# Gold‐Catalyzed Cycloisomerization of Sulfur Ylides to Dihydrobenzothiepines

**DOI:** 10.1002/chem.202000622

**Published:** 2020-06-08

**Authors:** Christian Knittl‐Frank, Iakovos Saridakis, Thomas Stephens, Rafael Gomes, James Neuhaus, Antonio Misale, Rik Oost, Alberto Oppedisano, Nuno Maulide

**Affiliations:** ^1^ Institute for organic Chemistry University of Vienna Währinger Strasse 38 1090 Vienna Austria

**Keywords:** alkynes, benzothiepines, gold, rearrangement, sulfonium ylides

## Abstract

The metal‐promoted nucleophilic addition of sulfur ylides to π‐systems is a well‐established reactivity. However, the driving force of such transformations, elimination of a sulfide moiety, entails stoichiometric byproducts making them unfavorable in terms of atom economy. In this work, a new take on sulfur ylide chemistry is reported, an atom‐economical gold(I)‐catalyzed synthesis of dihydrobenzo[*b*]thiepines. The reaction proceeds under mild conditions at room temperature.

## Graphical abstract

**Readily available** *S*‐homopropargyl sulfonium ylides are transformed to substituted dihydrobenzo[*b*]thiepines under Au^I^ catalysis. This atom‐efficient transformation takes place under very mild conditions at ambient temperature and proceeds through a charge‐accelerated sulfonium [3,3]‐sigmatropic rearrangement.

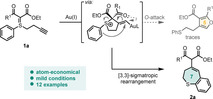

Six decades past the pioneering work of Johnson^1^, ^2^, ^3^ and Corey,^4^, ^5^ sulfonium and sulfoxonium ylides have arguably become textbook reagents in organic synthesis.^6^, ^7^, ^8^, ^9^, ^10^ In particular, they serve as one‐carbon synthons for the construction of small rings such as epoxides,^11^, ^12^, ^13^, ^14^, ^15^, ^16^, ^17^ cyclopropanes^11^, ^17^, ^18^, ^19^, ^20^ and aziridines^11^, ^17^, ^20^, ^21^, ^22^, ^23^ as well as for the synthesis of more complex cores through rearrangement reactions.^24^ Whilst the majority of the initial studies were based on non‐stabilized sulfonium and sulfoxonium ylides, modern work exploits the reactivity of their stabilized surrogates, in which electron density from the ylidic carbon is distributed to one or two electron‐withdrawing functionalities. This in turn enables the utilization of these sulfur ylides as practical and bench‐stable reagents as well as the development of novel reactivity. Indeed, applications of stabilized sulfonium and sulfoxonium ylides have been expanded to encompass noble transition metal catalysis,^8^, ^9^, ^25^ in particular Au^I^.

Recently, our group has reported that gold‐catalyzed electrophilic activation of alkenes allows the construction of highly functionalized cyclopropane scaffolds.^25^, ^26^, ^27^, ^28^, ^29^, ^30^ Prior to that, we^31^ and others^32^ have demonstrated the synthesis of multisubstituted furan cores through gold‐catalyzed activation of alkynes in the presence of sulfonium ylides, either in intra‐ or intermolecular fashion. Interestingly, the majority of these and related transition metal‐catalyzed reactions of sulfur ylides mostly employ variations on substituents tethered to the ylidic carbon. On the other hand, only minor variations on the sulfur substitution have been reported,^25^, ^30^ probably due to the fact that most of those transformations eventually result in elimination of the sulfur tether during the reaction, thus, leading to stoichiometric sulfide byproducts (cf. Scheme 1 a, ii–iv).

**Scheme 1:**
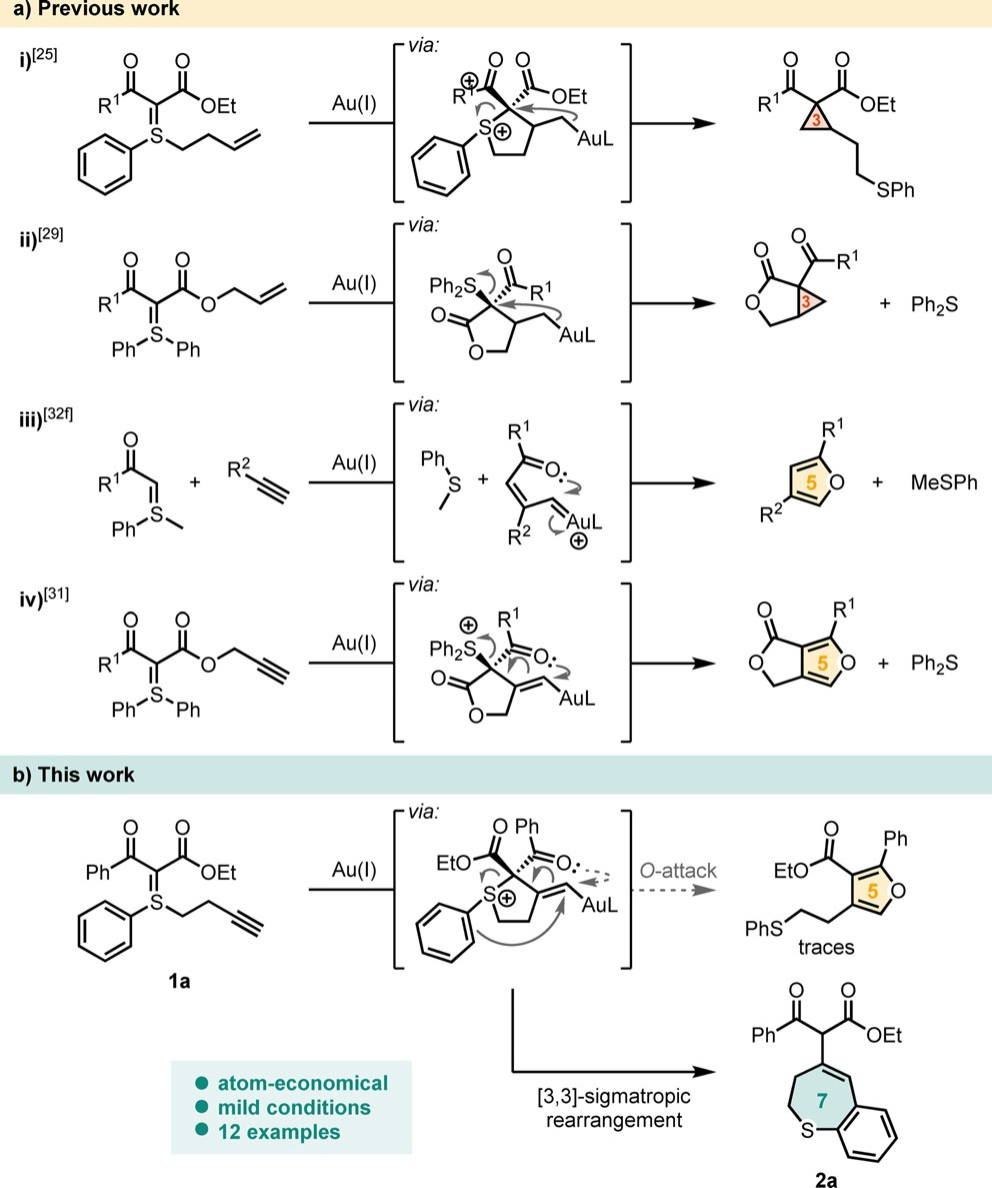
Reactivity of alkenes and alkynes with sulfonium ylides under gold(I) catalysis.

Given our recent experience in the cycloisomerization of *S*‐homoallyl sulfonium ylides (Scheme 1 a, i),^25^ we became interested in exploring the reactivity of analogous S‐homopropargyl sulfonium ylides. In this regard, initial experiments involved the reaction of the readily available sulfonium ylide **1 a** 33 with JohnPhos Au(MeCN)SbF_6_ (5 mol %) in MeOH at room temperature, aiming at the formation of a trisubstituted furan, inspired by a prior study of our group (Scheme 1 a iv).^31^ To our surprise, the obtained spectroscopic data for the major product were not in agreement with our hypothesis. Thorough analysis revealed that dihydrobenzo[*b*]thiepine **2 a** was formed as a major product in lieu of the anticipated furan (Scheme 1 b). This serendipitously discovered reactivity prompted us to investigate the scope of this atom‐economical transformation, especially since the benzothiepine scaffold is a known pharmacophore.^34^ Herein, we report on a gold‐catalyzed cycloisomerization of S‐homopropargyl sulfonium ylides to functionalized dihydrobenzo[*b*]thiepines.^35^

Whilst minute amounts (5 %) of the furan product could be detected in initial setups, the addition of silver triflate and employment of a binary solvent system (isopropanol/water 4:1 *v*/*v*) suppressed it entirely and was optimal for the dihydrobenzo[*b*]thiepine formation (see SI for details). Hence, **2 a** (along with its isomer **2 a’**) was isolated in 77 % yield.^36^

To investigate the scope of our reaction, we first screened variations in the northern 1,3‐dicarbonyl substituent. We found that electron‐neutral, ‐rich, and ‐poor aromatics were all well tolerated (Figure 1, **2 a**–**c**). Interestingly, the *p*‐NO_2_ substituted sulfonium ylide **1 c** required higher temperature (50 °C) than the rest of the substrates to reach full conversion. This might be ascribed to the decreased nucleophilicity of the ylidic carbon, due to better delocalization of its formal negative charge. Moreover, the aliphatic β‐keto esters **1 d** and **1 e** delivered the benzothiepine in satisfying yields, too.

**Figure 1:**
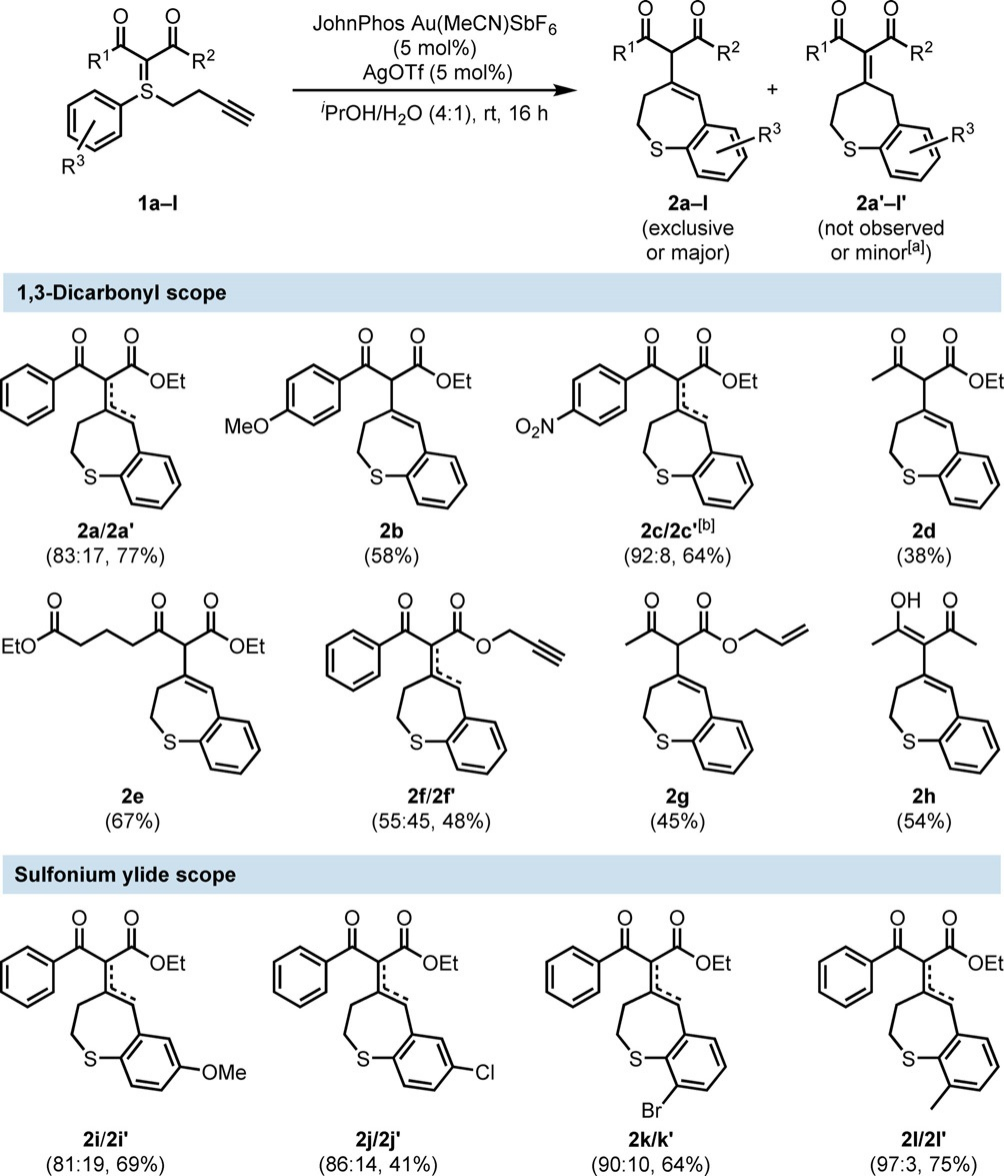
Scope of the Au^I^‐catalyzed dihydrobenzo[*b*]thiepine synthesis. All yields are isolated yields after column chromatography. Reactions were carried out in 0.2 mmol scale. [a] Formation of isomer **2’** resulted in an inseparable mixture. Given ratios determined by NMR after isolation. [b] Reaction at 50 °C.

We then devised a competition experiment by subjecting the sulfonium ylides **1 f** and **1 g**, bearing a propargyl and an allyl ester‐substituted ylidic C‐atom to our reaction conditions, respectively. In previously reported gold(I)‐catalyzed processes, propargyl‐ and allyl‐tethered diphenylsulfonium ylides were readily transformed into the corresponding furans^31^ and cyclopropanes,^29^ respectively. Following this reactivity, **1 f** would react to the corresponding furan **2 f’’** through an initial 5‐*exo*‐*dig* attack of the ylidic carbon onto the gold(I)‐activated triple bond followed by a 5‐*endo*‐*trig* cyclization along with sulfide release (Scheme 2 a).^31^ Likewise, **1 g** would form cyclopropane **2 g’’** after an initial 5‐*exo*‐*trig* attack of the ylidic C‐atom onto the gold(I)‐activated double bond with a subsequent 3‐*exo*‐*tet* cyclization concomitant with sulfide release (Scheme 2 b).^29^ In contrast to those reports, the presence of the *S*‐homopropargyl group completely shut down this pathway in both cases examined. Thus, under our reaction conditions we observed complete selectivity towards the formation of dihydrobenzo[*b*]thiepines, through an initial attack of the ylidic carbon onto the gold(I)‐activated S‐homopropargyl triple bond (cf. also Scheme 3). Hence, the reaction of **1 f** and **1 g** yielded **2 f**/**2 f’** and **2 g** with the propargyl and allyl moiety remaining intact, respectively.

**Scheme 2:**
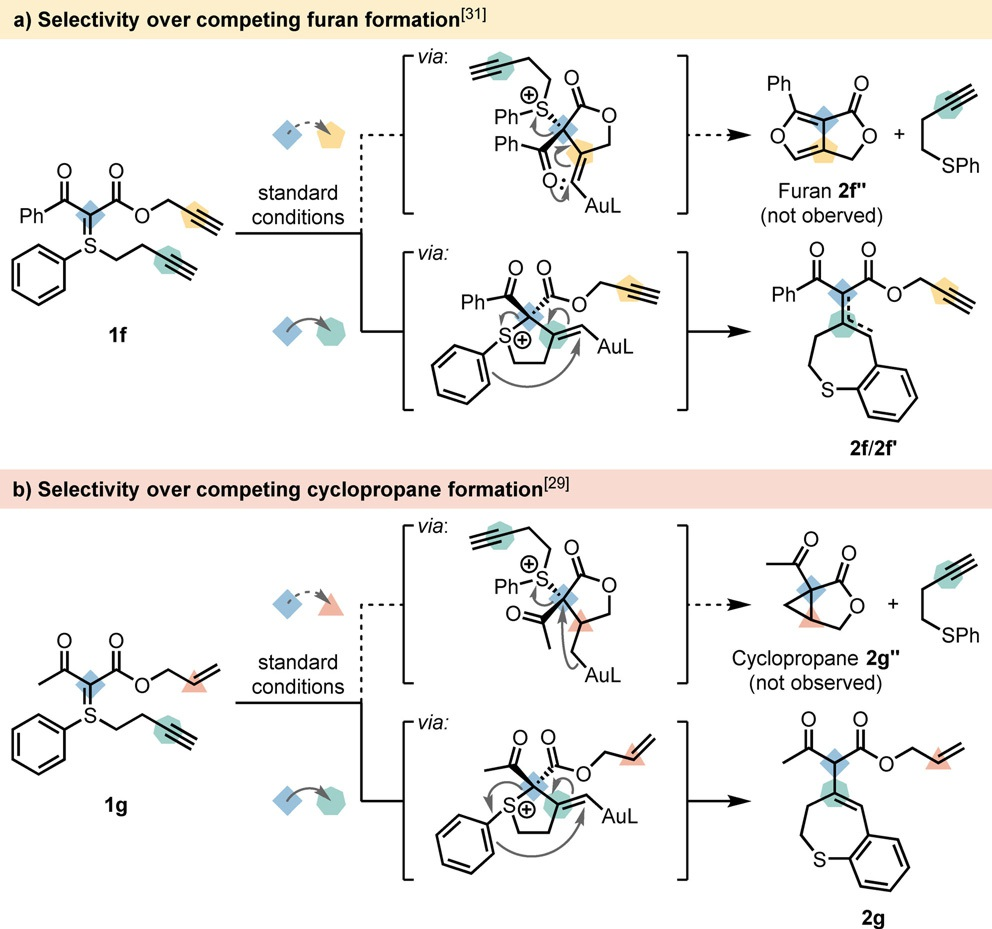
Probed chemoselectivity of propargylic and allylic esters **1 f** and **1 g**. Conditions as in Figure 1.

**Scheme 3:**
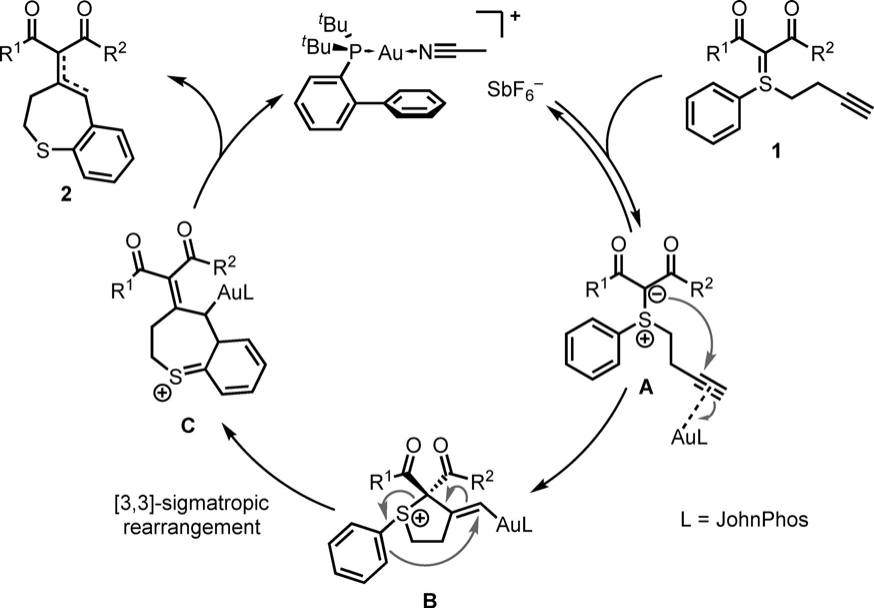
Proposed catalytic cycle for the formation of benzo[*b*]thiepines.

Furthermore, the acetylacetone derivative **1 h** afforded the corresponding benzo[*b*]thiepine in moderate yield. It is noteworthy that **2 h** resided solely in its enol form (in chloroform), whilst the rest of the synthesized dihydrobenzo[*b*]thiepines **2** were present in both tautomeric forms.

Finally, we surveyed the functionality tolerance on the southern domain of the sulfonium ylides. Hence, different *para*‐ and *ortho*‐substituted *S*‐aryl moieties were examined, and gratifyingly delivered the desired benzothiepines **2 i**–**l** in modest‐to‐good yields.

From a mechanistic point of view, we envisaged that the reaction starts with activation of the alkyne by π‐coordination to the gold catalyst,^37^ as shown in intermediate **A** (Scheme 3). An intramolecular 5‐*exo*‐*dig* attack of the ylidic carbon to the internal alkyne C‐atom leads to a gold‐vinyl complex **B**,^38^ which undergoes a charge‐accelerated sulfonium [3,3]‐sigmatropic re‐arrangement^24^, 35c, 39 to furnish the seven‐membered thiepine ring **C**. Finally, rearomatization and protodeauration^40^ deliver the dihydrobenzo[*b*]thiepines **2**, completing the catalytic cycle.

In conclusion, a Au^I^‐catalyzed synthesis of dihydrobenzo[*b*]thiepines was developed. The mild conditions of the cycloisomerization and the simple accessibility of the sulfonium ylide starting materials are distinctive characteristics of this process. Interestingly, this process appears to supersede the previously reported Au^I^‐catalyzed intramolecular furan and cyclopropane syntheses. The retention of the sulfur atom onto the final product after the reaction is an unusual trait of metal‐catalyzed transformations of sulfonium ylides and enables the development of atom‐economical synthetic transformations.

## Conflict of interest

The authors declare no conflict of interest.

## Supporting information

As a service to our authors and readers, this journal provides supporting information supplied by the authors. Such materials are peer reviewed and may be re‐organized for online delivery, but are not copy‐edited or typeset. Technical support issues arising from supporting information (other than missing files) should be addressed to the authors.

Supplementary
