# Supplementary material for: Gold‐Catalyzed Cycloisomerization of Sulfur Ylides to Dihydrobenzothiepines
Source: Chemistry. 2020 Jun 8;26(48):10972–5. doi: 10.1002/chem.202000622 (PMC7496544; doi:10.1002/chem.202000622)
Supplement: Supplementary file 1 — Supplementary [file CHEM-26-10972-s001.pdf]

Chemistry–A European Journal

Supporting Information

Gold-Catalyzed Cycloisomerization of Sulfur Ylides to Dihydrobenzothiepines

Christian Knittl-Frank, Iakovos Saridakis, Thomas Stephens, Rafael Gomes, James Neuhaus, Antonio Misale, Rik Oost, Alberto Oppedisano, and Nuno Maulide*[a]

Contents

1. General remarks 2

2. Synthesis of thioethers 3a–e 2

General procedure for the thioether synthesis (GP1) 2

3. Synthesis of sulfoxides 4a–e 3

General procedure for the sulfoxide synthesis (GP2) 3

4. Synthesis of ketoester 6 5

5. Synthesis of sulfonium ylides 1a–l 5

General procedure for the sulfonium ylide synthesis (GP3) 5

6. Synthesis of dihydrobenzothiepines 2a–l 9

Condition screening 9

General procedure for the Au(I)-catalyzed dihydrobenzothiepine synthesis (GP4) 10

7. References 16

8. NMR spectra 17

1. General remarks

All reactions were carried out under an argon atmosphere using oven-dried glassware and using standard Schlenk techniques. All reagents were used as received from commercial suppliers unless otherwise stated. Neat infrared spectra were recorded using a Perkin-Elmer Spectrum 100 FT-IR spectrometer. Wavenumbers ($\upsilon = 1/\lambda$) are reported in cm^{-1}. Mass spectra were acquired in positive ion mode by use of an ESI-Qq-TOF mass spectrometer (Bruker maXis UHR-TOF) or an EI-Q-TOF (Agilent Technologies 7200 Accurate-Mass Q-TOF GC/MS). All ^{1}H-NMR and ^{13}C-NMR experiments were recorded using Bruker AV-400, AV-600 or AV-700, spectrometers at 300 K. Chemical shifts (δ) are quoted in ppm and coupling constants (J) are quoted in Hz. The 7.26 ppm resonance of residual $CHCl_3$ for ^{1}H spectra and the 77.16 ppm resonance for ^{13}C spectra were used as internal references. Reaction progress was monitored by thin layer chromatography (TLC) performed on aluminum plates coated with silica gel F_{254} with 0.2 mm thickness. Visualization was achieved by a combination of ultraviolet light (254 nm) and potassium permanganate. Flash column chromatography was performed using silica gel 60 (230–400 mesh, Merck and co.).

2. Synthesis of thioethers 3a–e

General procedure for the thioether synthesis (GP1)

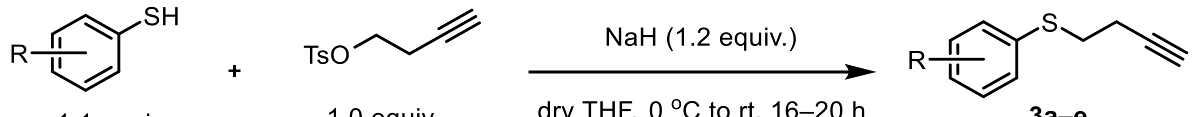

To a solution of thiophenol derivative (1.1 equiv.) in dry THF (2 ml mmol^{-1}) under argon atmosphere was added NaH (1.2 equiv.) at 0 °C. The mixture was stirred at 0 °C for 30 min and a solution of 3-butynyl *p*-toluenesulfonate (1 equiv.) in dry THF (4 ml mmol^{-1}). The mixture was warmed to room temperature and stirred further for 16–20 h. After completion, the excess of NaH was quenched by addition H_2O at 0 °C, the mixture was extracted with Et_2O, the combined organic layers were washed with brine, dried over $MgSO_4$. After removal of volatiles under reduced pressure, purification *via* flash chromatography (silica gel, EtOAc(0⟶5%)/heptane) afforded the desired thioether **3**.

But-3-yn-1-yl(phenyl)sulfane (3a)

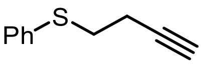

Synthesized according to **GP1** from commercially available thiophenol (82.5 mmol, 9.09 g). 87%, colorless oil.

Spectral data are in accordance to the literature.[1](Compound 28)

But-3-yn-1-yl(4-methoxyphenyl)sulfane (3b)

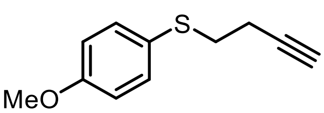

Synthesized according to **GP1** from commercially available 4-methoxybenzenethiol (15.4 mmol, 2.23 g). 75%, colorless oil.

^{1}H NMR (600 MHz, $CDCl_3$) δ 7.41–7.36 (m, 2H), 6.88–6.82 (m, 2H), 3.80 (s, 3H), 2.95 (t, J = 7.4 Hz, 2H), 2.42 (td, J = 7.5, 2.6 Hz, 2H), 2.02 (t, J = 2.6 Hz, 1H); **^{13}C NMR (151 MHz, $CDCl_3$)** δ 159.5 (C), 134.3 (2 CH), 125.2 (C), 114.8 (2 CH), 82.6 (C), 69.6 (CH), 55.5 (CH_3), 35.0 (CH_2), 19.5 (CH_2); **FTIR (neat, cm^{-1}):** 3292, 2954, 2922, 2853, 1592, 1493, 1462, 1284, 1244, 1173, 1032; **HRMS (EI+)**: m/z: $[M]^+$ Calcd. for $C_{11}H_{12}OS^+$ 192.0603; Found 192.0595.

But-3-yn-1-yl(4-chlorophenyl)sulfane (3c)

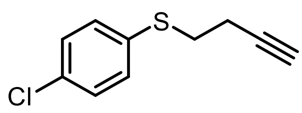

Synthesized according to **GP1** from commercially available 4-chlorobenzenethiol (11 mmol, 1.59 g). Quantitative yield, yellow oil.

^{1}H NMR (600 MHz, CDCl$_3$) δ 7.33–7.24 (m, 4H), 3.05 (t, J = 7.4 Hz, 2H), 2.47 (td, J = 7.4, 2.6 Hz, 2H), 2.04 (t, J = 2.6 Hz, 1H); **^{13}C NMR (151 MHz, CDCl$_3$)** δ 133.9 (C), 132.8 (C), 131.6 (2 CH), 129.3 (2 CH), 82.1 (C), 70.0 (CH), 33.3 (CH$_2$), 19.4 (CH$_2$); **FTIR (neat, cm^{-1}):** 3299, 3066, 2929, 2852, 2119, 1476, 1095, 1012; **HRMS (EI+)** m/z: [M]$^+$ Calcd. for $C_{10}H_9ClS^+$ 196.0108; Found 196.0107.

(2-Bromophenyl)(but-3-yn-1-yl)sulfane (3d)

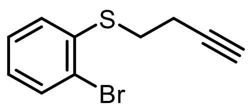

Synthesized according to **GP1** from commercially available 2-bromobenzenethiol (11 mmol, 2.08 g). Quantitative yield, yellow oil.

^{1}H NMR (600 MHz, CDCl$_3$) δ 7.57 (dd, J = 7.9, 1.3 Hz, 1H), 7.33–7.25 (m, 2H), 7.06 (ddd, J = 8.0, 7.1, 1.9 Hz, 1H), 3.12 (t, J = 7.6 Hz, 2H), 2.55 (td, J = 7.6, 2.7 Hz, 2H), 2.07 (t, J = 2.7 Hz, 1H); **^{13}C NMR (151 MHz, CDCl$_3$)** δ 136.9 (C), 133.4 (CH), 129.1 (CH), 128.0 (CH), 127.4 (CH), 124.6 (C), 82.0 (C), 70.1 (CH), 32.0 (CH$_2$), 19.0 (CH$_2$); **FTIR (neat, cm^{-1}):** 3294, 3058, 2931, 2834, 2118, 1448, 1427, 1041; **HRMS (EI+)** m/z: [M]$^+$ Calcd. for $C_{10}H_9BrS^+$ 239.9603; Found 239.9591.

But-3-yn-1-yl(o-tolyl)sulfane (3e)

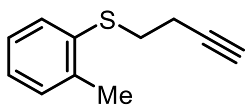

Synthesized according to **GP1** from commercially available 2-methylbenzenethiol (11 mmol, 1.37 g). Quantitative yield, yellow oil.

^{1}H NMR (600 MHz, CDCl$_3$) δ 7.32 (dd, J = 7.7, 1.4 Hz, 1H), 7.20–7.11 (m, 3H), 3.05 (t, J = 7.5 Hz, 2H), 3.05 (td, J = 7.5, 2.7 Hz, 2H), 2.40 (s, 3H), 2.05 (t, J = 2.7 Hz, 1H); **^{13}C NMR (151 MHz, CDCl$_3$)** δ 138.6 (C), 134.6 (C), 130.5 (CH), 129.2 (CH), 126.6 (CH), 126.5 (CH), 82.5 (C), 69.8 (CH), 32.3 (CH$_2$), 20.6 (CH$_3$), 19.3 (CH$_2$); **FTIR (neat, cm^{-1}):** 3293, 3060, 3011, 2969, 2931, 2844, 2118, 1469, 1457, 1434, 1048, 1035; **HRMS (EI+)** m/z: [M]$^+$ Calcd. for $C_{11}H_{12}S^+$ 176.0654; Found 176.0648.

3. Synthesis of sulfoxides 4a–e

General procedure for the sulfoxide synthesis (GP2)

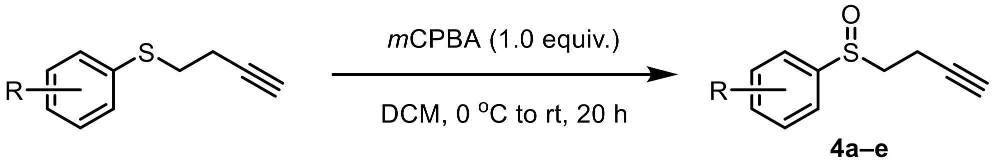

To a solution of the corresponding thioether **3** (1.0 equiv.) in DCM (4 ml mmol^{-1}) was added *m*CPBA (1.0 equiv.) at 0 °C. The mixture was stirred at room temperature for 20 h before it was washed with a saturated solution of $NaHCO_3$ in H_2O. The aqueous phase was extracted with DCM and the combined organic phases were dried over $MgSO_4$. After removal of volatiles under reduced pressure, purification *via* flash chromatography (silica gel, EtOAc(10⟶40%)/heptane) afforded the desired sulfoxide **4**.

(But-3-yn-1-ylsulfinyl)benzene (4a)

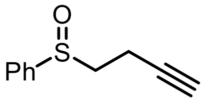

Synthesized according to **GP2** from thioether **3a** (85 mmol, 13.79 g). 80%, colorless oil.

Spectral data are in accordance to the literature.[2](Compound 1a)

1-(But-3-yn-1-ylsulfinyl)-4-methoxybenzene (4b)

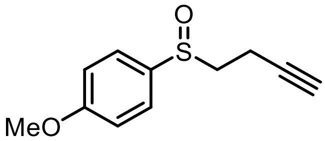

Synthesized according to **GP2** from thioether **3b** (11.8 mmol, 2.27 g). 73%, colorless oil.

^{1}H NMR (600 MHz, CDCl$_3$) δ 7.59–7.55 (m, 2H), 7.06–7.01 (m, 2H), 3.86 (s, 3H), 2.98–2.90 (m, 2H), 2.72–2.64 (m, 1H), 2.41 (dddd, J = 17.1, 7.9, 6.3, 2.7 Hz, 1H), 2.03 (t, J = 2.7 Hz, 1H); **^{13}C NMR (151 MHz, CDCl$_3$)** δ 162.3 (C), 133.9 (C), 126.1 (2 CH), 115.0 (2 CH), 80.9 (C), 70.5 (CH$_2$), 55.7 (CH$_3$), 55.4 (CH$_2$), 12.3 (CH$_2$); **FTIR (neat, cm^{-1}):** 3291, 3223, 2841, 2838, 1593, 1495, 1303, 1247, 1172, 1087, 1022; **HRMS (ESI+)**: m/z: [M+Na]$^+$ Calcd. for $C_{11}H_{12}NaO_2S^+$ 231.0450; Found 231.0446.

1-(But-3-yn-1-ylsulfinyl)-4-chlorobenzene (4c)

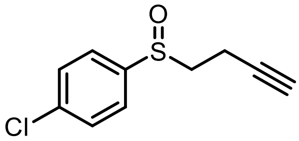

Synthesized according to **GP2** from thioether **3c** (10 mmol, 1.97 g). 94%, colorless solid.

^{1}H NMR (600 MHz, CDCl$_3$) δ 7.59–7.56 (m, 2H), 7.54–7.50 (m, 2H), 3.00 (ddd, J = 13.1, 8.2, 7.2 Hz, 1H), 2.94–2.88 (m, 1H), 2.76–2.69 (m, 1H), 2.46–2.39 (m, 1H), 2.04 (t, J = 2.7 Hz, 1H); **^{13}C NMR (151 MHz, CDCl$_3$)** δ 141.7 (C), 137.7 (C), 129.8 (2 CH), 125.6 (2 CH), 80.5 (C), 70.8 (CH), 55.4 (CH$_2$), 12.1 (CH$_2$); **FTIR (neat, cm^{-1}):** 3300, 3233, 2923, 1575, 1476, 1431, 1392, 1091, 1078, 1047, 1011; **HRMS (ESI+)**: m/z: [M+Na]$^+$ Calcd. for $C_{10}H_9ClNaOS^+$ 234.9955; Found 234.9965.

1-Bromo-2-(but-3-yn-1-ylsulfinyl)benzene (4d)

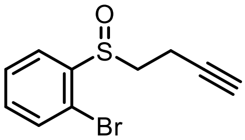

Synthesized according to **GP2** from thioether **3d** (9.01 mmol, 2.17 g). 93%, colorless solid.

^{1}H NMR (600 MHz, CDCl$_3$) δ 7.89–7.84 (m, 1H), 7.57 (dd, J = 11.7, 4.3 Hz, 2H), 7.39 (td, J = 7.6, 1.7 Hz, 1H), 3.32 (ddd, J = 13.2, 8.8, 7.1 Hz, 1H), 3.00–2.94 (m, 1H), 2.80 (dddd, J = 17.1, 8.8, 7.1, 2.7 Hz, 1H), 2.47 (dddd, J = 17.1, 8.8, 5.2, 2.7 Hz, 1H), 2.00 (t, J = 2.7 Hz, 1H); **^{13}C NMR (151 MHz, CDCl$_3$)** δ 142.6 (C), 133.2 (CH), 132.6 (CH), 128.6 (CH), 126.9 (CH), 118.8 (C), 80.7 (C), 70.4 (CH), 52.1 (CH$_2$), 11.9 (CH$_2$); **FTIR (neat, cm^{-1}):** 3297, 3233, 3059, 2929, 1567, 1447, 1431, 1244, 1095, 1056, 1015; **HRMS (ESI+)**: m/z: [M + Na]$^+$ Calcd. for $C_{10}H_9BrNaOS^+$ 278.9450; Found 278.9458.

1-(But-3-yn-1-ylsulfinyl)-2-methylbenzene (4e)

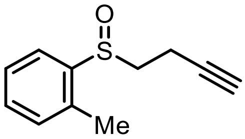

Synthesized according to **GP2** from thioether **3e** (8.72 mmol, 1.54 g). 86%, colorless solid.

^{1}H NMR (600 MHz, CDCl$_3$) δ 7.88 (dd, J = 7.7, 1.4 Hz, 1H), 7.46–7.37 (m, 2H), 7.21 (d (broad), J = 7.4 Hz, 1H), 3.01 (ddd, J = 12.2, 8.3, 7.1 Hz, 1H), 2.87–2.74 (m, 2H), 2.52–2.44 (m, 1H), 2.39 (s, 3H), 2.03 (t, J = 2.6 Hz, 1H); **^{13}C NMR (151 MHz, CDCl$_3$)** δ 141.4 (C), 134.6 (C), 131.1 (CH), 131.0 (CH), 127.4 (CH), 124.0 (CH), 81.0 (C), 70.4 (CH), 53.4 (CH$_2$), 18.2 (CH$_3$),

12.4 (CH_2); **FTIR (neat, cm⁻¹):** 3293, 3227, 3050, 2922, 2855, 1471, 1066, 1034; **HRMS (ESI+)**: m/z: [M + Na]$^+$ Calcd. for $C_{11}H_{12}NaOS^+$ 215.0501; Found 215.0509.

4. Synthesis of ketoester 6

3-oxo-3-phenylpropanoic acid (5)

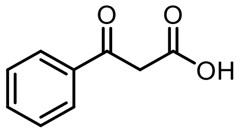

Prepared from commercially available ethyl benzoylacetate (25 mmol, 4.81 g) following a literature procedure.[3] 80%, colorless solid.

Spectral data are in accordance to the literature.[3](Compound S28)

Prop-2-yn-1-yl 3-oxo-3-phenylpropanoate (6)

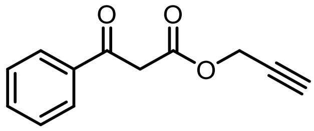

Prepared from **5** (15 mmol, 2.46 g) and 2-propyn-1-ol following a literature procedure.[4] 57%, colorless oil.

Spectral data are in accordance to the literature.[5](Compound 3c)

5. Synthesis of sulfonium ylides 1a–l

General procedure for the sulfonium ylide synthesis (GP3)

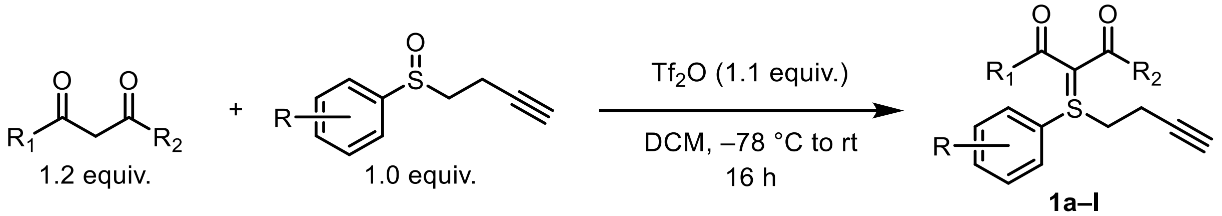

The corresponding 1,3-dicarbonyl compound (1.2 equiv.) and the sulfoxide **4** (1.0 equiv.) were dissolved in anhydrous DCM (1 ml mmol^{-1}) under an argon atmosphere. The solution was cooled to –78 °C and trifluoromethanesulfonic anhydride (1.1 equiv.) was added dropwise. The reaction mixture was stirred for 12 h at this temperature and allowed to warm up to room temperature. After 4 h stirring at room temperature, the resulting solution was quenched with a half saturated Na_2CO_3 solution. Water was added and the aqueous layer was extracted with DCM. The combined organic layers were dried over $MgSO_4$ and the solvent was evaporated *in vacuo*. The crude mixture was purified by flash chromatography (silica gel, EtOAc(30⟶80%)/heptane) to afford the desired sulfonium ylide **1**.

Ethyl 2-(but-3-yn-1-yl(phenyl)-l4-sulfaneylidene)-3-oxo-3-phenylpropanoate (1a)

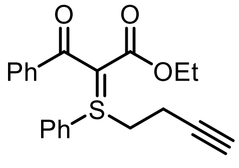

Synthesized according to **GP3** from sulfoxide **4a** (7 mmol, 1.25 g) and the corresponding commercially available β-keto ester. 65%, white solid.

^{1}H NMR (600 MHz, CDCl$_3$) δ 7.83–7.81 (m, 2H), 7.51–7.47 (m, 5H), 7.37–7.30 (m, 3H), 4.51–4.47 (m, 1H), 3.96–3.90 (m, 2H), 3.70–3.66 (m, 1H), 2.70–2.68 (m, 2H), 2.16 (t, J = 2.6 Hz, 1H), 0.90 (t, J = 7.1 Hz, 3H); **^{13}C NMR (151 MHz, CDCl$_3$)** δ 190.8 (C), 166.8 (C), 143.1 (C), 131.5 (CH), 131.1 (C), 129.8 (2 CH), 129.2 (CH), 128.4 (2 CH), 127.4 (2 CH), 127.3 (2 CH), 79.3 (C), 71.8 (CH), 71.3 (CH), 59.4 (CH_2), 40.9 (CH_2), 15.2 (CH_2), 13.9 (CH_3); **FTIR (neat, cm^{-1}):** 3289, 3239, 3058,

2978, 1646 1585, 1549, 1477, 1443, 1366, 1325, 1273, 1157, 1127, 1057, 1025; **HRMS (ESI+)**: m/z: $[M+Na]^+$ Calcd. for $C_{21}H_{20}O_3SNa^+$ 375.1025; Found 375.1026.

Ethyl 2-(but-3-yn-1-yl(phenyl)-l4-sulfaneylidene)-3-(4-methoxyphenyl)-3-oxopropanoate (1b)

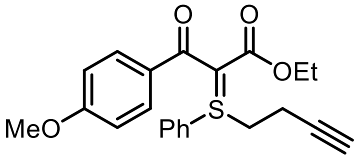

Synthesized according to **GP3** from sulfoxide **4a** (7 mmol, 1.25 g) and the corresponding commercially available β-keto ester. 69%, orange highly viscous oil.

^{1}H NMR (600 MHz, CDCl$_3$) δ 7.82–7.81 (m, 2H), 7.57–7.49 (m, 5H), 6.85–6.84 (m, 2H), 4.50–4.46 (m, 1H), 4.01–3.97 (m, 2H), 3.83 (s, 3H), 3.69–3.64 (m, 1H), 2.69 (ddd, J = 7.6, 6.4, 2.7 Hz, 2H), 2.15 (t, J = 2.6 Hz, 1H), 1.00 (t, J = 7.1 Hz, 3H); **^{13}C NMR (151 MHz, CDCl$_3$)** δ 190.0 (C), 166.9 (C), 161.0 (C), 135.2 (C), 131.49 (C), 131.46 (CH), 130.0 (2 CH), 129.9 (2 CH), 128.4 (2 CH), 112.6 (2 CH), 79.5 (C, HSQC), 71.8 (CH), 70.5 (C), 59.4 (CH_2), 55.3 (CH_3), 41.1 (CH_2), 15.3 (CH_2), 14.2 (CH_3); **FTIR (neat, cm^{-1}):** 3290, 3245, 3057, 2978, 2839, 1647, 1604, 1584, 1552, 1509, 1444, 1366, 1328, 1279, 1250, 1173, 1062, 1029; **HRMS (ESI+)**: m/z: $[M+Na]^+$ Calcd. for $C_{22}H_{22}O_4SNa^+$ 405.1131; Found 405.1132.

Ethyl 2-(but-3-yn-1-yl(phenyl)-l4-sulfaneylidene)-3-(4-nitrophenyl)-3-oxopropanoate (1c)

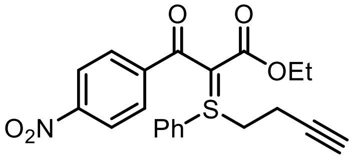

Synthesized according to **GP3** from sulfoxide **4a** (7 mmol, 1.25 g) and the corresponding commercially available β-keto ester. 68%, orange highly viscous oil.

^{1}H NMR (600 MHz, CDCl$_3$) δ 8.19 (d, J = 8.8 Hz, 2H), 7.87–7.85 (m, 2H), 7.59–7.53 (m, 5H), 4.51–4.47 (m, 1H), 3.98–3.91 (m, 2H), 3.80–3.76 (m, 1H), 2.73–2.70 (m, 2H), 2.19 (t, J = 2.6 Hz, 1H), 0.93 (t, J = 7.1 Hz, 3H); **^{13}C NMR (151 MHz, CDCl$_3$)** δ 188.4 (C), 166.1 (C), 149.5 (C), 147.8 (C), 132.1 (CH), 130.3 (C), 130.1 (2 CH), 128.8 (2 CH), 128.0 (2 CH), 122.9 (2 CH), 79.0 (C, HSQC), 73.0 (C), 72.2 (CH), 59.8 (CH_2), 40.8 (CH_2), 15.4 (CH_2), 14.0 (CH_3); **FTIR (neat, cm^{-1}):** 3289, 2980, 1668, 1559, 1519, 1334, 1282, 1062; **HRMS (ESI+)**: m/z: $[M+Na]^+$ Calcd. for $C_{21}H_{19}O_5NSNa^+$ 420.0876; Found 420.0882.

Ethyl 2-(but-3-yn-1-yl(phenyl)-l4-sulfaneylidene)-3-oxobutanoate (1d)

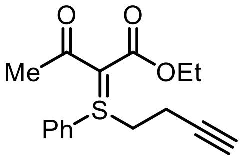

Synthesized according to **GP3** from sulfoxide **4a** (7 mmol, 1.25 g) and the corresponding commercially available β-keto ester. 60%, orange solid.

^{1}H NMR (600 MHz, CDCl$_3$) δ 7.69–7.68 (m, 2H), 7.49–7.47 (m, 3H), 4.45–4.41 (m, 1H), 4.21–4.11 (m, 2H), 3.58 (ddd, J = 11.6, 8.3, 7.0 Hz, 1H), 2.65–2.54 (m, 2H), 2.47 (s, 3H), 2.13 (t, J = 2.7 Hz, 1H), 1.26 (t, J = 7.1 Hz, 3H); **^{13}C NMR (151 MHz, CDCl$_3$)** δ 192.5 (C), 167.2 (C), 131.4 (CH), 131.2 (C), 129.9 (2 CH), 128.3 (2 CH), 79.6 (C, HSQC), 71.7 (CH), 71.2 (C), 59.6 (CH_2), 40.7 (CH_2), 30.2 (CH_3), 15.3 (CH_2), 14.8 (CH_3); **FTIR (neat, cm^{-1}):** 3292, 3247, 2979, 1736, 1664, 1573, 1478, 1444, 1419, 1368, 1323, 1276, 1236, 1167, 1069, 1032; **HRMS (ESI+)**: m/z: $[M+Na]^+$ Calcd. for $C_{16}H_{18}O_3SNa^+$ 313.0869; Found 313.0868.

Diethyl 2-(but-3-yn-1-yl(phenyl)-l4-sulfaneylidene)-3-oxoheptanedioate (1e)

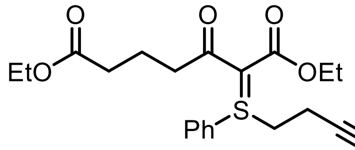

Synthesized according to **GP3** from sulfoxide **4a** (2 mmol, 357 mg) and the corresponding commercially available β-keto ester. 53%, colorless oil.

^{1}H NMR (600 MHz, CDCl$_3$) δ 7.66–7.64 (m, 2H), 7.47–7.44 (m, 3H), 4.38 (ddd, J = 12.2, 6.9, 5.6 Hz, 1H), 4.18–4.07 (m, 4H), 3.58 (ddd, J = 11.6, 8.5, 6.9 Hz, 1H), 2.94–2.88 (m, 2H), 2.62–2.51 (m, 2H), 2.32–2.30 (m, 2H), 2.12 (t, J = 2.7 Hz, 1H), 1.93–1.88 (m, 2H), 1.24–1.20 (m, 6H); **^{13}C NMR (151 MHz, CDCl$_3$)** δ 194.0 (C), 173.8 (C), 166.9 (C), 131.3 (CH), 131.2 (C), 129.8 (2 CH), 128.1 (2 CH), 79.6 (C, HSQC), 71.7 (CH), 70.9 (C), 60.2 (CH$_2$), 59.5 (CH$_2$), 40.7 (CH$_2$), 40.3 (CH$_2$), 34.2 (CH$_2$), 21.0 (CH$_2$), 15.2 (CH$_2$), 14.7 (CH$_3$), 14.3 (CH$_3$); **FTIR (neat, cm^{-1}):** 3253, 2978, 2933, 1726, 1663, 1574, 1443, 1370, 1276, 1167, 1061; **HRMS (ESI+)**: m/z: [M+Na]$^+$ Calcd. for $C_{21}H_{26}O_5SNa^+$ 413.1393; Found 413.1399.

Prop-2-yn-1-yl 2-(but-3-yn-1-yl(phenyl)-l4-sulfaneylidene)-3-oxo-3-phenylpropanoate (1f)

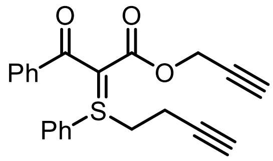

Synthesized according to **GP3** from sulfoxide **4a** (1 mmol, 178 mg) and β-keto ester **6**. 78%, slightly yellow highly viscous oil.

^{1}H NMR (600 MHz, CDCl$_3$) δ 7.84–7.83 (m, 2H), 7.53–7.51 (m, 5H), 7.38–7.33 (m, 3H), 4.54 (s, 2H), 4.53–4.49 (m, 1H), 3.71 (dt, J = 11.8, 7.6 Hz, 1H), 2.72–2.70 (m, 2H), 2.32 (t, J = 2.4 Hz, 1H), 2.18 (d, J = 2.6 Hz, 1H); **^{13}C NMR (151 MHz, CDCl$_3$)** δ 191.1 (C), 165.8 (C), 142.5 (C), 131.9 (CH), 131.0 (C), 130.1 (2 CH), 129.9 (CH), 128.7 (2 CH), 127.9 (2 CH), 127.6 (2 CH), 79.3 (C, HSQC), 78.7 (C, HSQC), 74.0 (CH), 72.2 (CH), 70.9 (C), 51.1 (CH$_2$), 41.2 (CH$_2$), 15.4 (CH$_2$); **FTIR (neat, cm^{-1}):** 3291, 3060, 3024, 2923, 2852, 2121, 1678, 1587, 1556, 1444, 1369, 1327, 1274, 1052; **HRMS (ESI+)**: m/z: [M+Na]$^+$ Calcd. for $C_{22}H_{18}O_3SNa^+$ 385.0869; Found 385.0871.

Allyl 2-(but-3-yn-1-yl(phenyl)-l4-sulfaneylidene)-3-oxobutanoate (1g)

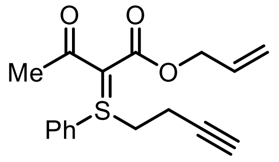

Synthesized according to **GP3** from sulfoxide **4a** (7 mmol, 1.25 g) and the corresponding commercially available β-keto ester. 44%, orange solid.

^{1}H NMR (600 MHz, CDCl$_3$) δ 7.68–7.66 (m, 2H), 7.48–7.43 (m, 3H), 5.91 (ddt, J = 17.1, 10.6, 5.4 Hz, 1H), 5.30–5.26 (m, 1H), 5.15 (dd, J = 10.5, 1.4 Hz, 1H), 4.60 (dddt, J = 37.6, 13.7, 5.4, 1.4 Hz, 2H), 4.42 (ddd, J = 12.1, 6.7, 5.7 Hz, 1H), 3.57 (ddd, J = 11.7, 8.4, 6.9 Hz, 1H), 2.62–2.51 (m, 2H), 2.46 (s, 3H), 2.12 (t, J = 2.6 Hz, 1H); **^{13}C NMR (151 MHz, CDCl$_3$)** δ 192.6 (C), 166.8 (C), 133.5 (CH), 131.5 (CH), 131.0 (C), 129.9 (2 CH), 128.3 (2 CH), 117.1 (CH$_2$), 79.6 (C, HSQC), 71.9 (CH), 70.9 (C), 64.4 (CH$_2$), 40.7 (CH$_2$), 30.2 (CH$_3$), 15.4 (CH$_2$); **FTIR (neat, cm^{-1}):** 3294, 3245, 2930, 1668, 1575, 1443, 1420, 1364, 1321, 1270, 1232, 1103, 1060, 1025; **HRMS (ESI+)**: m/z: [M+Na]$^+$ Calcd. for $C_{19}H_{18}O_3SNa^+$ 325.0869; Found 325.0872.

3-(But-3-yn-1-yl(phenyl)-l4-sulfaneylidene)pentane-2,4-dione (1h)

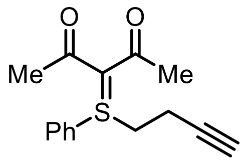

Synthesized according to **GP3** from sulfoxide **4a** (7 mmol, 1.25 g) and the corresponding commercially available 1,3-diketone. 69%, brown solid.

^{1}H NMR (600 MHz, CDCl$_3$) δ 7.66–7.64 (m, 2H), 7.50–7.49 (m, 3H), 4.59–4.55 (m, 1H), 3.58–3.53 (m, 1H), 2.70–2.65 (m, 1H), 2.57–2.51 (m, 1H), 2.45 (s, 6H), 2.19 (t, J = 2.6 Hz, 1H); **^{13}C NMR (151 MHz, CDCl$_3$)** δ 191.7 (2 C), 131.5 (CH), 130.7 (C), 130.0 (2 CH), 127.9 (2 CH), 79.9 (C), 72.2 (CH), 41.3 (CH$_2$), 30.4 (2 CH$_3$), 15.2 (CH$_2$); **FTIR (neat, cm^{-1}):** 3464, 3221,

3060, 2998, 2922, 1606, 1572, 1477, 1443, 1418, 1363, 1319, 1282, 1236, 1024; **HRMS (ESI+)**: m/z: $[M+Na]^+$ Calcd. for $C_{15}H_{16}O_2SNa^+$ 283.0763; Found 283.0761.

Ethyl 2-(but-3-yn-1-yl(4-methoxyphenyl)-l4-sulfaneylidene)-3-oxo-3-phenylpropanoate (1i)

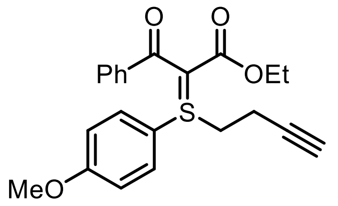

Synthesized according to **GP3** from sulfoxide **4b** (6.57 mmol, 2.44 g) and the corresponding commercially available β-keto ester. 22%, colorless oil.

^{1}H NMR (600 MHz, CDCl$_3$) δ 7.88–7.83 (m, 2H), 7.48–7.42 (m, 2H), 7.35–7.27 (m, 3H), 6.99–6.94 (m, 2H), 4.44–4.36 (m, 1H), 3.91 (q, J = 7.1 Hz, 2H), 3.81 (s, 3H), 3.70 (dt, J = 11.9, 7.4 Hz, 1H), 2.67–2.59 (m, 2H), 2.14 (t, J = 2.6 Hz, 1H), 0.88 (t, J = 7.1 Hz, 3H); **^{13}C NMR (151 MHz, CDCl$_3$)** δ 190.8 (C), 167.0 (C), 162.5 (C), 143.5 (C), 131.3 (2 CH), 129.2 (CH), 127.5 (2 CH), 127.4 (2 CH), 121.8 (C), 115.4 (2 CH), 79.5 (C), 73.5 (C), 71.8 (CH), 59.4 (CH_2), 55.6 (CH_2), 41.1 (CH_3), 15.4 (CH_2), 14.0 (CH_3); **FTIR (neat, cm^{-1}):** 3463, 3291, 3059, 2978, 2927, 2840, 1645, 1588, 1547, 1495, 1461, 1443, 1412, 1366, 1327, 1277, 1253, 1176, 1127, 1058, 1024; **HRMS (ESI+)**: m/z: $[M+Na]^+$ Calcd. for $C_{22}H_{22}O_4SNa^+$ 405.1131; Found 405.1133.

Ethyl 2-(but-3-yn-1-yl(4-chlorophenyl)-l4-sulfaneylidene)-3-oxo-3-phenylpropanoate (1j)

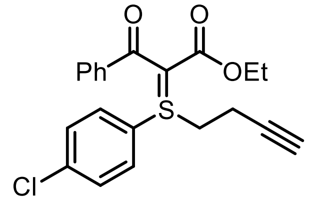

Synthesized according to **GP3** from sulfoxide **4c** (9.36 mmol, 1.99 g) and the corresponding commercially available β-keto ester. 99%, yellow highly viscous oil.

^{1}H NMR (600 MHz, CDCl$_3$) δ 7.82–7.80 (m, 2H), 7.49–7.47 (m, 4H), 7.38–7.31 (m, 3H), 4.51–4.47 (m, 1H), 3.97–3.91 (m, 2H), 3.69 (dt, J = 11.7, 7.6 Hz, 1H), 2.71–2.68 (m, 2H), 2.17 (t, J = 2.6 Hz, 1H), 0.91 (t, J = 7.1 Hz, 3H); **^{13}C NMR (151 MHz, CDCl$_3$)** δ 191.0 (C), 166.8 (C), 143.0 (C), 138.4 (C), 130.30 (2 CH), 130.28 (2 CH), 129.8 (C), 129.6 (CH), 127.7 (2 CH), 127.5 (2 CH), 79.3 (C, HSQC), 72.2 (CH), 71.7 (C), 59.7 (CH_2), 41.2 (CH_2), 15.4 (CH_2), 14.1 (CH_3); **FTIR (neat, cm^{-1}):** 3301, 3244, 3061, 2978, 1650, 1586, 1554, 1478, 1392, 1367, 1329, 1283, 1093, 1063, 1010; **HRMS (ESI+)**: m/z: $[M+Na]^+$ Calcd. for $C_{21}H_{19}O_3ClSNa^+$ 409.0636; Found 409.0638.

Ethyl 2-((2-bromophenyl)(but-3-yn-1-yl)-l4-sulfaneylidene)-3-oxo-3-phenylpropanoate (1k)

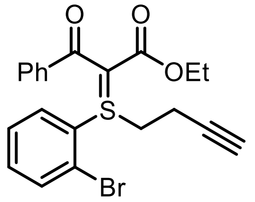

Synthesized according to **GP3** from sulfoxide **4d** (8.36 mmol, 2.15 g) and the corresponding commercially available β-keto ester. 88%, yellow highly viscous oil.

^{1}H NMR (600 MHz, CDCl$_3$) δ 8.02 (dd, J = 8.0, 1.5 Hz, 1H), 7.68 (dd, J = 8.0, 1.3 Hz, 1H), 7.53–7.52 (m, 2H), 7.48 (td, J = 7.8, 1.3 Hz, 1H), 7.41–7.32 (m, 4H), 4.69 (ddd, J = 12.2, 6.6, 5.8 Hz, 1H), 3.96 (q, J = 7.1 Hz, 2H), 3.68 (dt, J = 11.8, 7.9 Hz, 1H), 2.74–2.71 (m, 2H), 2.17–2.16 (m, 1H), 0.94 (t, J = 7.1 Hz, 3H); **^{13}C NMR (151 MHz, CDCl$_3$)** δ 191.1 (C), 166.9 (C), 143.2 (C), 134.2 (CH), 133.3 (CH), 131.9 (CH), 131.4 (C), 129.7 (CH), 129.0 (CH), 127.7 (2 CH), 127.6 (2 CH), 123.04 (C), 79.0 (C, HSQC), 71.9 (CH), 69.4 (C), 59.8 (CH_2), 39.7 (CH_2), 15.6 (CH_2), 14.1 (CH_3); **FTIR (neat, cm^{-1}):** 3297, 3243, 3060, 2979, 2902, 1652, 1553, 1445, 1366, 1327, 1278, 1164, 1129, 1060, 1018; **HRMS (ESI+)**: m/z: $[M+Na]^+$ Calcd. for $C_{21}H_{19}BrO_3SNa^+$ 453.0130; Found 453.0137.

Ethyl 2-(but-3-yn-1-yl(o-tolyl)-l4-sulfaneylidene)-3-oxo-3-phenylpropanoate (1l)

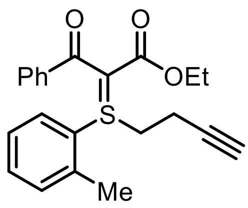

Synthesized according to **GP3** from sulfoxide **4e** (7.45 mmol, 1.43 g) and the corresponding commercially available β-keto ester. 97% yellow highly viscous oil.

^{1}H NMR (600 MHz, CDCl$_3$) δ 8.21 (d, J = 8.0 Hz, 1H), 7.45–7.40 (m, 3H), 7.38–7.27 (m, 5H), 4.55–4.49 (m, 1H), 3.93 (q, J = 7.1 Hz, 2H), 3.68 (dt, J = 12.1, 7.4 Hz, 1H), 2.74–2.62 (m, 5H), 2.15 (t, J = 2.6 Hz, 1H), 0.90 (t, J = 7.1 Hz, 3H); **^{13}C NMR (151 MHz, CDCl$_3$)** δ 191.0 (C), 167.1 (C), 143.6 (C), 139.5 (C), 132.1 (CH), 131.7 (CH), 130.5 (CH), 129.7 (C), 129.3 (CH), 127.7 (CH), 127.49 (2 CH), 127.45 (2 CH), 79.6 (C, HSQC), 72.7 (C), 71.8 (CH), 59.5 (CH$_2$), 39.6 (CH$_2$), 20.1 (CH$_3$), 15.5 (CH$_2$), 14.1 (CH$_3$); **FTIR (neat, cm^{-1}):** 3297, 3243, 3060, 2979, 2902, 1652, 1553, 1445, 1366, 1327, 1278, 1164, 1129, 1060, 1018; **HRMS (ESI+)**: m/z: [M+Na]$^+$ Calcd. for $C_{22}H_{22}O_3SNa^+$ 389.1182; Found 389.1185.

6. Synthesis of dihydrobenzothiepines 2a–l

Condition screening

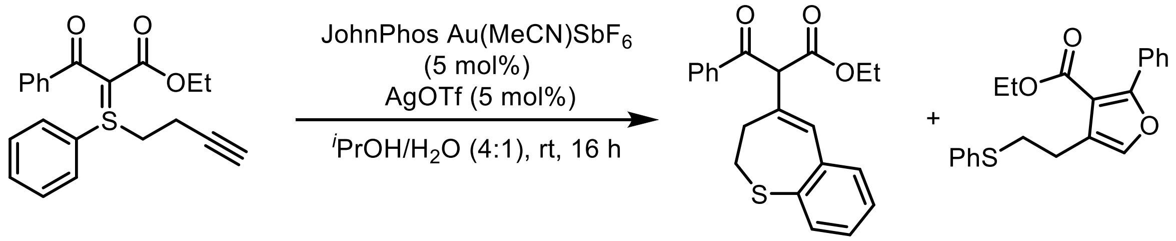

1a (0.1 mmol) **2a** **2a''**

Entry	Solvent	T/°C	Additive	Y(**2a**)[a]/%	Y(**2a''**)[a]/%
**1**	MeOH	25	—	42	5
**2**	MeOH	25	TfOH	34	6
**3**	MeOH	25	AgOTf	49	8
**4**	DCE	25	AgOTf	42	12
**5**	1,4-dioxane	25	AgOTf	49	32
**6**	Toluene	25	AgOTf	37	20
**7**	MeCN	25	AgOTf	45	5
**8**	^{i}PrOH/H$_2$O (4:1 v/v)	25	AgOTf	65	<5
**9**	^{i}PrOH/H$_2$O (4:1 v/v)	50	AgOTf	52	12

[a] Yields were determined by ^{1}H-NMR analysis with bromomesitylene as internal standard.

General procedure for the Au(I)-catalyzed dihydrobenzothiepine synthesis (GP4)

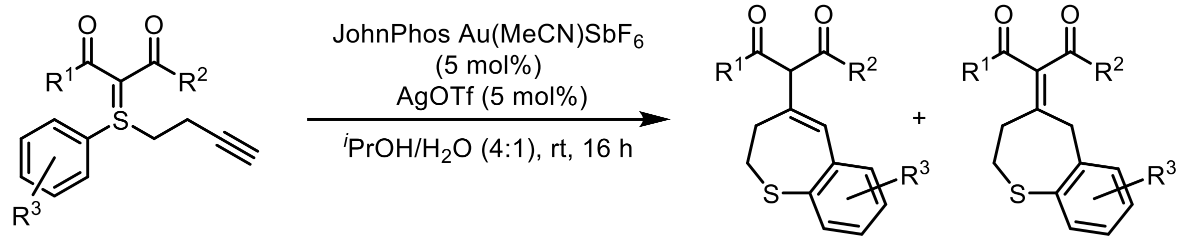

1a–l **2a–l** (exclusive or major) **2a'–l'** (not observed or minor)

To a solution of sulfonium ylide (0.2 mmol, 1 equiv.) in ^{i}PrOH/H_2O (4:1, 3 ml mmol^{-1}) was added a solution of (JohnPhos)AuSbF_6(MeCN) (0.05 equiv., 0.01 mmol) in ^{i}PrOH/H_2O (4:1, 6 ml mmol^{-1}) and a solution of AgOTf (0.05 equiv., 0.01 mmol) in ^{i}PrOH/H_2O (4:1, 1 ml mmol^{-1}). The mixture was stirred for 16 h at room temperature. Silica gel was added, and volatiles were evaporated under reduced pressure. The residue was directly loaded onto a flash column and the crude purified by flash chromatography (silica gel, EtOAc(0⟶2%)/heptane, unless otherwise stated).

Ethyl 2-(2,3-dihydrobenzo[b]thiepin-4-yl)-3-oxo-3-phenylpropanoate (2a) & Ethyl 2-(2,3-dihydrobenzo[b]thiepin-4(5H)-ylidene)-3-oxo-3-phenylpropanoate (2a')

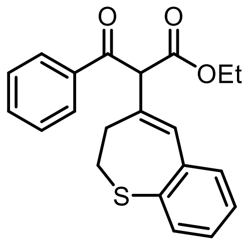

2a

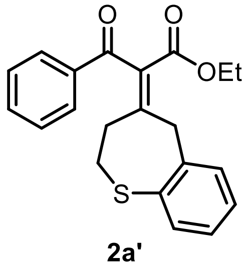

2a'

Synthesized according to **GP4** from sulfonium ylide **1a**. Obtained as a mixture of regioisomers (**2a/2a'** = 83:17). **2a** is present in $CDCl_3$ as a mixture of tautomers (***keto/enol*** = 95:5). 77%, yellow oil.

^{1}H NMR (600 MHz, $CDCl_3$) δ 13.38 (s, 1H (***enol*-2a**)), 8.08–8.01 (m, 2H (***keto*-2a**)), 7.84 (dd, *J* = 8.3, 1.2 Hz, 2H (**2a'**)), 7.73 (dd, *J* = 7.9, 1.7 Hz, 2H (***enol*-2a**)), 7.58 (dtd, *J* = 4.2, 2.7, 1.3 Hz, 1H (***keto*-2a**)), 7.54 (d, *J* = 7.4 Hz, 1H (**2a'**)), 7.50–7.45 (m, 2H (***keto*-2a**)), 7.44–7.39 (m, 1H (***keto*-2a**), 2H (**2a'**)), 7.23–7.18 (m, 2H (***keto*-2a**)), 7.12–7.07 (m, 1H (***keto*-2a**)), 7.05 (dd, *J* = 7.6, 1.4 Hz, 1H (**2a'**)), 6.95 (td, *J* = 7.5, 1.3 Hz, 1H (**2a'**)), 6.88 (d, *J* = 6.7 Hz, 1H (***enol*-2a**)), 6.78 (dd, *J* = 7.5, 0.8 Hz, 1H (**2a'**)), 6.66 (s, 1H (***keto*-2a**)), 6.36 (s, 1H (***enol*-2a**)), 5.23 (s, 1H (***keto*-2a**)), 4.32 (q, *J* = 7.1 Hz, 2H (***enol*-2a**)), 4.29–4.23 (m, 2H (***keto*-2a**)), 4.04 (q, *J* = 7.1 Hz, 2H (**2a'**)), 3.78 (s, 2H (**2a'**)), 3.34–3.32 (m, 2H (**2a'**)), 3.24 (ddd, *J* = 13.3, 7.7, 4.3 Hz, 1H (***keto*-2a**)), 3.15 (m, 2H (***enol*-2a**)), 3.15–3.10 (m, 1H (***keto*-2a**)), 3.09–3.07 (m, 2H (**2a'**)), 2.90–2.85 (m, 1H (***keto*-2a**)), 2.75 (s, 2H (***enol*-2a**)), 2.72–2.67 (m, 1H (***keto*-2a**)), 1.34 (t, *J* = 7.1 Hz, 3H (***enol*-2a**)), 1.27 (t, *J* = 7.1 Hz, 3H (***keto*-2a**)), 0.98 (t, *J* = 7.1 Hz, 3H (**2a'**)); **^{13}C NMR (151 MHz, $CDCl_3$)** δ 195.1, 194.3, 173.4, 170.4, 169.8, 169.0, 164.6, 156.0, 140.9, 139.0, 138.7, 137.6, 137.4, 137.1, 136.64, 136.57, 136.0, 135.5, 135.1, 135.0, 133.9, 133.7, 133.6, 133.4, 132.52, 132.50, 132.4, 132.1, 130.8, 130.3, 130.1, 129.1, 128.9, 128.82, 128.79, 128.6, 128.2, 128.1, 127.3, 127.1, 126.9, 126.7, 64.2, 61.8, 61.3, 61.0, 42.0, 38.3, 36.8, 35.9, 35.8, 34.7, 33.0, 14.4, 14.2, 13.9; **FTIR (neat, cm^{-1}):** 3057, 2979, 2924, 1732, 1678, 1595, 1446, 1367, 1290, 1256, 1175, 1156, 1025, 1004; **HRMS (ESI+)**: m/z: [M+Na]$^+$ Calcd. for $C_{21}H_{20}O_3SNa^+$ 375.1025; Found 375.1028.

Ethyl 2-(2,3-dihydrobenzo[b]thiepin-4-yl)-3-(4-methoxyphenyl)-3-oxopropanoate (2b)

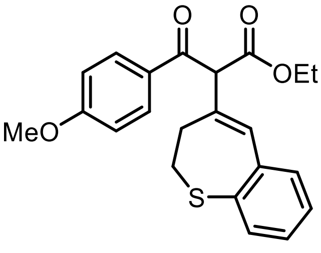

Synthesized according to **GP4** from sulfonium ylide **1b**. Column chromatography with EtOAc(0⟶8%)/heptane. **2b** is present in $CDCl_3$ as a mixture of tautomers (***keto/enol*** = 93:7). 58%, brown oil.

^{1}H NMR (600 MHz, $CDCl_3$) δ 13.48 (s, 1H (***enol*-2b**)), 8.04–8.02 (m, 2H (***keto*-2b**)), 7.80 (d, J = 9.0 Hz, 2H (***enol*-2b**)), 7.42 (d, J = 7.6 Hz, 1H (***keto*-2b**)), 7.22–7.19 (m, 2H (***keto*-2b**)), 7.16–7.13 (m, 1H (***enol*-2b**)), 7.11–7.08 (m, 1H (***keto*-2b**)), 7.08–7.06 (m, 1H (***enol*-2b**)), 6.99 (d, J = 8.7 Hz, 1H (***enol*-2b**)), 6.95–6.93 (m, 2H (***keto*-2b**)), 6.83 (d, J = 9.0 Hz, 2H (***enol*-2b**)), 6.65 (s, 1H (***keto*-2b**)), 6.42 (s, 1H (***enol*-2b**)), 5.18 (s, 1H (***keto*-2b**)), 4.30 (dd, J = 14.3, 7.1 Hz, 2H (***enol*-2b**)), 4.28–4.22 (m, 2H (***keto*-2b**)), 3.86 (s, 3H (***keto*-2b**)), 3.79 (s, 3H (***enol*-2b**)), 3.24 (ddd, J = 13.1, 7.7, 4.3 Hz, 1H (***keto*-2b**)), 3.15 (t, J = 6.0 Hz, 2H (***enol*-2b**)), 3.13–3.09 (m, 1H (***keto*-2b**)), 2.86 (ddd, J = 17.1, 8.1, 4.2 Hz, 1H (***keto*-2b**)), 2.78 (s, 2H (***enol*-2b**)), 2.67 (ddd, J = 17.1, 7.6, 4.2 Hz, 1H (***keto*-2b**)), 1.32 (t, J = 7.1 Hz, 3H (***enol*-2b**)), 1.28 (t, J = 7.1 Hz, 3H (***keto*-2b**)); **^{13}C NMR (151 MHz, $CDCl_3$, only *keto*-2b assigned)** δ 192.8, 169.3, 164.2, 138.9, 137.9, 136.5, 133.3, 132.6, 132.5, 131.3, 129.0, 127.2, 127.1, 114.1, 64.1, 61.7, 55.7, 36.9, 34.5, 14.2; **FTIR (neat, cm^{-1}):** 3055, 2977, 2931, 2840, 1734, 1670, 1598, 1574, 1510, 1463, 1419, 1316, 1257, 1169, 1027; **HRMS (ESI+)**: m/z: $[M+Na]^+$ Calcd. for $C_{22}H_{22}O_4SNa^+$ 405.1131; Found 405.1132.

Ethyl 2-(2,3-dihydrobenzo[b]thiepin-4-yl)-3-(4-nitrophenyl)-3-oxopropanoate (2c) & Ethyl 2-(2,3-dihydrobenzo[b]thiepin-4(5H)-ylidene)-3-(4-nitrophenyl)-3-oxopropanoate (2c')

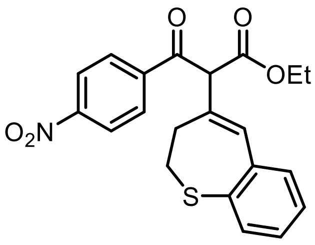

2c

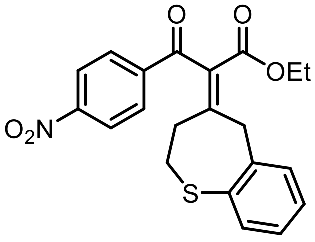

2c'

Synthesized according to **GP4** from sulfonium ylide **1c** at 50 °C. Column chromatography with EtOAc(0⟶8%)/heptane. Obtained as a mixture of regioisomers (**2c/2c'** = 92:8). **2c** is present in $CDCl_3$ as a mixture of tautomers (***keto/enol*** = 74:26). 64%, yellow oil.

^{1}H NMR (600 MHz, $CDCl_3$) δ 13.35 (s, 1H (***enol*-2c**)), 8.32 (d, J = 8.9 Hz, 2H (***keto*-2c**)), 8.23 (d, J = 8.9 Hz, 2H (**2c'**)), 8.20 (d, J = 8.9 Hz, 2H (***keto*-2c**)), 8.17 (d, J = 9.0 Hz, 2H (***enol*-2c**)), 7.95 (d, J = 9.0 Hz, 2H (***enol*-2c**)), 7.92 (d, J = 8.9 Hz, 2H (**2c'**)), 7.49–7.47 (m, 1H (**2c'**)), 7.45–7.42 (m, 1H (***keto*-2c**)), 7.42–7.39 (m, 1H (***enol*-2c**)), 7.24–7.18 (m, 2H (***keto*-2c**)), 7.14–7.11 (m, 1H (***keto*-2c**), 1H (***enol*-2c**)), 7.09–7.06 (m, 1H (***enol*-2c**), 1H (***2c'***)), 6.94 (dd, J = 7.5, 1.3 Hz, 1H (**2c'**)), 6.91 (d, J = 7.3 Hz, 1H (***enol*-2c**)), 6.76 (d, J = 7.6 Hz, 1H (**2c'**)), 6.65 (s, 1H (***keto*-2c**)), 6.34 (s, 1H (***enol*-2c**)), 5.17 (s, 1H (***keto*-2c**)), 4.34 (q, J = 7.1 Hz, 2H (***enol*-2c**)), 4.31–4.23 (m, 2H (***keto*-2c**)), 4.03 (q, J = 7.1 Hz, 2H (**2c'**)), 3.81 (s, 2H (**2c'**)), 3.37 (dd, J = 6.7, 5.1 Hz, 1H (**2c'**)), 3.26–3.22 (m, 1H (***keto*-2c**)), 3.19–3.10 (m, 1H (***keto*-2c**), 2H (***enol*-2c**), 1H (**2c'**)), 3.08 (t, J = 6.4 Hz, 1H (**2c'**)), 2.98 (t, J = 6.3 Hz, 1H (**2c'**)), 2.87–2.82 (m, 1H (***keto*-2c**), 2H (***enol*-2c**)), 2.69 (ddd, J = 17.2, 7.5, 4.3 Hz, 1H (***keto*-2c**)), 1.35 (t, J = 7.1 Hz, 3H (***enol*-2c**)), 1.29 (t, J = 7.1 Hz, 3H (***keto*-2c**)), 0.97 (t, J = 7.1 Hz, 3H (**2c'**)); **^{13}C NMR (151 MHz, $CDCl_3$, only 2c assigned)** δ 193.1, 173.1, 168.3, 167.3, 150.7, 148.4, 141.1, 140.5, 138.4, 138.3, 136.9, 136.8, 136.6, 136.4, 136.2, 134.4, 132.7, 132.63, 132.61, 132.3, 129.90, 129.86, 127.6, 127.3, 127.2, 127.1, 124.2, 123.3, 110.0, 64.7, 62.2, 61.8, 38.6, 36.7, 35.4, 35.0, 14.3, 14.2; **FTIR (neat, cm^{-1}):** 3109, 3053, 2981, 2925, 1734, 1689, 1638, 1603, 1582, 1523, 1470, 1345, 1317, 1292, 1252, 1176, 1131, 1010; **HRMS (ESI+)**: m/z: $[M+Na]^+$ Calcd. for $C_{21}H_{19}NO_5SNa^+$ 420.0876; Found 420.0881.

Ethyl 2-(2,3-dihydrobenzo[b]thiepin-4-yl)-3-oxobutanoate (2d)

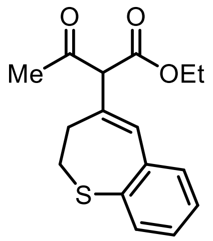

Synthesized according to **GP4** from sulfonium ylide **1d**. **2d** is present in $CDCl_3$ as a mixture of tautomers (***keto*/*enol*** = 50:50). 38%, brown oil.

^{1}H NMR (600 MHz, $CDCl_3$) δ 12.80 (d, J = 0.7 Hz, 1H (***enol*-2d**)), 7.47–7.45 (m, 1H (***keto*-2d**), 1H (***enol*-2d**)), 7.24–7.21 (m, 2H (***keto*-2d**), 1H (***enol*-2d**)), 7.19 (dd, J = 7.7, 1.6 Hz, 1H (***enol*-2d**)), 7.14–7.09 (m, 1H (***keto*-2d**), 1H (***enol*-2d**)), 6.61 (s, 1H (***keto*-2d**)), 6.41 (s, 1H (***enol*-2d**)), 4.32 (s, 1H (***keto*-2d**)), 4.25–4.21 (m, 2H (***keto*-2d**), 2H (***enol*-2d**)), 3.27–3.18 (m, 2H (***keto*-2d**), 2H (***enol*-2d**)), 2.79 (dddd, J = 17.1, 7.6, 4.5, 0.8 Hz, 1H (***keto*-2d**)), 2.69–2.63 (m, 1H (***keto*-2d**), 2H (***enol*-2d**)), 2.35 (s, 3H (***keto*-2d**)), 2.16 (s, 3H (***enol*-2d**)), 1.30 (t, J = 7.1 Hz, 3H (***keto*-2d**)), 1.27 (t, J = 7.1 Hz, 3H (***enol*-2d**)); **^{13}C NMR (151 MHz, $CDCl_3$)** δ 202.4, 173.5, 172.3, 168.6, 139.6, 139.1, 138.7, 136.60, 136.55, 136.5, 133.6, 133.2, 132.6, 132.5, 132.4, 127.4, 127.3, 127.2, 126.9, 107.3, 69.0, 61.7, 60.7, 37.4, 36.9, 36.8, 34.7, 29.1, 19.9, 14.4, 14.3; **FTIR (neat, cm^{-1}):** 3055, 2980, 2923, 1715, 1637, 1606, 1467, 1328, 1242, 1222, 1183, 1062, 1017; **HRMS (ESI+)**: m/z: $[M+Na]^+$ Calcd. for $C_{16}H_{18}NaO_3S^+$ 313.0869; Found 313.0869.

Diethyl 2-(2,3-dihydrobenzo[b]thiepin-4-yl)-3-oxoheptanedioate (2e)

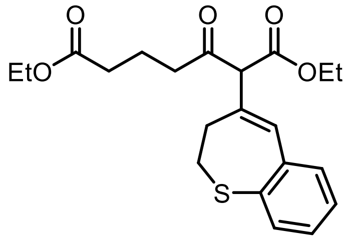

Synthesized according to **GP4** from sulfonium ylide **1e** (0.2 mmol, 78.1 mg). Column chromatography with EtOAc(0⟶8%)/heptane. **2e** is present in $CDCl_3$ as a mixture of tautomers (***keto*/*enol*** = 64:36). 67%, yellow viscous oil.

^{1}H NMR (600 MHz, $CDCl_3$) δ 12.84 (s, 1H (***enol*-2e**)), 7.46–7.42 (m, 1H (***keto*-2e**), 1H (***enol*-2e**)), 7.24–7.15 (m, 2H (***keto*-2e**), 2H (***enol*-2e**)), 7.13–7.07 (m, 1H (***keto*-2e**), 1H (***enol*-2e**)), 6.58 (s, 1H (***keto*-2e**)), 6.40 (s, 1H (***enol*-2e**)), 4.33 (s, 1H (***keto*-2e**)), 4.27–4.17 (m, 2H (***keto*-2e**), 2H (***enol*-2e**)), 4.09 (qd, J = 7.2, 1.8 Hz, 2H (***keto*-2e**)), 4.04 (dd, J = 14.2, 7.1 Hz, 2H (**enol-2e**)), 3.27–3.14 (m, 2H (***keto*-2e**), 2H (***enol*-2e**)), 2.83–2.59 (m, 3H (***keto*-2e**), 3H (***enol*-2e**)), 2.56 (t, J = 7.4 Hz, 1H (***keto*-2e**), 1H (***enol*-2e**)), 2.36–2.31 (m, 2H (***keto*-2e**), 2H (***enol*-2e**)), 2.00–1.87 (m, 2H (***keto*-2e**), 2H (***enol*-2e**)), 1.30–1.24 (m, 3H (***keto*-2e**), 3H (***enol*-2e**)), 1.21 (t, J = 7.1 Hz, 3H (***keto*-2e**)), 1.18 (t, J = 7.1 Hz, 3H (***enol*-2e**)); **^{13}C NMR (151 MHz, $CDCl_3$)** δ 203.7, 175.1, 173.12, 173.08, 172.4, 168.5, 139.4, 138.7, 138.5, 136.5, 136.51, 136.48, 133.48, 133.1, 132.6, 132.47, 132.45, 132.4, 127.3, 127.2, 127.1, 126.9, 107.5, 68.1, 61.6, 60.8, 60.5, 60.4, 40.6, 37.7, 36.9, 36.6, 34.5, 33.9, 33.1, 32.0, 22.3, 18.9, 14.32, 14.29, 14.26, 14.2; **FTIR (neat, cm^{-1}):** 3054, 2978, 2930, 1728, 1636, 1602, 1372, 1291, 1241, 1220, 1178, 1155, 1093, 1025; **HRMS (ESI+)**: m/z: $[M+Na]^+$ Calcd. for $C_{21}H_{26}O_5SNa^+$ 413.1393; Found 413.1398.

Prop-2-yn-1-yl 2-(2,3-dihydrobenzo[b]thiepin-4-yl)-3-oxo-3-phenylpropanoate (2f) & Prop-2-yn-1-yl 2-(2,3-dihydrobenzo[b]thiepin-4(5H)-ylidene)-3-oxo-3-phenylpropanoate (2f')

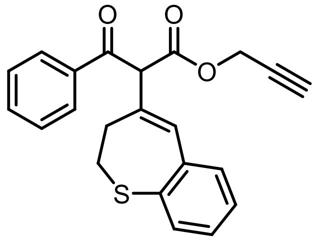

2f

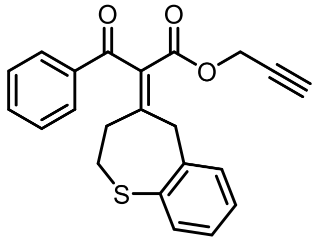

2f'

Synthesized according to **GP4** from sulfonium ylide **1f**. Obtained as a mixture of regioisomers (**2f/2f'** = 55:45). **2f** is present in $CDCl_3$ as a mixture of tautomers (***keto/enol*** = 85:15). 48%, yellow oil.

^{1}H NMR (600 MHz, $CDCl_3$) δ 13.06 (s, 1H (***enol*-2f**)), 8.04 (d, *J* = 7.3 Hz, 2H (***keto*-2f**)), 7.86 (dd, *J* = 8.2, 1.1 Hz, 2H (**2f'**)), 7.74 (dd, *J* = 8.1, 1.4 Hz, 2H (***enol*-2f**)), 7.61–7.55 (m, 1H (***keto*-2f**), 1H (***enol*-2f**), 1H (**2f'**)), 7.49–7.46 (m, 2H (***keto*-2f**), 1H (**2f'**)), 7.44–7.41 (m, 1H (***keto*-2f**), 1H (***enol*-2f**), 2H (**2f'**)), 7.35–7.33 (m, 2H (***enol*-2f**)), 7.21–7.19 (m, 2H (***keto*-2f**), 1H (***enol*-2f**)), 7.13–7.09 (m, 1H (***keto*-2f**), 1H (***enol*-2f**)), 7.07 (td, *J* = 7.5, 1.2 Hz, 1H (***enol*-2f**), 1H (**2f'**)), 6.96 (td, *J* = 7.5, 1.2 Hz, 1H (**2f'**)), 6.90 (d, *J* = 6.8 Hz, 1H (***enol*-2f**)), 6.77 (d, *J* = 7.5 Hz, 1H (**2f'**)), 6.68 (s, 1H (***keto*-2f**)), 6.38 (s, 1H (***enol*-2f**)), 5.28 (s, 1H (***keto*-2f**)), 4.83 (dd, *J* = 15.6, 2.5 Hz, 1H (***keto*-2f**), 1H (***enol*-2f**)), 4.75 (dd, *J* = 15.6, 2.5 Hz, 1H (***keto*-2f**), 1H (***enol*-2f**)), 4.57 (d, *J* = 2.4 Hz, 2H (**2f'**)), 3.78 (s, 2H (**2f'**)), 3.33 (dd, *J* = 7.2, 4.6 Hz, 2H (**2f'**)), 3.24 (ddd, *J* = 13.2, 7.7, 4.3 Hz, 1H (***keto*-2f**)), 3.17 (t, *J* = 6.0 Hz, 2H (***enol*-2f**)), 3.13–3.08 (m, 1H (***keto*-2f**), 2H (**2f'**)), 2.88 (ddd, *J* = 17.3, 8.0, 4.2 Hz, 1H (***keto*-2f**)), 2.77 (s, 2H (***enol*-2f**)), 2.68 (ddd, *J* = 17.2, 7.7, 4.2 Hz, 1H (***keto*-2f**)), 2.50 (t, *J* = 2.5 Hz, 1H (***keto*-2f**), 1H (***enol*-2f**)), 2.30 (t, *J* = 2.4 Hz, 1H (**2f'**)); **^{13}C NMR (151 MHz, $CDCl_3$)** δ 194.5, 193.8, 172.5, 171.4, 168.3, 163.8, 157.7, 140.6, 138.8, 138.6, 136.9, 136.8, 136.7, 136.0, 135.8, 135.1, 134.7, 134.1, 133.9, 133.5, 132.64, 132.55, 132.2, 130.4, 130.3, 129.8, 129.4, 129.0, 128.94, 128.93, 128.7, 128.3, 128.2, 127.42, 127.40, 127.2, 126.9, 126.8, 107.5, 75.6, 75.3, 75.0, 64.0, 53.1, 52.6, 52.3, 42.2, 38.4, 36.6, 36.0, 35.7, 34.8, 32.9, 29.9; **FTIR (neat, cm^{-1}):** 3285, 3057, 2923, 2130, 1746, 1678, 1595, 1447, 1369, 1266, 1215, 1150, 1026; **HRMS (ESI+)**: m/z: $[M+Na]^+$ Calcd. for $C_{22}H_{18}O_3SNa^+$ 385.0869; Found 385.0872.

Allyl 2-(2,3-dihydrobenzo[b]thiepin-4-yl)-3-oxobutanoate (2g)

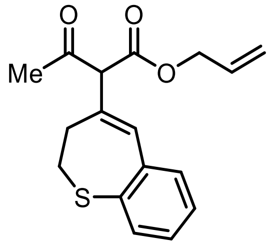

Synthesized according to **GP4** from sulfonium ylide **1g**. **2g** is present in $CDCl_3$ as a mixture of tautomers (***keto/enol*** = 45:55). 45%, yellow oil.

^{1}H NMR (600 MHz, $CDCl_3$) δ 12.69 (s, 1H (***enol*-2g**)), 7.46 (d, *J* = 7.7 Hz, 1H (***keto*-2g**), 1H (***enol*-2g**)), 7.25–7.18 (m, 2H (***keto*-2g**), 2H (***enol*-2g**)), 7.14–7.09 (m, 1H (***keto*-2g**), 1H (***enol*-2g**)), 6.62 (s, 1H (***keto*-2g**)), 6.44 (s, 1H (***enol*-2g**)), 5.96–5.85 (m, 1H (***keto*-2g**), 1H (***enol*-2g**)), 5.37–5.20 (m, 2H (***keto*-2g**), 2H (***enol*-2g**)), 4.68–4.67 (m, 2H (***keto*-2g**), 2H (***enol*-2g**)), 4.37 (s, 1H (***keto*-2g**)), 3.27–3.17 (m, 2H (***keto*-2g**), 2H (***enol*-2g**)), 2.82–2.78 (m, 1H (***keto*-2g**)), 2.72 (s, 2H (***enol*-2g**)), 2.68–2.63 (m, 1H (***keto*-2g**)), 2.35 (s, 3H (***keto*-2g**)), 2.17 (s, 3H (***enol*-2g**)); **^{13}C NMR (151 MHz, $CDCl_3$)** δ 202.1, 173.9, 171.9, 168.3, 139.4, 138.8, 138.6, 136.62, 136.56, 136.4, 133.7, 133.3, 132.63, 132.55, 132.49, 132.45, 132.0, 131.7, 127.4, 127.3, 127.2, 126.9, 119.1, 118.0, 107.2, 68.9, 66.2, 65.1, 37.6, 36.8, 36.6, 34.7, 29.1, 19.9; **FTIR (neat, cm^{-1}):** 3054, 2922, 1716, 1637, 1604, 1429, 1386, 1358, 1326, 1240, 1219, 1181, 1144, 1058; **HRMS (ESI+)**: m/z: $[M+Na]^+$ Calcd. for $C_{17}H_{18}O_3SNa^+$ 325.0869; Found 325.0873.

3-(2,3-dihydrobenzo[b]thiepin-4-yl)-4-hydroxypent-3-en-2-one (2h)

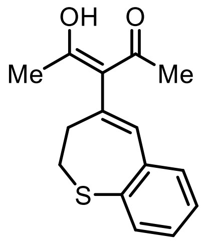

Synthesized according to **GP4** from sulfonium ylide **1h**. **2h** is present in $CDCl_3$ solely in its enol form. 54%, brown solid.

^{1}H NMR (600 MHz, $CDCl_3$) δ; 7.46 (d, J = 7.7 Hz, 1H), 7.23 (ddd, J = 15.3, 7.6, 3.7 Hz, 2H), 7.13 (td, J = 7.5, 1.6 Hz, 1H), 6.53 (s, 1H), 3.19–3.17 (m, 2H), 2.76–2.74 (m, 2H), 2.19 (s, 6H); **^{13}C NMR (151 MHz, $CDCl_3$)** δ 190.6 (2 C), 139.8, 138.5, 137.1, 134.6, 132.9, 132.4, 127.3, 127.2, 118.4, 39.8, 35.4, 23.6 (2 CH_3); **FTIR (neat, cm^{-1}):** 3054, 3007, 2921, 1587, 1469, 1409, 1307, 1284, 1220, 1161, 1132, 1075, 1020; **HRMS (ESI+)**: m/z: $[M+Na]^+$ Calcd. for $C_{15}H_{16}O_2SNa^+$ 283.0763; Found 283.0758.

Ethyl 2-(7-methoxy-2,3-dihydrobenzo[b]thiepin-4-yl)-3-oxo-3-phenylpropanoate (2i) & Ethyl 2-(7-methoxy-2,3-dihydrobenzo[b]thiepin-4(5H)-ylidene)-3-oxo-3-phenylpropanoate (2i')

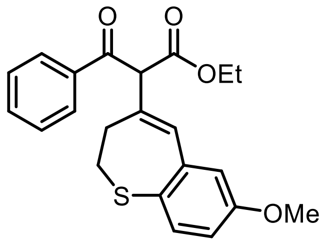

2i

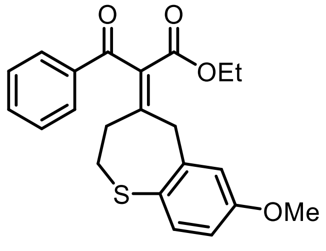

2i'

Synthesized according to **GP4** from sulfonium ylide **1i**. Column chromatography with EtOAc(0⟶9%)/heptane. Obtained as a mixture of regioisomers (**2i/2i'** = 81:19). **2i** is present in $CDCl_3$ as a mixture of tautomers (***keto*/*enol*** = 95:5). 69%, greenish oil.

^{1}H NMR (600 MHz, $CDCl_3$) δ 13.38 (s, 1H (***enol*-2i**)), 8.05 (dd, J = 8.3, 1.2 Hz, 2H (***keto*-2i**)), 7.92 (dd, J = 8.3, 1.2 Hz, 2H (***enol*-2i**)), 7.83 (dd, J = 6.6, 5.3 Hz, 1H (***enol*-2i**)), 7.81 (dd, J = 8.3, 1.2 Hz, 2H (**2i'**)), 7.70 (dd, J = 7.9, 1.7 Hz, 2H (***enol*-2i**)), 7.60–7.57 (m, 1H (***keto*-2i**)), 7.56–7.53 (m, 1H (**2i'**)), 7.48 (t, J = 7.8 Hz, 2H (***keto*-2i**)), 7.44 (d, J = 7.5 Hz, 1H (***enol*-2i**)), 7.42–7.40 (m, 1H (**2i'**)), 7.38 (d, J = 8.4 Hz, 1H (**2i'**)), 7.36 (d, J = 8.5 Hz, 1H (***keto*-2i**)), 7.34–7.32 (m, 1H (**2i'**)), 6.83 (d, J = 8.9 Hz, 1H (***enol*-2i**)), 6.73 (d, J = 2.8 Hz, 1H (***keto*-2i**)), 6.69 (dd, J = 8.5, 2.8 Hz, 1H (***keto*-2i**)), 6.64 (s, 1H (***keto*-2i**)), 6.59 (dd, J = 8.4, 2.8 Hz, 1H (**2i'**)), 6.34 (d, J = 2.8 Hz, 1H (***enol*-2i**)), 6.31 (d, J = 2.8 Hz, 1H (**2i'**)), 6.29 (s, 1H (***enol*-2i**)), 5.24 (s, 1H (***keto*-2i**)), 4.33 (q, J = 7.1 Hz, 2H (***enol*-2i**)), 4.25 (dtt, J = 10.8, 7.2, 3.7 Hz, 2H (***keto*-2i**)), 4.03 (q, J = 7.1 Hz, 2H (**2i'**)), 3.79 (s, 3H (**2i'**)), 3.77 (s, 3H (***keto*-2i**)), 3.71 (s, 3H (***enol*-2i**)), 3.44 (s, 2H (**2i'**)), 3.30–3.24 (m, 1H (***keto*-2i**), 2H (**2i'**)), 3.18 (t, J = 6.2 Hz, 2H (***enol*-2i**)), 3.11 (ddd, J = 13.0, 8.2, 4.8 Hz, 1H (***keto*-2i**)), 3.05–3.03 (m, 2H (**2i'**)), 2.75 (ddd, J = 16.2, 8.1, 4.8 Hz, 1H (***keto*-2i**)), 2.66–2.61 (m, 1H (***keto*-2i**), 2H (***enol*-2i**)), 1.35 (t, J = 7.1 Hz, 3H (***enol*-2i**)), 1.27 (t, J = 7.1 Hz, 3H (***keto*-2i**)), 0.97 (t, J = 7.1 Hz, 3H (**2i'**)); **^{13}C NMR (151 MHz, $CDCl_3$)** δ 195.2, 194.5, 194.3, 173.4, 170.6, 169.8, 169.0, 164.6, 159.6, 159.2, 158.9, 156.5, 147.4, 142.7, 141.3, 141.1, 138.0, 137.2, 136.0, 135.4, 135.1, 134.9, 134.3, 133.9, 133.7, 133.5, 132.3, 130.9, 129.1, 128.93, 128.89, 128.8, 128.7, 128.1, 127.2, 127.0, 125.9, 116.8, 116.7, 115.4, 114.6, 113.5, 113.3, 112.8, 63.8, 61.8, 61.3, 61.0, 55.5, 55.4, 55.1, 42.3, 38.6, 37.5, 36.5, 33.9, 33.6, 33.1, 14.4, 14.2, 13.9; **FTIR (neat, cm^{-1}):** 3061, 2959, 2933, 2837, 1734, 1678, 1592, 1561, 1466, 1447, 1368, 1306, 1271, 1239, 1173, 1158, 1074, 1029; **HRMS (ESI+)**: m/z: $[M+Na]^+$ Calcd. for $C_{22}H_{22}NaO_4SNa^+$ 405.1131; Found 405.1129.

Ethyl 2-(7-chloro-2,3-dihydrobenzo[b]thiepin-4-yl)-3-oxo-3-phenylpropanoate (2j) & Ethyl 2-(7-chloro-2,3-dihydrobenzo[b]thiepin-4(5H)-ylidene)-3-oxo-3-phenylpropanoate (2j')

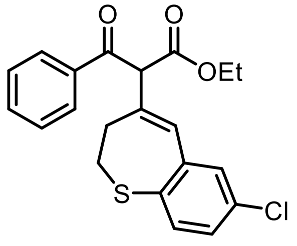

2j

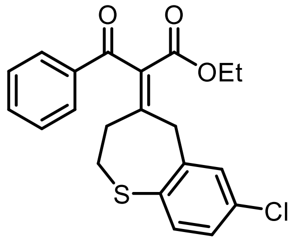

2j'

Synthesized according to **GP4** from sulfonium ylide **1j** (0.2 mmol, 77.4 mg). Column chromatography with EtOAc(0⟶4%)/heptane. Obtained as a mixture of regioisomers (**2j/2j'** = 86:14). **2j** is present in $CDCl_3$ as a mixture of tautomers (***keto/enol*** = 94:6). 41%, orange viscous oil.

^{1}H NMR (600 MHz, $CDCl_3$) δ 13.38 (s, 1H (***enol*-2j**)), 8.05–8.00 (m, 2H (***keto*-2j**)), 7.84–7.80 (m, 2H (**2j'**)), 7.71–7.68 (m, 2H (***enol*-2j**)), 7.62–7.54 (m, 1H (***keto*-2j**), 1H (***enol*-2j**), 1H (**2j'**)), 7.48 (t, *J* = 7.8 Hz, 2H (***keto*-2j**)), 7.45–7.40 (m, 2H (***enol*-2j**), 2H (**2j'**)), 7.38 (d, *J* = 8.2 Hz, 1H (**2j'**)), 7.35 (d, *J* = 8.3 Hz, 1H (***keto*-2j**)), 7.33–7.30 (m, 1H (***enol*-2j**)), 7.18 (d, *J* = 2.2 Hz, 1H (***keto*-2j**)), 7.07 (dd, *J* = 8.3, 2.2 Hz, 1H (***keto*-2j**)), 7.04–6.99 (m, 1H (***enol*-2j**),7.04–6.99 (m, 1H (**2j'**)), 6.85 (d, *J* = 2.2 Hz, 1H (***enol*-2j**)), 6.70 (d, *J* = 2.2 Hz, 1H (**2j'**)), 6.58 (s, 1H (***keto*-2j**)), 6.27 (s, 1H (***enol*-2j**)), 5.22 (s, 1H (***keto*-2j**)), 4.35–4.30 (m, 2H (***enol*-2j**)), 4.28–4.22 (m, 2H (***keto*-2j**)), 4.05 (q, *J* = 7.1 Hz, 2H (**2j'**)), 3.72 (s, 2H (**2j'**)), 3.35–3.30 (m, 2H (**2j'**)), 3.27–3.20 (m, 1H (***keto*-2j**)), 3.16–3.08 (m, 1H (***keto*-2j**),3.16–3.09 (m, 2H (***enol*-2j**)), 3.08–3.04 (m, 2H (**2j'**)), 2.84 (ddd, *J* = 17.0, 8.0, 4.4 Hz, 1H (***keto*-2j**)), 2.74 (br, 2H (***enol*-2j**)), 2.68 (ddd, *J* = 17.0, 7.5, 4.4 Hz, 1H (***keto*-2j**)), 1.34 (t, *J* = 7.1 Hz, 3H (***enol*-2j**)), 1.27 (t, *J* = 7.1 Hz, 3H (***keto*-2j**)), 0.99 (t, *J* = 7.1 Hz, 3H (**2j'**)); **^{13}C NMR (151 MHz, $CDCl_3$)** δ 194.9, 194.0, 173.2, 170.7, 168.8, 164.5, 154.7, 142.5, 140.7, 140.5, 139.4, 139.0, 136.9, 135.9, 135.3, 135.1, 135.0, 134.9, 134.5, 134.4, 134.3, 134.0, 133.9, 133.82, 133.76, 133.4, 133.1, 132.7, 132.4, 131.9, 131.7, 131.5, 130.3, 130.2, 129.1, 129.0, 128.9, 128.8, 128.6, 128.2, 127.2, 126.6, 107.7, 64.0, 61.9, 61.4, 61.1, 41.7, 38.1, 37.2, 36.02, 35.95, 34.4, 33.1, 14.4, 14.2, 13.9; **FTIR (neat, cm^{-1}):** 3060, 2979, 2926, 1738, 1681, 1638, 1596, 1579, 1466, 1448, 1368, 1290, 1263, 1178, 1159, 1106, 1026, 1004; **HRMS (ESI+)**: m/z: $[M+Na]^+$ Calcd. for $C_{21}H_{19}O_3ClSNa^+$ 409.0636; Found 409.0638.

Ethyl 2-(9-bromo-2,3-dihydrobenzo[b]thiepin-4-yl)-3-oxo-3-phenylpropanoate (2k) & Ethyl 2-(9-bromo-2,3-dihydrobenzo[b]thiepin-4(5H)-ylidene)-3-oxo-3-phenylpropanoate (2k')

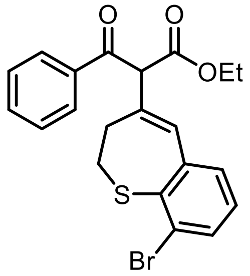

2k

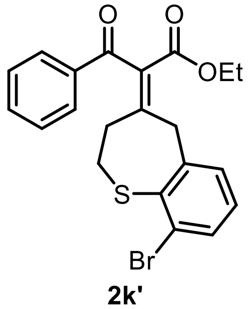

2k'

Synthesized according to **GP4** from sulfonium ylide **1k** (0.2 mmol, 86.3 mg). Column chromatography with EtOAc(0⟶4%)/heptane. Obtained as a mixture of regioisomers (**2k/2k'** = 90:10). **2k** is present in $CDCl_3$ as a mixture of tautomers (***keto/enol*** = 86:14). 64%, yellow viscous oil.

^{1}H NMR (600 MHz, $CDCl_3$) δ 13.37 (s, 1H (***enol*-2k**)), 8.04–7.98 (m, 2H (***keto*-2k**)), 7.86–7.83 (m, 2H (**2k'**)), 7.72–7.67 (m, 2H (***enol*-2k**)), 7.61–7.53 (m, 1H (***keto*-2k**), 1H (***enol*-2k**), 1H (**2k'**)), 7.51–7.40 (m, 3H (***keto*-2k**), 2H (***enol*-2k**)), 7.40–7.37 (m, 1H (***enol*-2k**, 1H (**2k'**)), 7.35–7.30 (m, 2H (**2k'**)), 7.15 (d, *J* = 7.2 Hz, 1H (***keto*-2k**)), 7.01 (t, *J* = 7.8 Hz, 1H (***keto*-2k**)), 6.92 (t, *J* = 7.8 Hz, 1H (***enol*-2k**)), 6.82 (d, *J* = 7.3 Hz, 1H (***enol*-2k**)), 6.78 (t, *J* = 7.7 Hz, 1H (**2k'**)), 6.74 (dd, *J* = 7.6, 1.3 Hz, 1H (**2k'**)), 6.63 (s, 1H (***keto*-2k**)), 6.33 (s, 1H (***enol*-2k**)), 5.19 (s, 1H (***keto*-2k**)), 4.29–4.20 (m, 2H (***keto*-2k**)), 4.31 (q, *J* = 7.1 Hz, 2H (***enol*-2k**)), 4.04 (q, *J* = 7.1 Hz, 2H (**2k'**)), 3.81 (s, 2H (**2k'**)), 3.36–3.31 (m, 2H (**2k'**)), 3.22 (ddd, *J* = 13.8, 7.6, 4.3 Hz, 1H (***keto*-2k**)), 3.17–3.09 (m, 1H (***keto*-2k**), 1H (***enol*-2k**), 1H (**2k'**)), 2.93–2.86 (m, 1H (***keto*-2k**)), 2.77 (br, 2H (***enol*-2k**)), 2.74–2.66 (m, 1H (***keto*-

2k)), 1.27 (t, *J* = 7.1 Hz, 3H (***keto*-2k**)), 1.33 (t, *J* = 7.1 Hz, 3H (***enol*-2k**)), 0.98 (t, *J* = 7.1 Hz, 3H (**2k'**)); **^{13}C NMR (151 MHz, $CDCl_3$)** *δ* 194.9, 194.1, 173.2, 170.5, 168.9, 164.5, 154.9, 142.5, 140.2, 139.9, 138.8, 138.31, 138.30, 138.2, 137.0, 136.6, 135.9, 135.3, 134.9, 133.9, 133.8, 133.2, 132.2, 131.93, 131.88, 131.4, 131.1, 130.9, 130.2, 129.6, 129.1, 129.0, 128.84, 128.77, 128.5, 128.1, 127.9, 127.4, 127.3, 127.0, 126.7, 107.8, 64.1, 61.9, 61.3, 61.1, 42.4, 38.0, 36.1, 35.5, 35.1, 34.9, 32.5, 29.8, 14.4, 14.2, 13.9; **FTIR (neat, cm^{-1}):** 3060, 2979, 2925, 1730, 1678, 1595, 1579, 1491, 1446, 1392, 1370, 1239, 1176, 1096, 1024; **HRMS (ESI+)**: m/z: $[M+Na]^+$ Calcd. for $C_{21}H_{19}O_3BrSNa^+$ 453.0130; Found 453.0121.

Ethyl 2-(9-methyl-2,3-dihydrobenzo[b]thiepin-4-yl)-3-oxo-3-phenylpropanoate (2l) & Ethyl 2-(9-methyl-2,3-dihydrobenzo[b]thiepin-4(5H)-ylidene)-3-oxo-3-phenylpropanoate (2l')

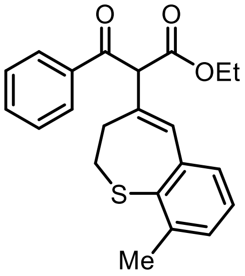

2l

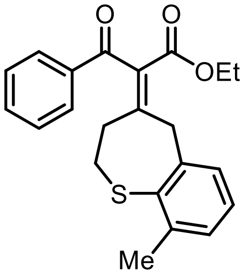

2l'

Synthesized according to **GP4** from sulfonium ylide **1l** (0.2 mmol, 73.3 mg). Column chromatography with EtOAc(0⟶3%)/heptane. Obtained as a mixture of regioisomers (**2l/2l'** = 97:3). **2l** is present in $CDCl_3$ as a mixture of tautomers (***keto/enol*** = 94:6). 75%, yellow viscous oil.

^{1}H NMR (600 MHz, $CDCl_3$) *δ* 13.38 (s, 1H (***enol*-2l**)), 8.05 (dd, *J* = 8.3, 1.1 Hz, 2H (***keto*-2l**)), 7.89–7.86 (m, 2H (**2l'**)), 7.75–7.72 (m, 2H (***enol*-2l**)), 7.61–7.55 (m, 1H (***keto*-2l**)), 7.50–7.45 (m, 2H (***keto*-2l**)), 7.45–7.40 (m, 1H (***enol*-2l**), 1H (**2l'**)), 7.35–7.30 (m, 2H (***enol*-2l**), 2H (**2l'**)), 7.12–7.04 (m, 3H (***keto*-2l**), 3H (**2l'**)), 7.04–6.98 (m, 3H (***enol*-2l**)), 6.68 (s, 1H (***keto*-2l**)), 6.37 (s, 1H (***enol*-2l**)), 5.22 (s, 1H (***keto*-2l**)), 4.32 (q, *J* = 7.1 Hz, 2H (***enol*-2l**)), 4.30–4.21 (m, 2H (***keto*-2l**)), 4.04 (q, *J* = 7.1 Hz, 2H (**2l'**)), 3.81 (s, 2H (**2l'**)), 3.30–3.26 (m, 2H (**2l'**)), 3.19 (ddd, *J* = 13.4, 7.6, 4.5 Hz, 1H (***keto*-2l**)), 3.14–3.03 (m, 1H (***keto*-2l**), 2H (***enol*-2l**), 2H (**2l'**)), 2.91–2.81 (m, 1H (***keto*-2l**)), 2.72 (br, 2H (***enol*-2l**)), 2.70–2.61 (m, 1H (***keto*-2l**)), 2.45 (s, 3H (**2l'**)), 2.43 (s, 3H (***enol*-2l**)), 2.42 (s, 3H (***keto*-2l**)), 1.34 (t, *J* = 7.1 Hz, 3H (***enol*-2l**)), 1.28 (t, *J* = 7.1 Hz, 3H (***keto*-2l**)), 0.99 (t, *J* = 7.1 Hz, 3H (**2l'**)); **^{13}C NMR (151 MHz, $CDCl_3$)** *δ* 195.2, 194.4, 173.5, 170.3, 169.1, 164.7, 156.4, 141.4, 140.8, 139.6, 139.4, 139.2, 139.1, 137.2, 137.1, 136.9, 136.7, 136.6, 136.1, 136.0, 135.1, 134.2, 134.0, 133.8, 133.6, 133.1, 130.6, 130.4, 130.1, 129.2, 128.90, 128.87, 128.84, 128.81, 128.75, 128.6, 128.2, 128.06, 128.05, 127.5, 126.4, 126.2, 108.1, 64.3, 61.7, 61.2, 60.9, 42.1, 38.0, 36.5, 35.7, 34.9, 34.7, 32.4, 22.1, 22.00, 21.97, 14.4, 14.2, 13.9; **FTIR (neat, cm^{-1}):** 3056, 2976, 2922, 1733, 1678, 1595, 1579, 1446, 1367, 1287, 1260, 1176, 1155, 1096, 1031, 1003; **HRMS (ESI+)**: m/z: $[M+Na]^+$ Calcd. for $C_{22}H_{22}O_3SNa^+$ 389.1182; Found 389.1184.

7. References

[1] K. Fujiwara, Y. Suzuki, N. Koseki, S. Murata, A. Murai, H. Kawai, T. Suzuki, *Tetrahedron Lett.* **2011**, *52*, 5589–5592.

[2] L. Capella, P. C. Montevecchi, D. Nanni, *J. Org. Chem.* **1994**, *59*, 3368–3374.

[3] V. G. Lisnyak, T. Lynch-Colameta, S. A. Snyder, *Angew. Chem. Int. Ed.* **2018**, *57*, 15162–15166.

[4] J. L. Howard, Y. Sagatov, D. L. Browne, *Tetrahedron* **2018**, *74*, 3118–3123.

[5] C. Mottet, O. Hamelin, G. Garavel, J.-P. Deprés, A. E. Greene, *J. Org. Chem.* **1999**, *64*, 1380–1382.

8. NMR spectra

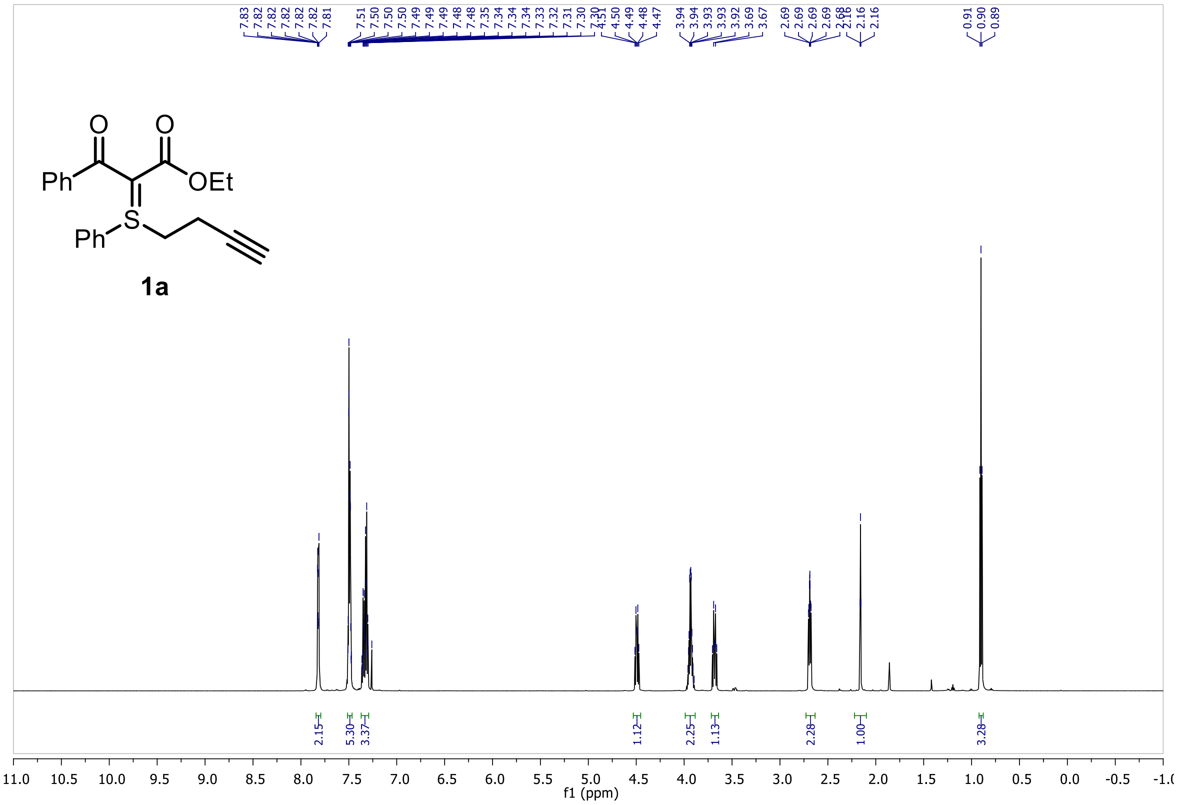

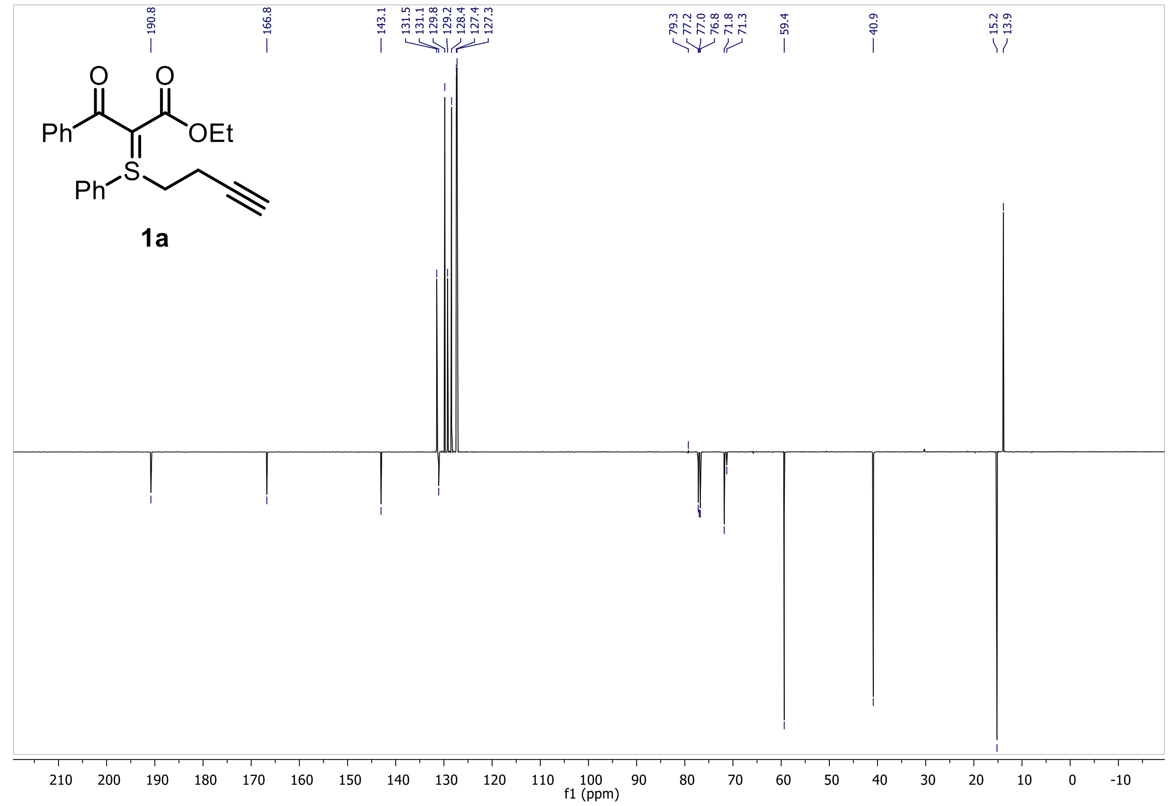

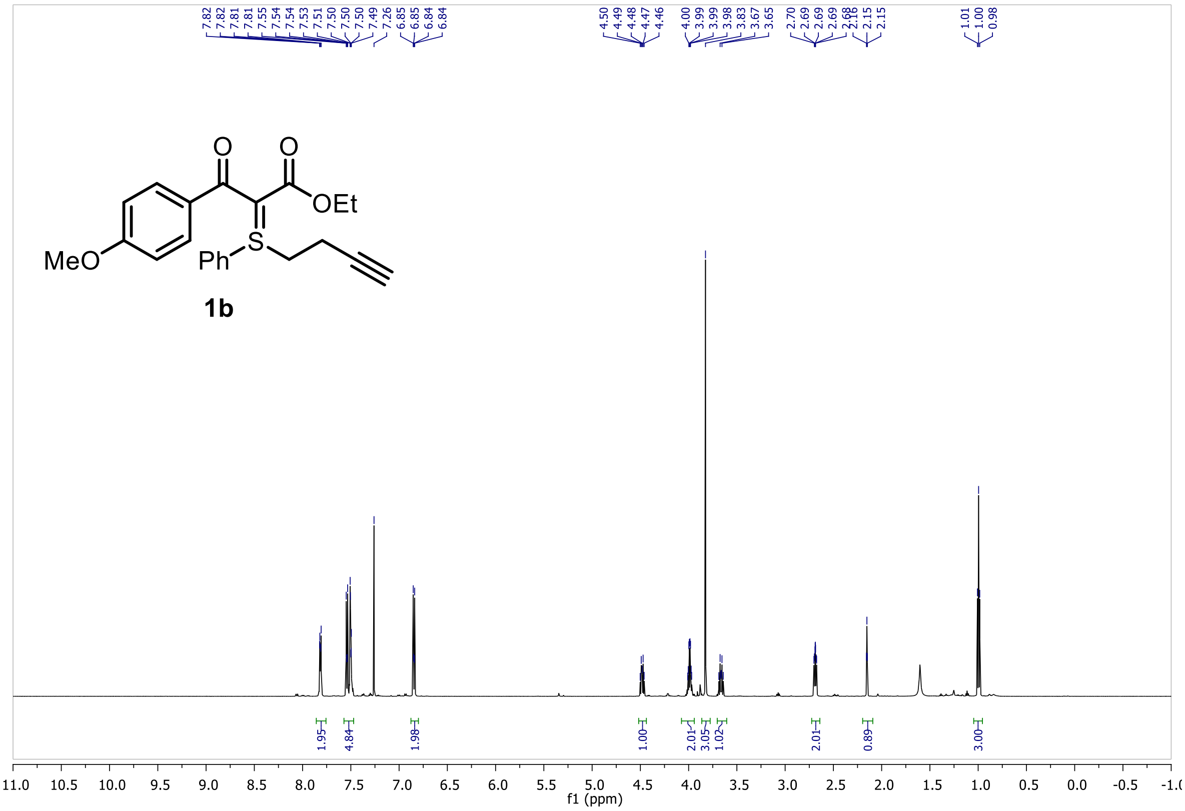
7.82
7.82
7.81
7.81
7.55
7.54
7.54
7.53
7.51
7.50
7.50
7.49
7.26
6.85
6.85
6.84
6.84
4.50
4.49
4.48
4.47
4.46
4.00
3.99
3.99
3.98
3.83
3.67
3.65
2.70
2.69
2.69
2.69
2.68
2.16
2.15
2.15
1.01
1.00
0.98
MeO
O
O
OEt
S
Ph
1b
1.95
4.84
1.98
1.00
2.01
3.05
1.02
2.01
0.89
3.00
f1 (ppm)

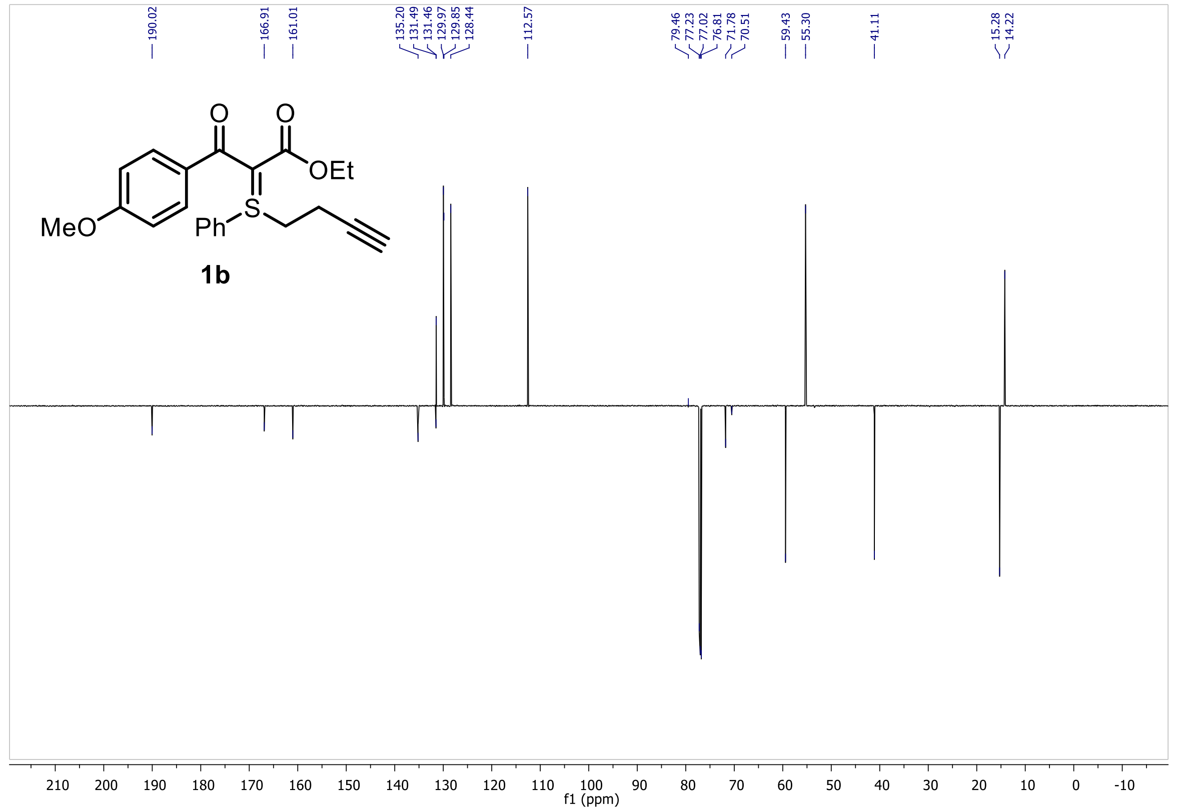
190.02
166.91
161.01
135.20
131.49
131.46
129.97
129.85
128.44
112.57
79.46
77.23
77.02
76.81
71.78
70.51
59.43
55.30
41.11
15.28
14.22
MeO
O
O
OEt
S
Ph
1b
f1 (ppm)

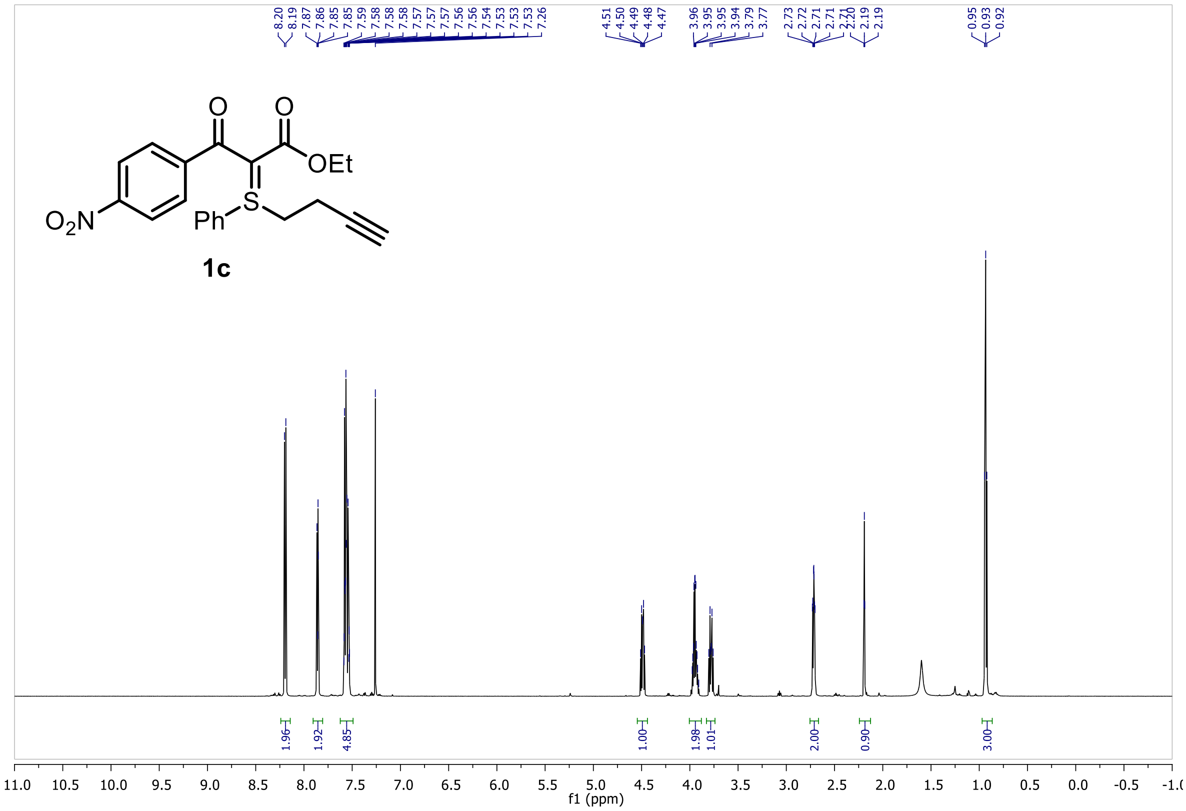
8.20
8.19
7.87
7.86
7.85
7.85
7.59
7.58
7.58
7.58
7.57
7.57
7.57
7.56
7.54
7.53
7.53
7.53
7.26
4.51
4.50
4.49
4.48
4.47
3.96
3.95
3.95
3.94
3.79
3.77
2.73
2.72
2.71
2.71
2.21
2.20
2.19
2.19
0.95
0.93
0.92
O
O
OEt
O_2N
Ph
S
1c
1.96
1.92
4.85
1.00
1.98
1.01
2.00
0.90
3.00
f1 (ppm)

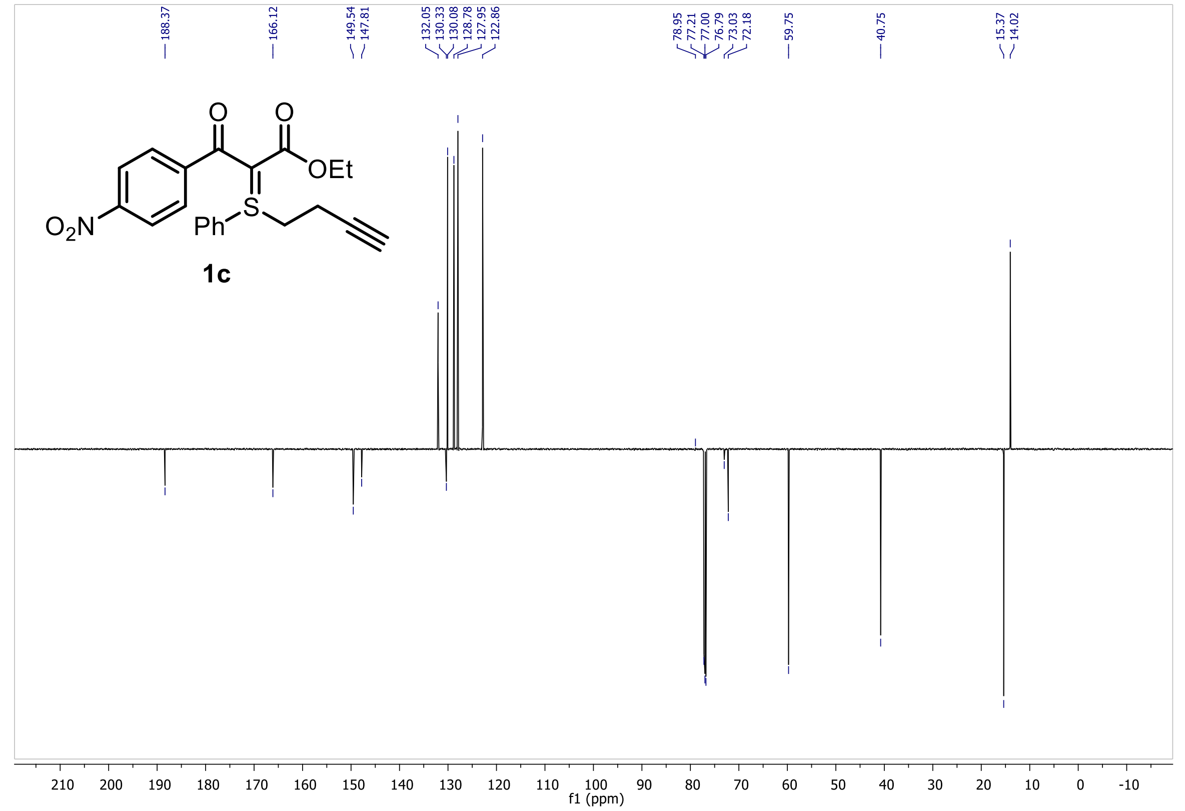
188.37
166.12
149.54
147.81
132.05
130.33
130.08
128.78
127.95
122.86
78.95
77.21
77.00
76.79
73.03
72.18
59.75
40.75
15.37
14.02
O
O
OEt
O_2N
Ph
S
1c
f1 (ppm)

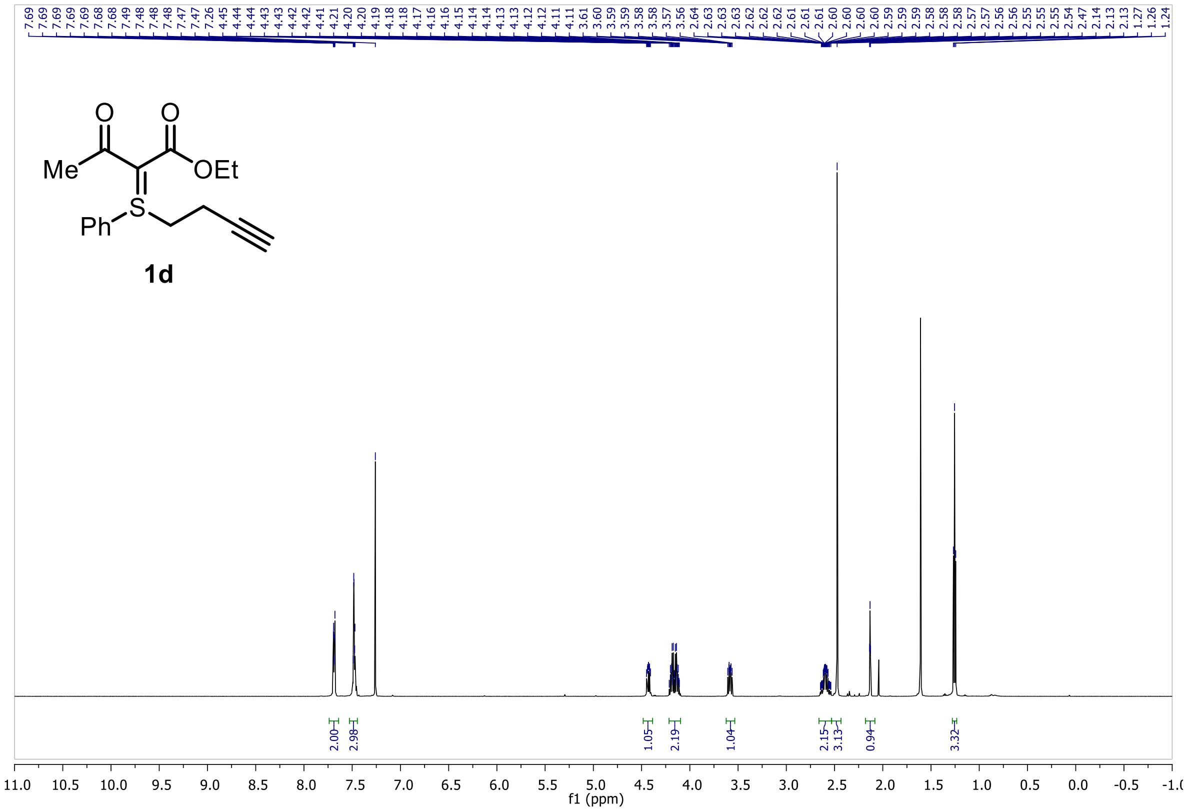

1d
f1 (ppm)

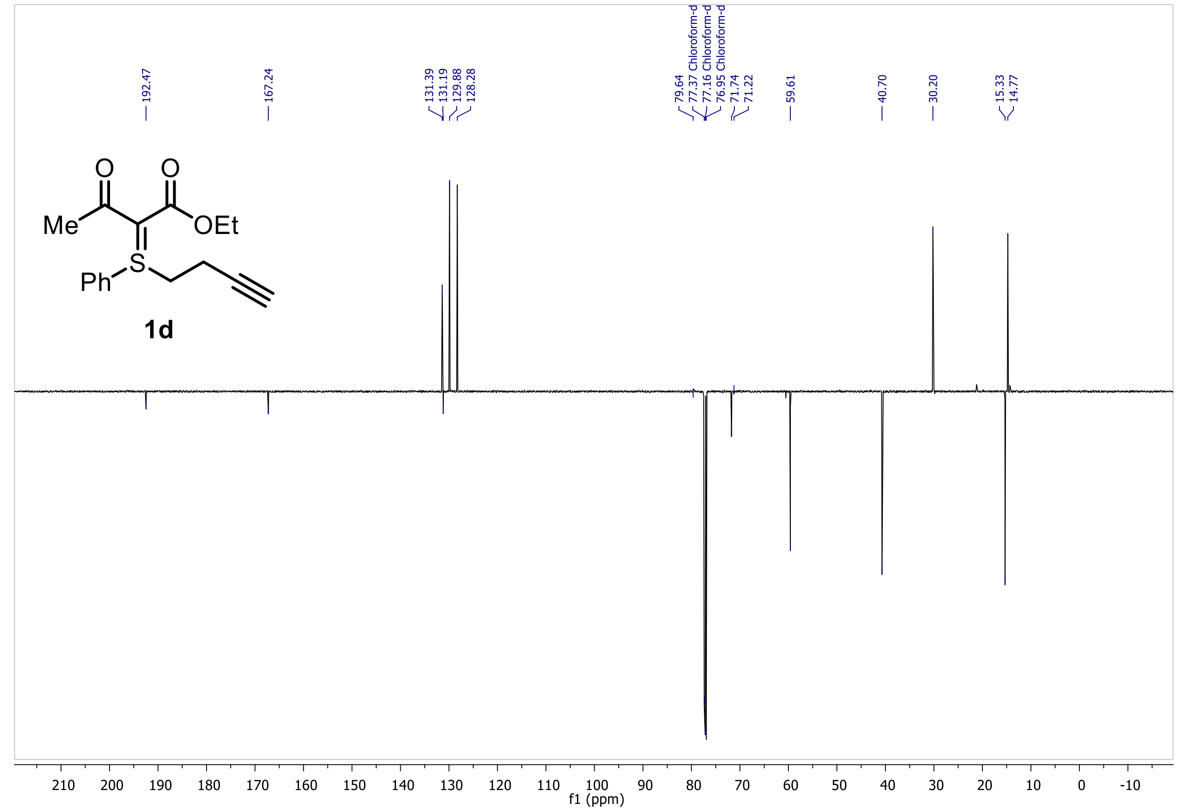

1d
f1 (ppm)

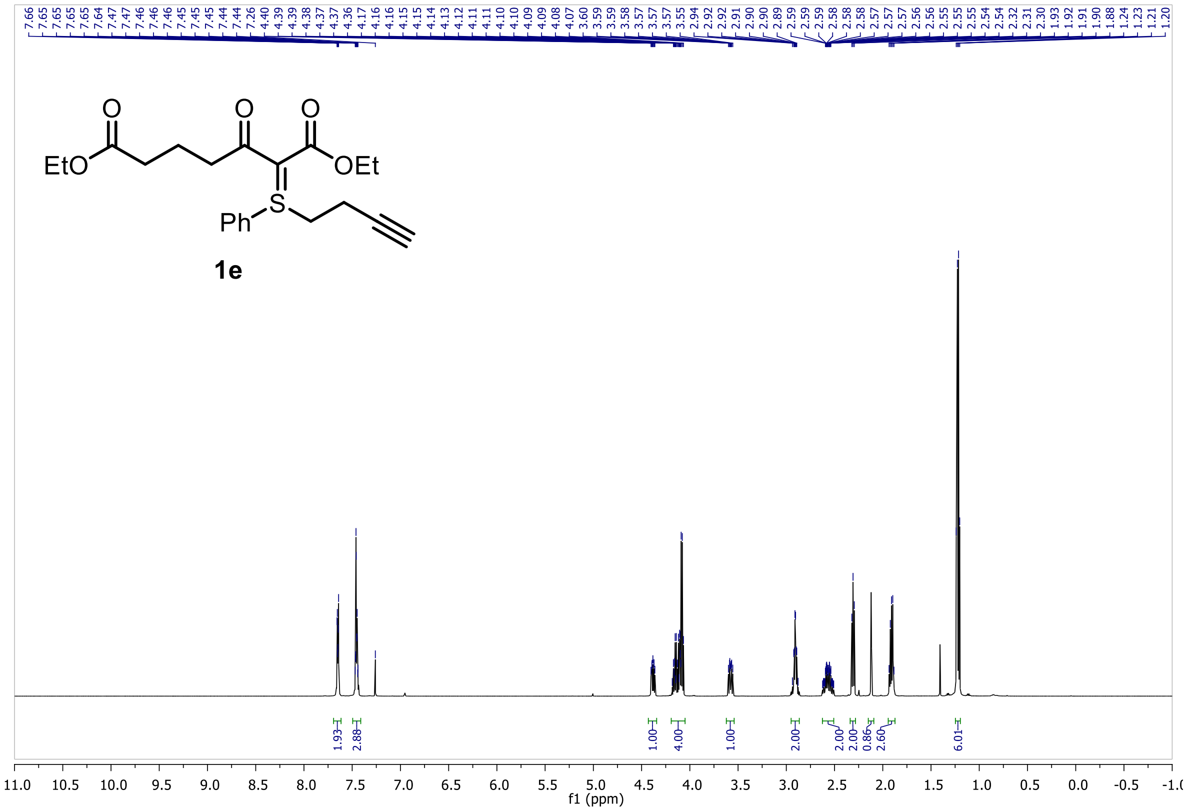
7.66 7.65 7.65 7.65 7.64 7.47 7.46 7.46 7.45 7.45 7.44 7.26 4.40 4.39 4.39 4.38 4.37 4.36 4.17 4.16 4.15 4.15 4.14 4.13 4.12 4.11 4.11 4.10 4.10 4.09 4.09 4.08 4.07 3.60 3.59 3.59 3.58 3.57 3.57 3.57 3.55 2.94 2.92 2.92 2.91 2.90 2.90 2.89 2.59 2.59 2.59 2.58 2.58 2.58 2.57 2.57 2.57 2.56 2.56 2.55 2.55 2.55 2.54 2.54 2.32 2.31 2.30 1.93 1.92 1.91 1.90 1.88 1.24 1.23 1.21 1.20
EtO
OEt
Ph
S
1e
1.93
2.88
1.00
4.00
1.00
2.00
2.00
2.00
0.86
2.60
6.01
f1 (ppm)

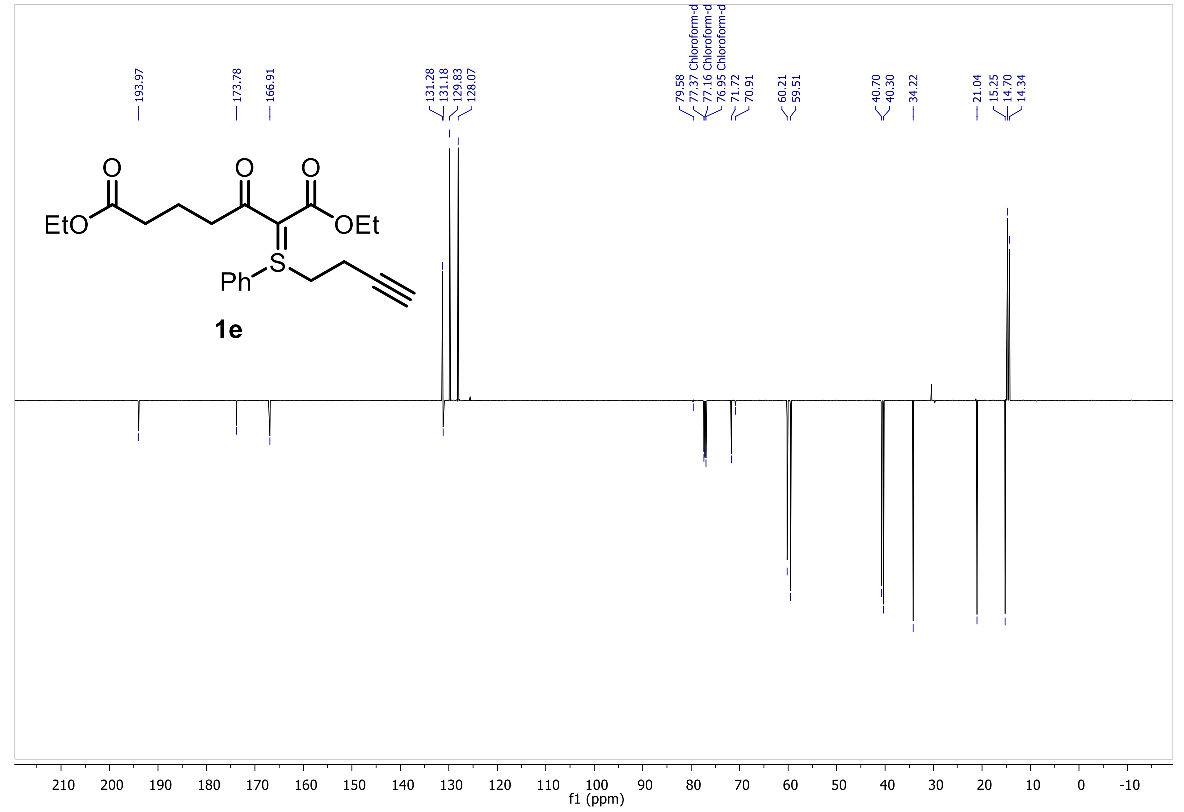
193.97
173.78
166.91
131.28 131.18 129.83 128.07
79.58 77.37 Chloroform-d 77.16 Chloroform-d 76.95 Chloroform-d 71.72 70.91
60.21 59.51
40.70 40.30
34.22
21.04 15.25 14.70 14.34
EtO
OEt
Ph
S
1e
f1 (ppm)

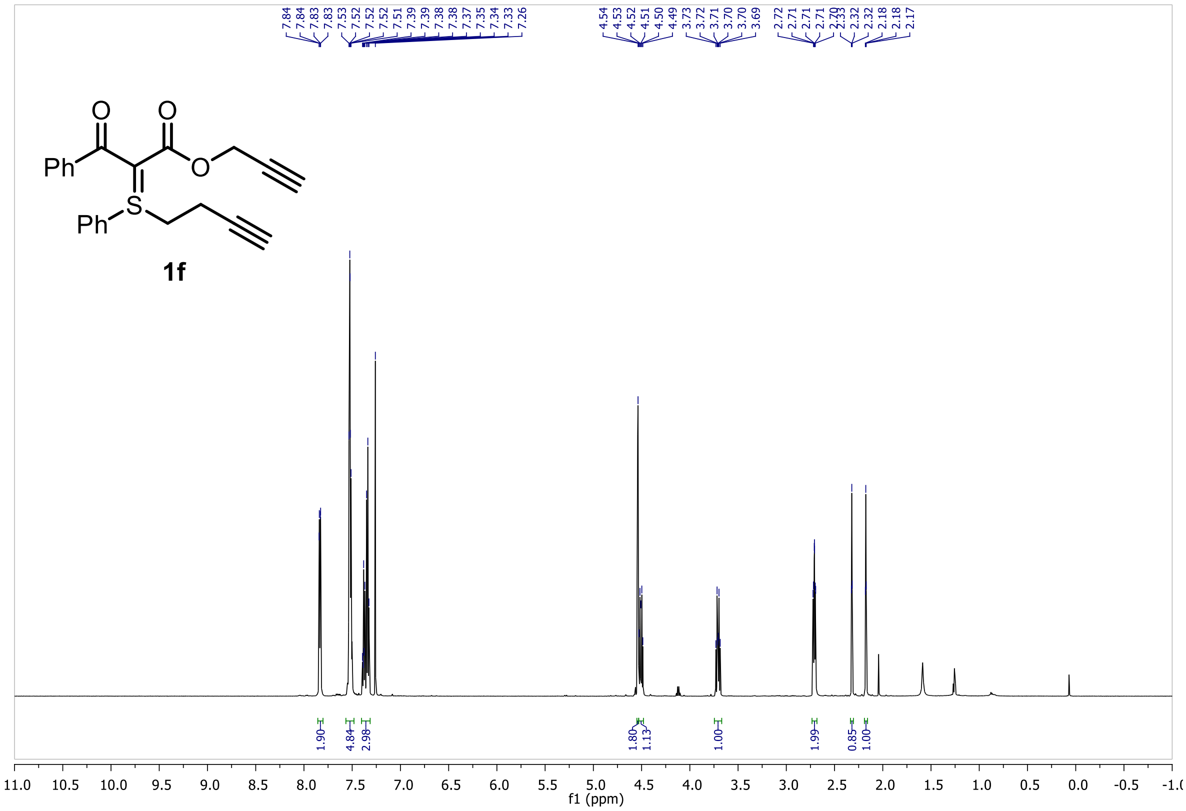
7.84 7.84 7.83 7.83 7.53 7.52 7.52 7.52 7.51 7.39 7.39 7.38 7.38 7.37 7.35 7.34 7.33 7.26
4.54 4.53 4.52 4.51 4.50 4.49 3.73 3.72 3.71 3.70 3.70 3.69 2.72 2.71 2.71 2.71 2.70 2.33 2.32 2.32 2.18 2.18 2.17
O
O
Ph
O
S
Ph
1f
1.90
4.84
2.98
1.80
1.13
1.00
1.99
0.85
1.00
11.0 10.5 10.0 9.5 9.0 8.5 8.0 7.5 7.0 6.5 6.0 5.5 5.0 4.5 4.0 3.5 3.0 2.5 2.0 1.5 1.0 0.5 0.0 -0.5
f1 (ppm)

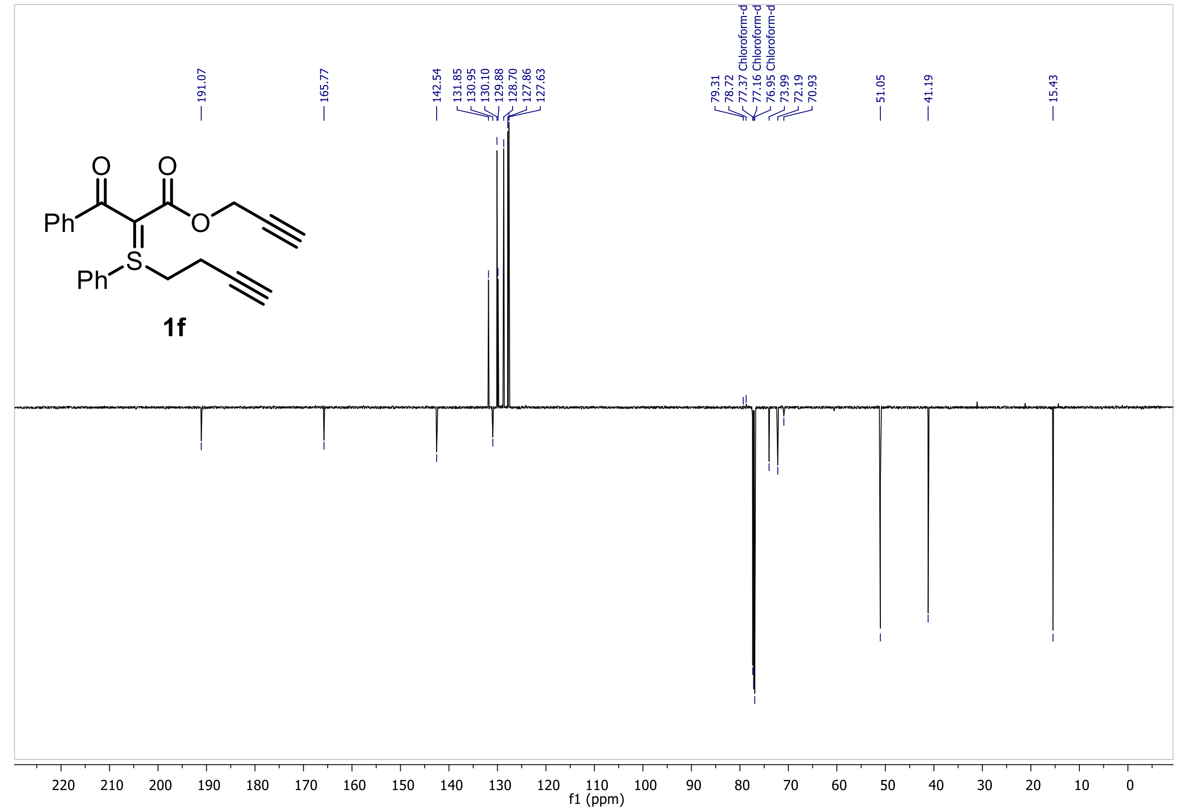
191.07
165.77
142.54 131.85 130.95 130.10 129.88 128.70 127.86 127.63
79.31 78.72 77.37 Chloroform-d 77.16 Chloroform-d 76.95 Chloroform-d 73.99 72.19 70.93
51.05
41.19
15.43
O
O
Ph
O
S
Ph
1f
220 210 200 190 180 170 160 150 140 130 120 110 100 90 80 70 60 50 40 30 20 10 0
f1 (ppm)

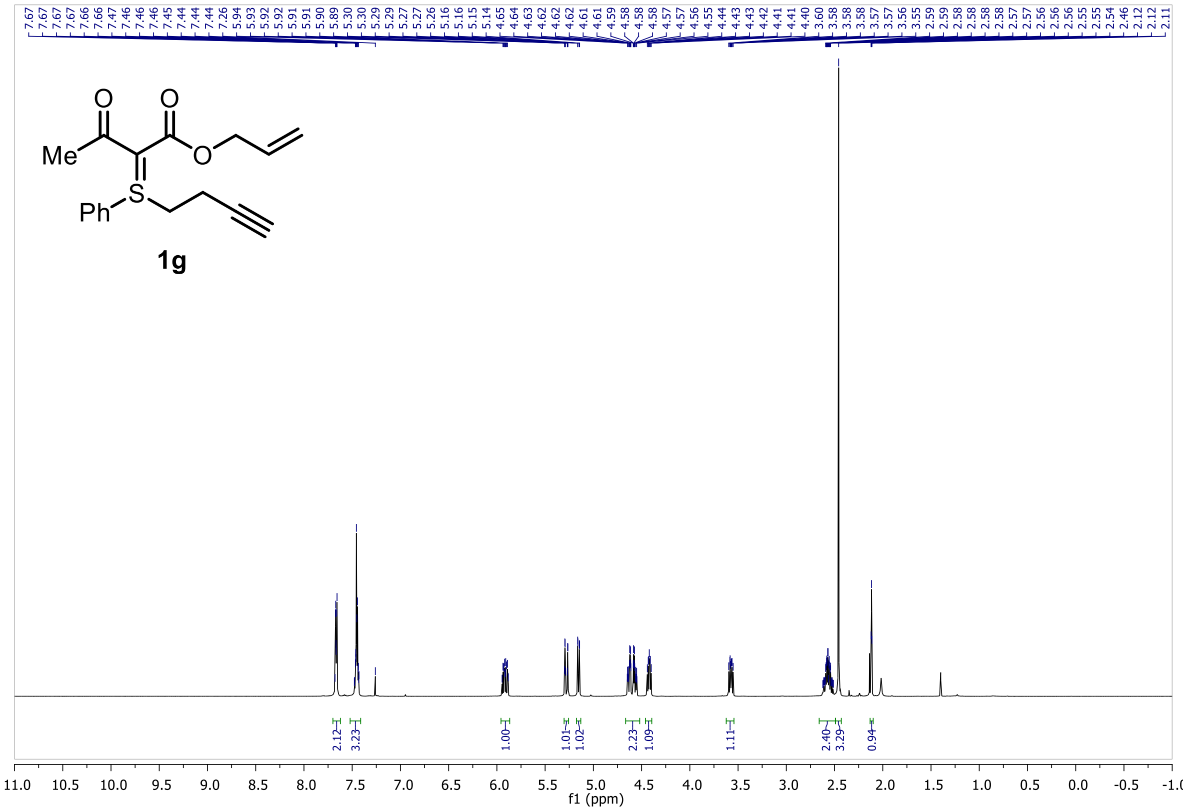

7.67 7.67 7.67 7.66 7.47 7.46 7.46 7.45 7.44 7.44 7.44 7.26 5.94 5.93 5.92 5.92 5.91 5.91 5.90 5.89 5.30 5.30 5.29 5.29 5.27 5.27 5.26 5.16 5.16 5.15 5.14 4.65 4.64 4.63 4.62 4.62 4.62 4.61 4.61 4.59 4.58 4.58 4.58 4.57 4.57 4.56 4.55 4.44 4.43 4.43 4.42 4.41 4.41 4.40 3.60 3.58 3.58 3.58 3.58 3.57 3.57 3.56 3.55 2.59 2.59 2.58 2.58 2.58 2.58 2.57 2.57 2.56 2.56 2.56 2.55 2.55 2.54 2.46 2.12 2.12 2.11
Me
O
O
O
S
Ph
1g
2.12 3.23 1.00 1.01 1.02 2.23 1.09 1.11 2.40 3.29 0.94
11.0 10.5 10.0 9.5 9.0 8.5 8.0 7.5 7.0 6.5 6.0 5.5 5.0 4.5 4.0 3.5 3.0 2.5 2.0 1.5 1.0 0.5 0.0 -0.5
f1 (ppm)

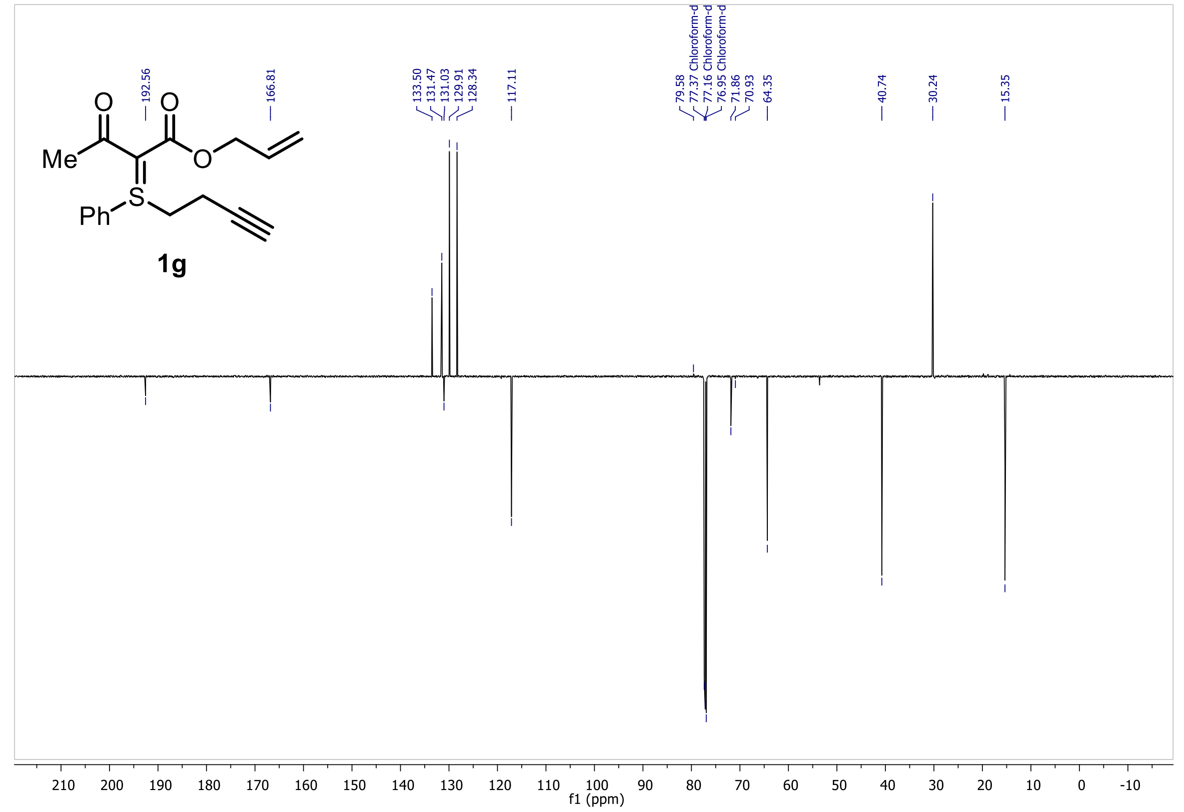

192.56
166.81
133.50 131.47 131.03 129.91 128.34
117.11
79.58 77.37 Chloroform-d 77.16 Chloroform-d 76.95 Chloroform-d 71.86 70.93
64.35
40.74
30.24
15.35
Me
O
O
O
S
Ph
1g
210 200 190 180 170 160 150 140 130 120 110 100 90 80 70 60 50 40 30 20 10 0 -10
f1 (ppm)

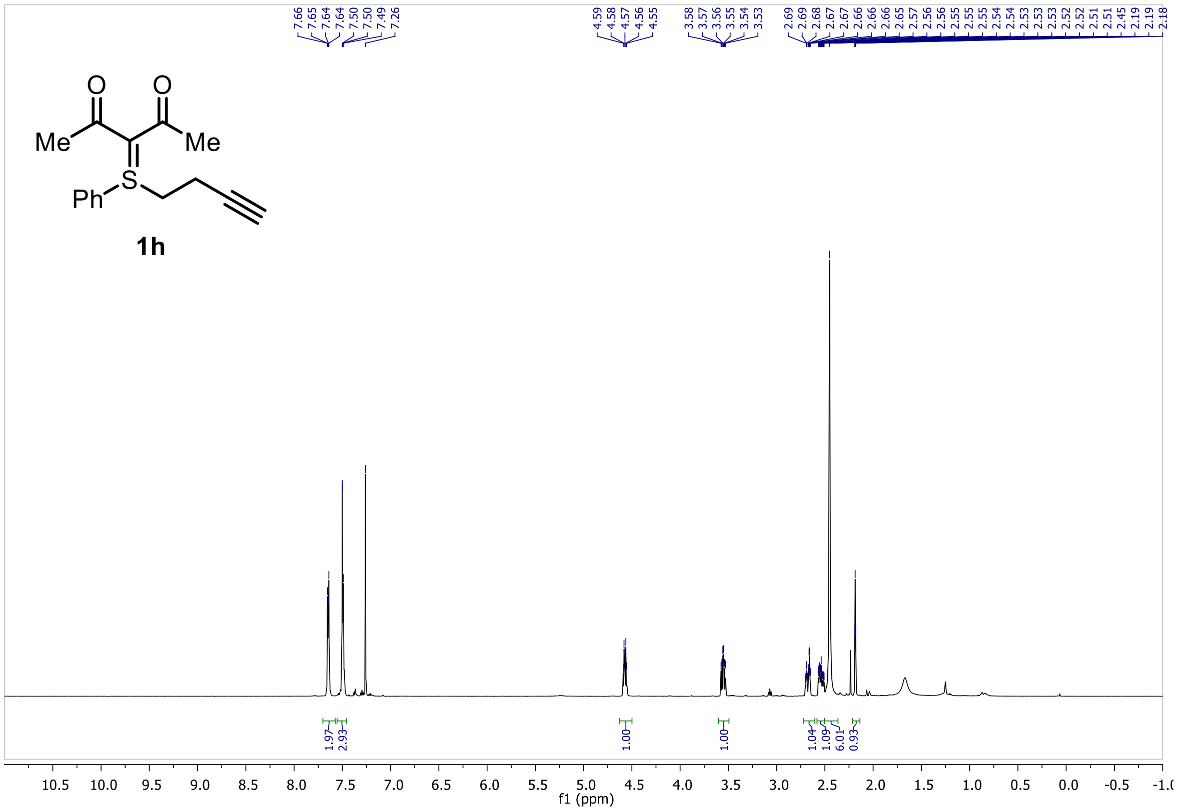

1h
7.66 7.65 7.64 7.64 7.50 7.50 7.49 7.26
4.59 4.58 4.57 4.56 4.55
3.58 3.57 3.56 3.55 3.54 3.53
2.69 2.69 2.68 2.67 2.67 2.66 2.66 2.66 2.65 2.57 2.56 2.56 2.55 2.55 2.55 2.54 2.54 2.53 2.53 2.53 2.52 2.52 2.51 2.51 2.45 2.19 2.18
1.97 2.93 1.00 1.00 1.04 1.09 6.01 0.93
f1 (ppm)

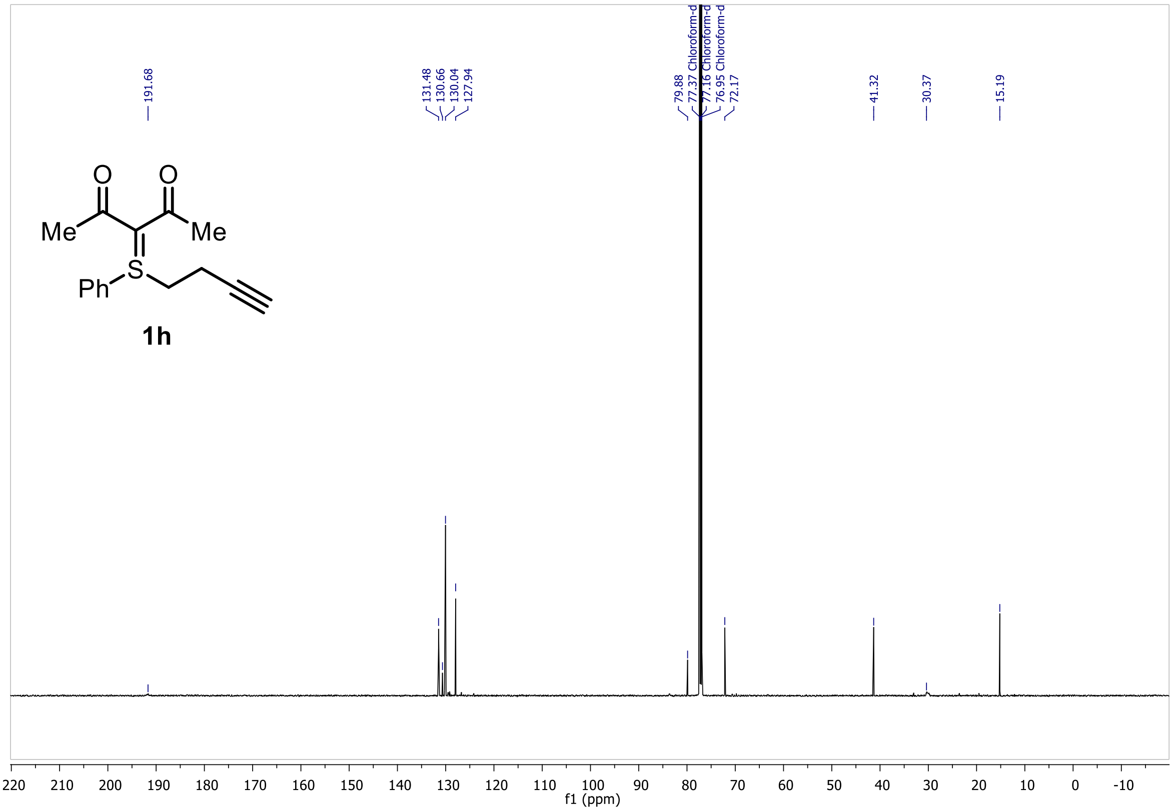

1h
191.68
131.48 130.66 130.04 127.94
79.88 77.37 Chloroform-d 77.16 Chloroform-d 76.95 Chloroform-d 72.17
41.32
30.37
15.19
f1 (ppm)

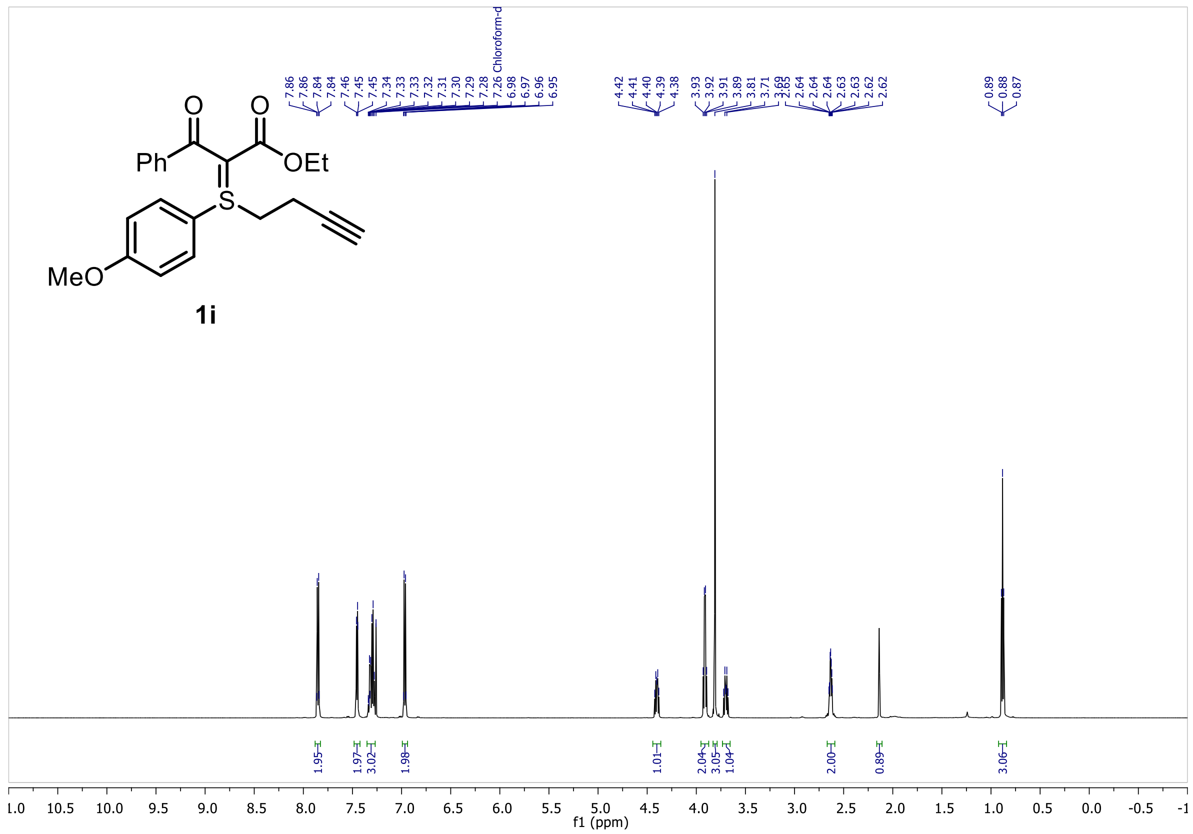
1i
f1 (ppm)

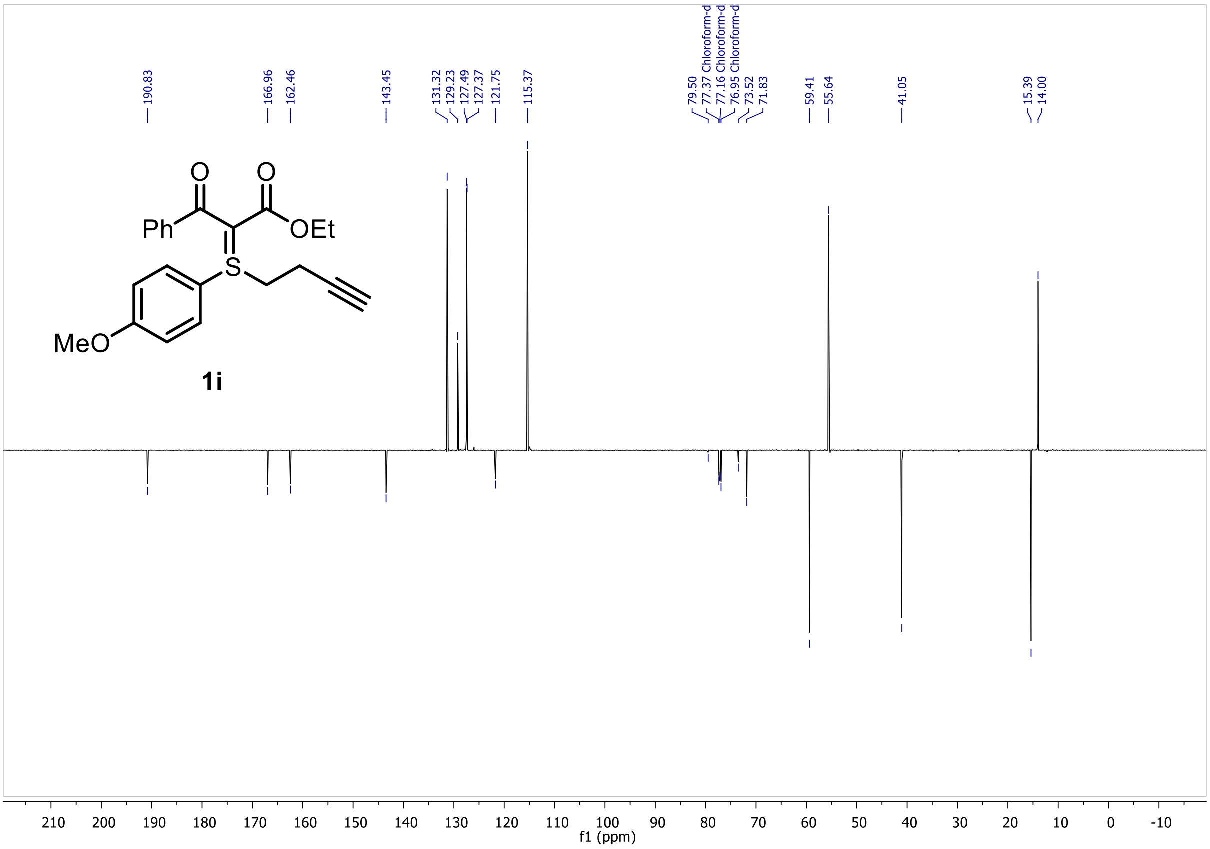
1i
f1 (ppm)

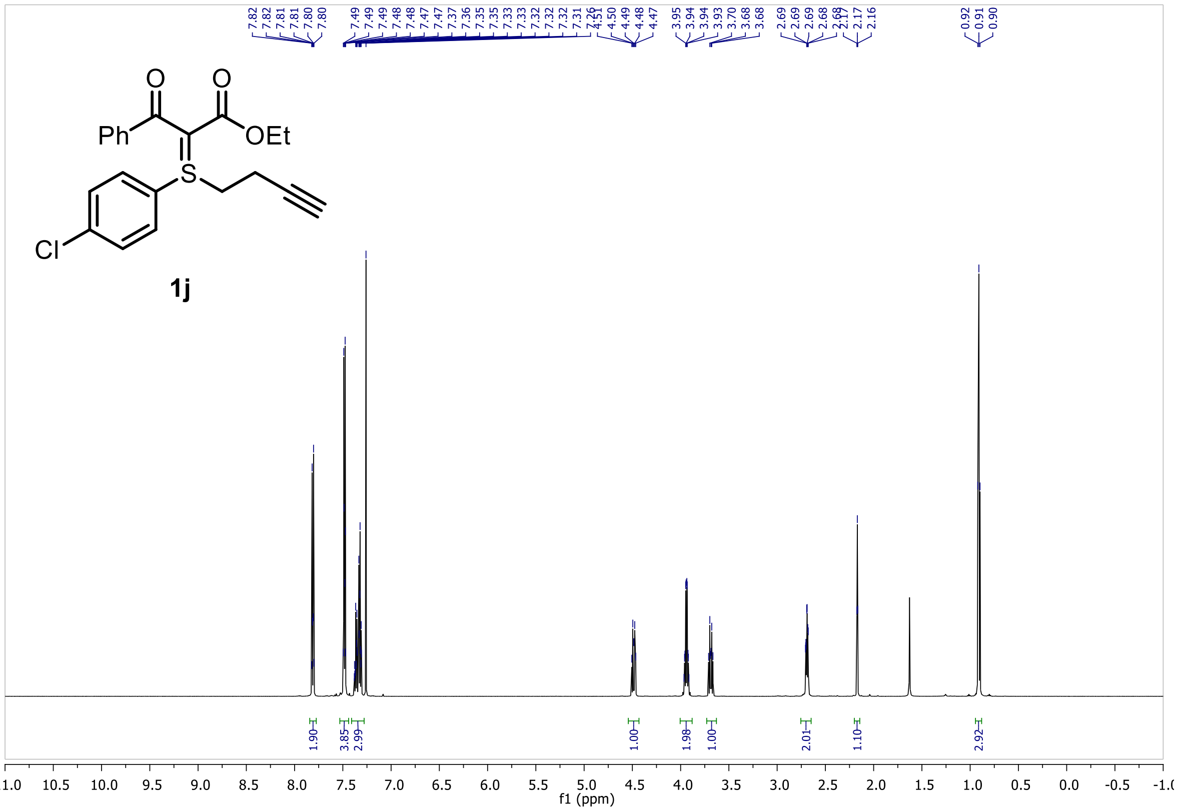
7.82
7.82
7.81
7.80
7.80
7.49
7.49
7.49
7.48
7.48
7.47
7.47
7.37
7.36
7.35
7.35
7.33
7.33
7.32
7.32
7.32
7.31
7.26
4.51
4.50
4.49
4.48
4.47
3.95
3.94
3.94
3.93
3.70
3.68
3.68
2.69
2.69
2.69
2.68
2.68
2.17
2.17
2.16
0.92
0.91
0.90
O
O
Ph
OEt
S
Cl
1j
1.90
3.85
2.99
1.00
1.98
1.00
2.01
1.10
2.92
f1 (ppm)

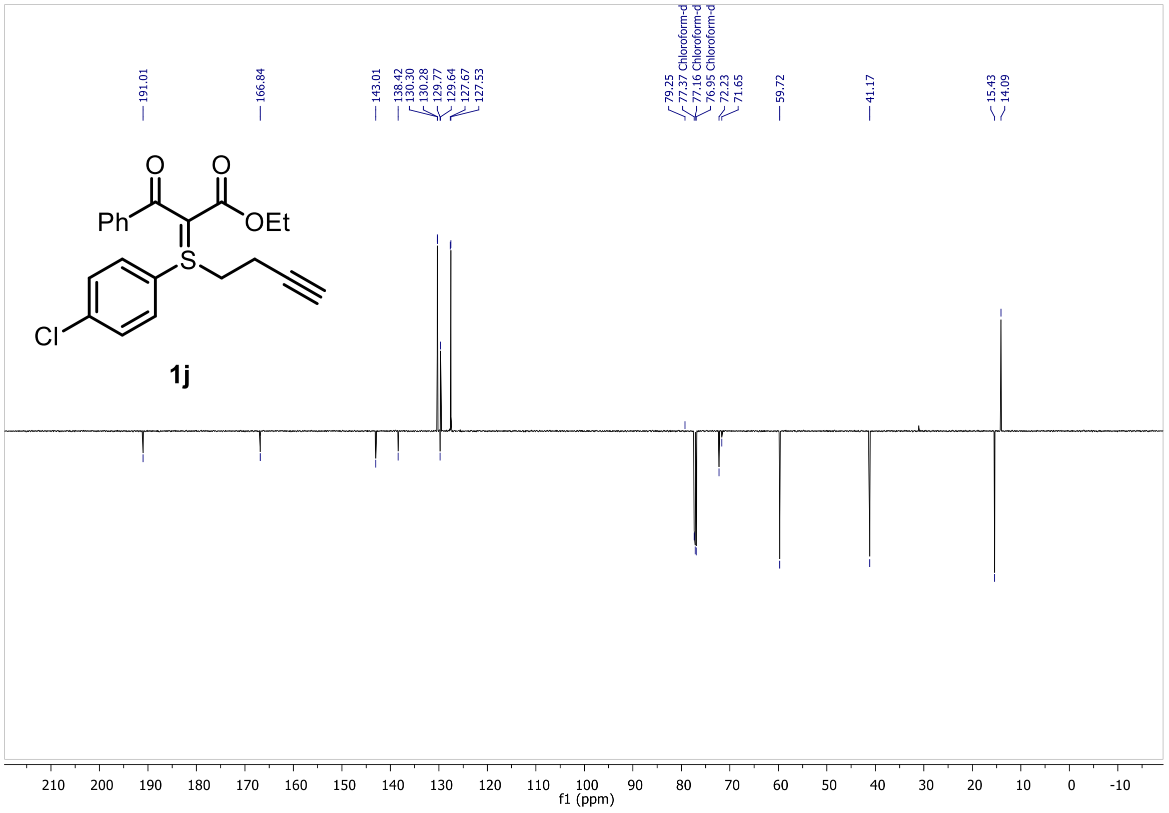
191.01
166.84
143.01
138.42
130.30
130.28
129.77
129.64
127.67
127.53
79.25
77.37 Chloroform-d
77.16 Chloroform-d
76.95 Chloroform-d
72.23
71.65
59.72
41.17
15.43
14.09
O
O
Ph
OEt
S
Cl
1j
f1 (ppm)

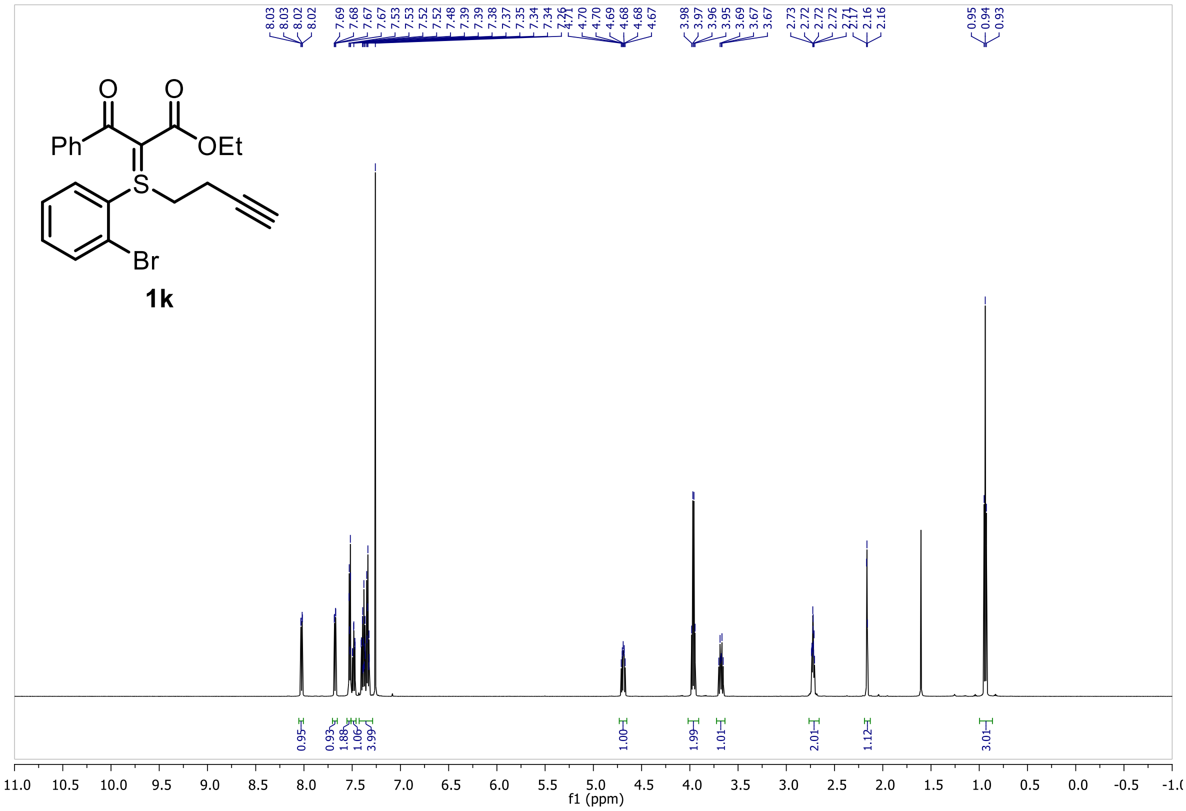

8.03
8.03
8.02
8.02
7.69
7.68
7.67
7.67
7.53
7.53
7.52
7.52
7.48
7.39
7.39
7.38
7.37
7.35
7.34
7.34
7.26
4.71
4.70
4.70
4.69
4.68
4.68
4.67
3.98
3.97
3.96
3.95
3.69
3.67
3.67
2.73
2.72
2.72
2.72
2.71
2.17
2.16
2.16
0.95
0.94
0.93
Ph
O
O
OEt
S
Br
1k
0.95
0.93
1.88
1.06
3.99
1.00
1.99
1.01
2.01
1.12
3.01
f1 (ppm)

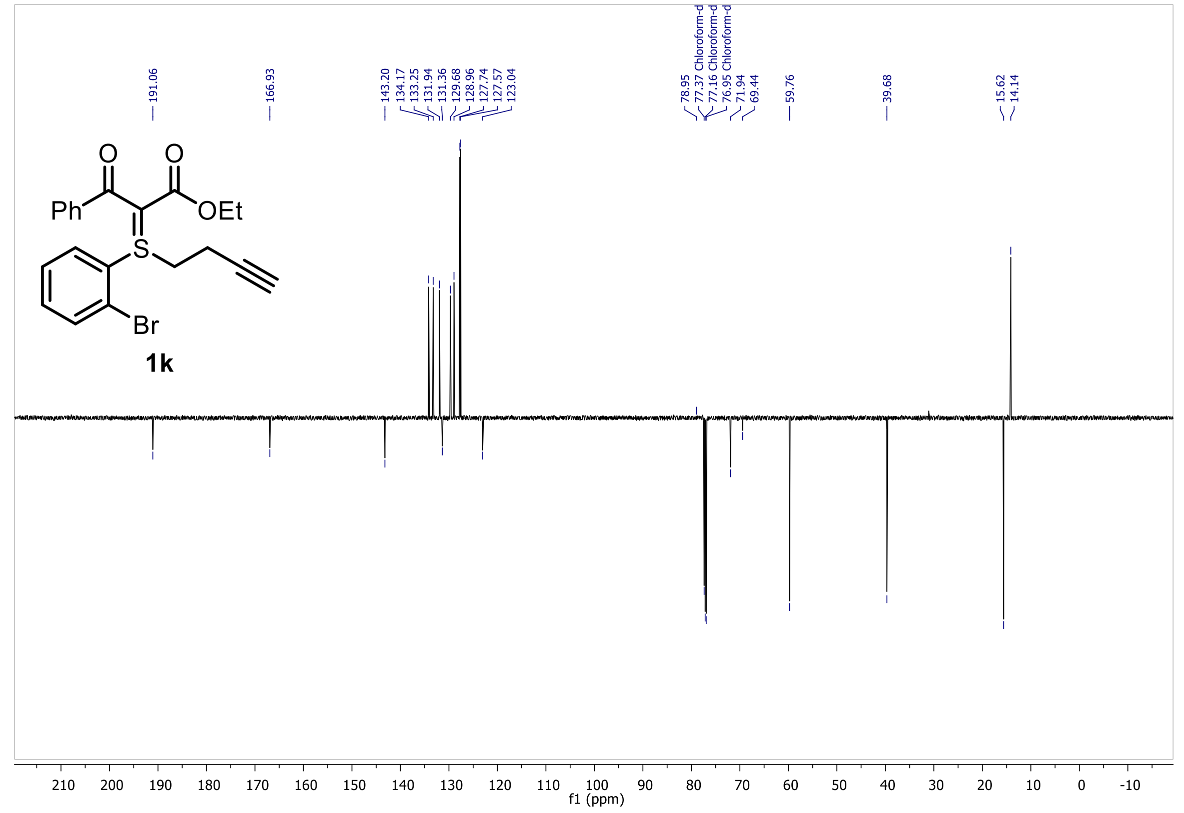

191.06
166.93
143.20
134.17
133.25
131.94
131.36
129.68
128.96
127.74
127.57
123.04
78.95
77.37 Chloroform-d
77.16 Chloroform-d
76.95 Chloroform-d
71.94
69.44
59.76
39.68
15.62
14.14
Ph
O
O
OEt
S
Br
1k
f1 (ppm)

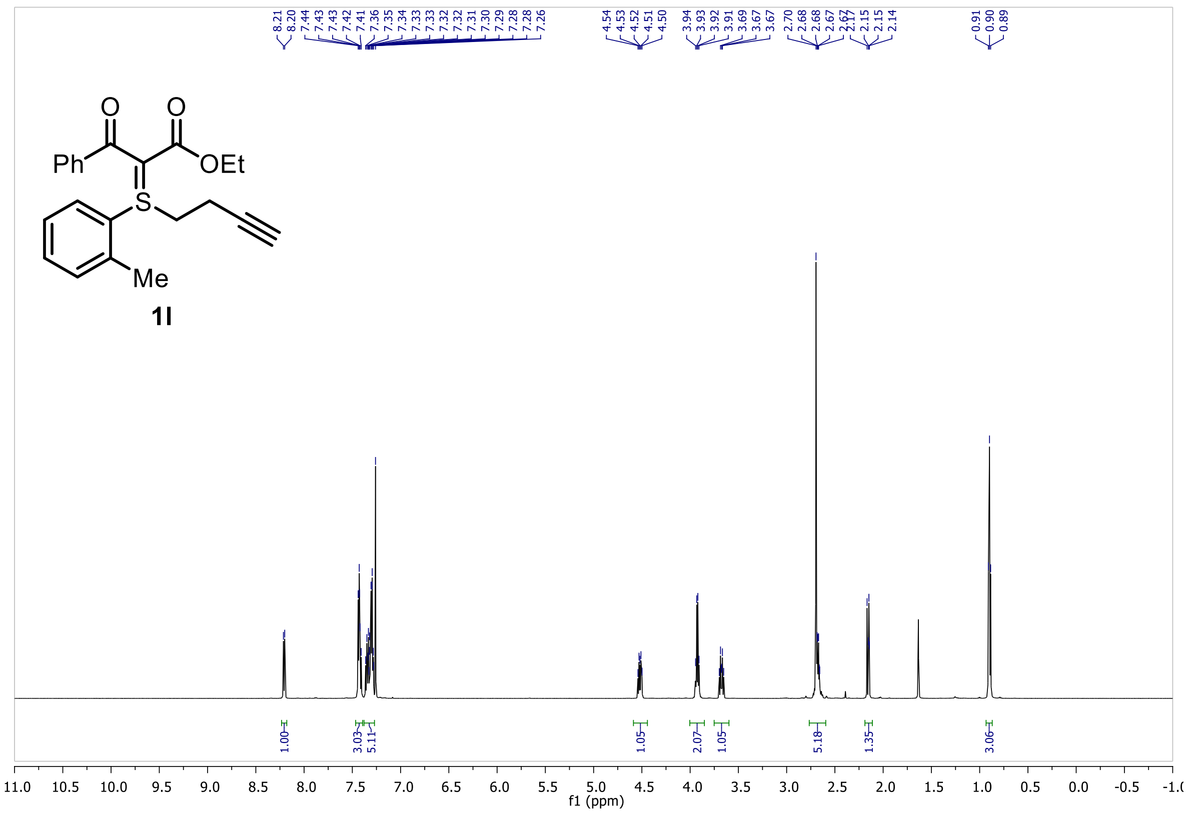
8.21
8.20
7.44
7.43
7.43
7.42
7.41
7.36
7.35
7.34
7.33
7.33
7.32
7.32
7.31
7.30
7.29
7.28
7.28
7.26
4.54
4.53
4.52
4.51
4.50
3.94
3.93
3.92
3.91
3.69
3.67
3.67
2.70
2.68
2.68
2.67
2.67
2.17
2.15
2.15
2.14
0.91
0.90
0.89
Ph
O
O
OEt
S
Me
1l
1.00
3.03
5.11
1.05
2.07
1.05
5.18
1.35
3.06
f1 (ppm)

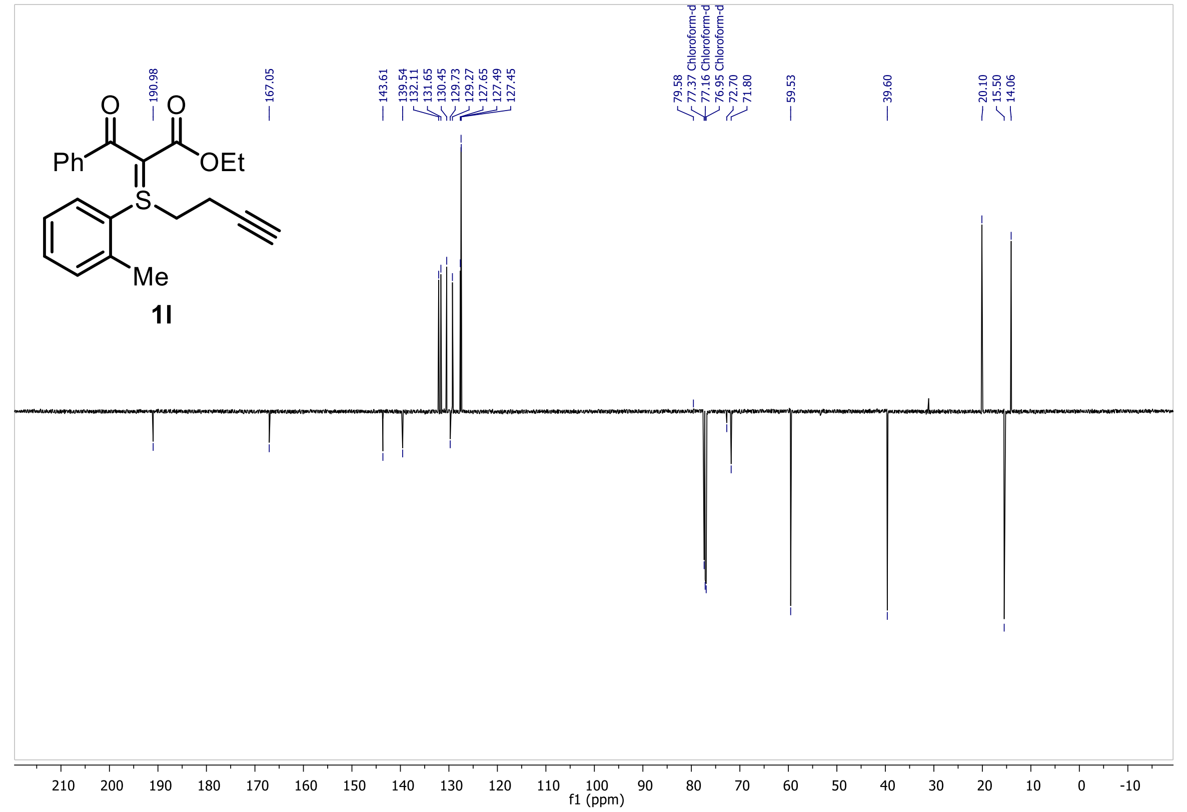
190.98
167.05
143.61
139.54
132.11
131.65
130.45
129.73
129.27
127.65
127.49
127.45
79.58
77.37 Chloroform-d
77.16 Chloroform-d
76.95 Chloroform-d
72.70
71.80
59.53
39.60
20.10
15.50
14.06
Ph
O
O
OEt
S
Me
1l
f1 (ppm)

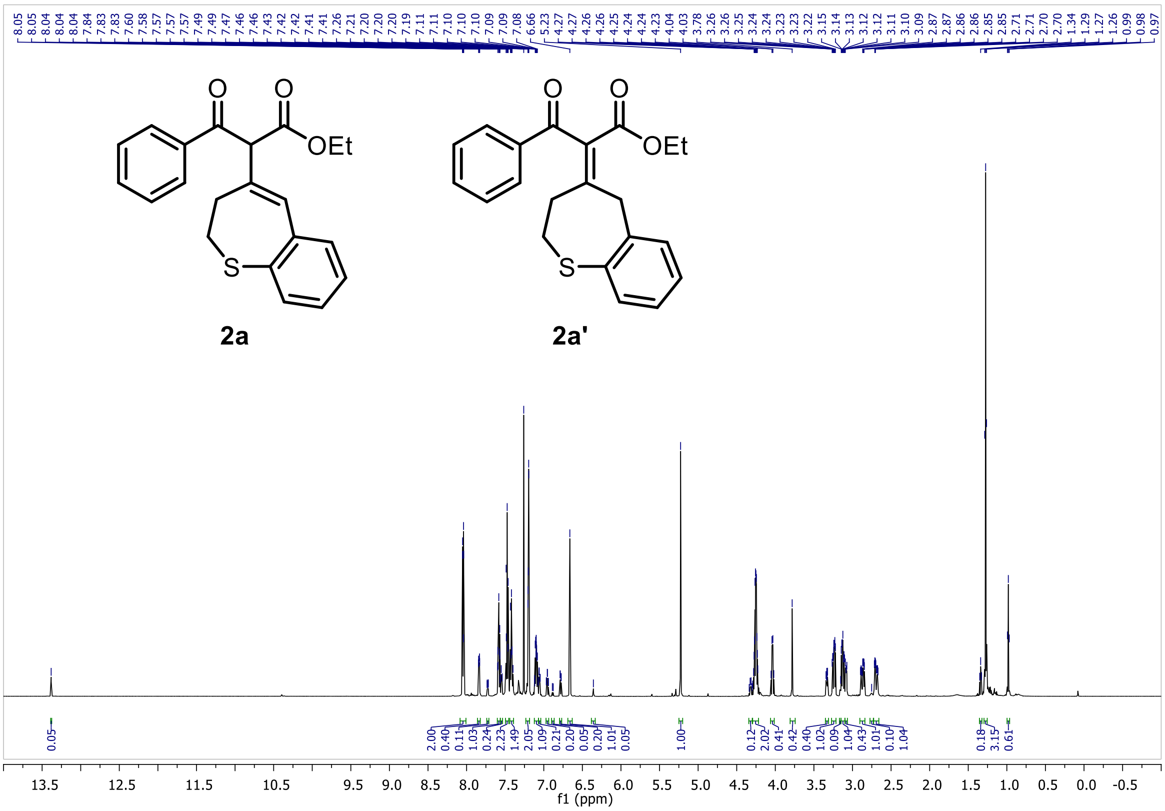

2a
2a'
OEt
f1 (ppm)

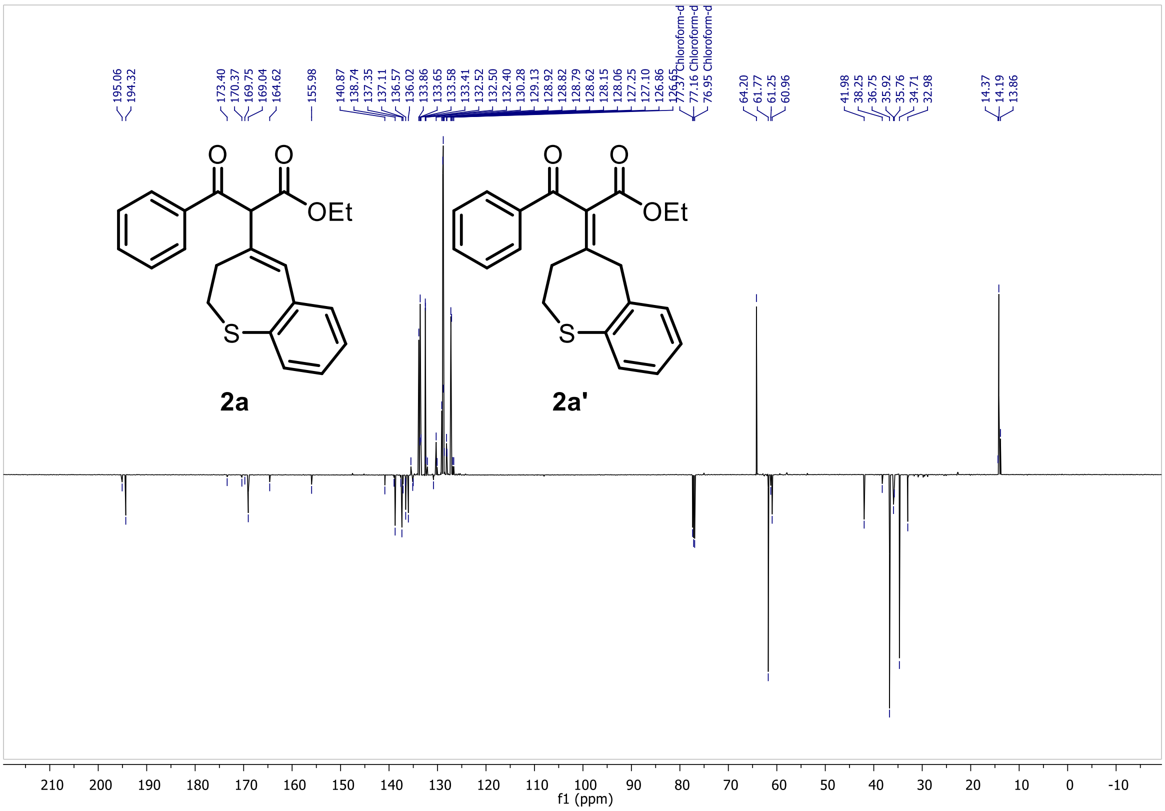

2a
2a'
OEt
f1 (ppm)

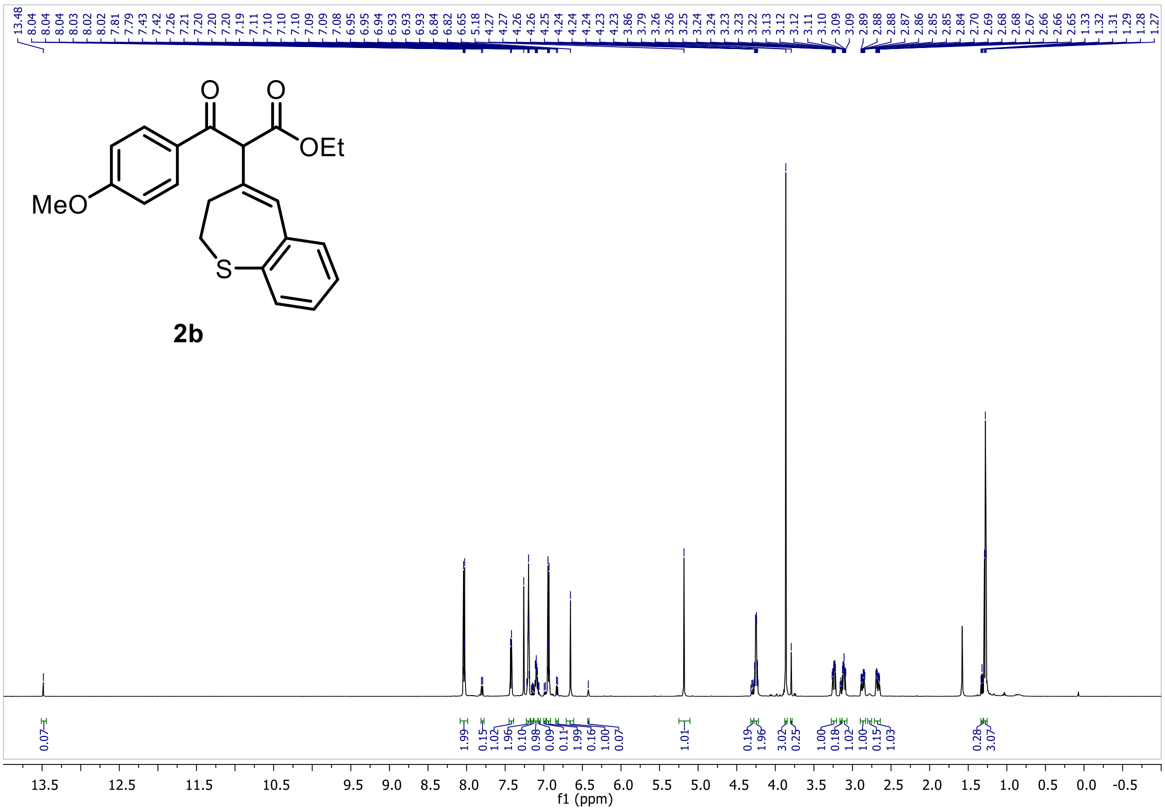
MeO
OEt
2b
f1 (ppm)

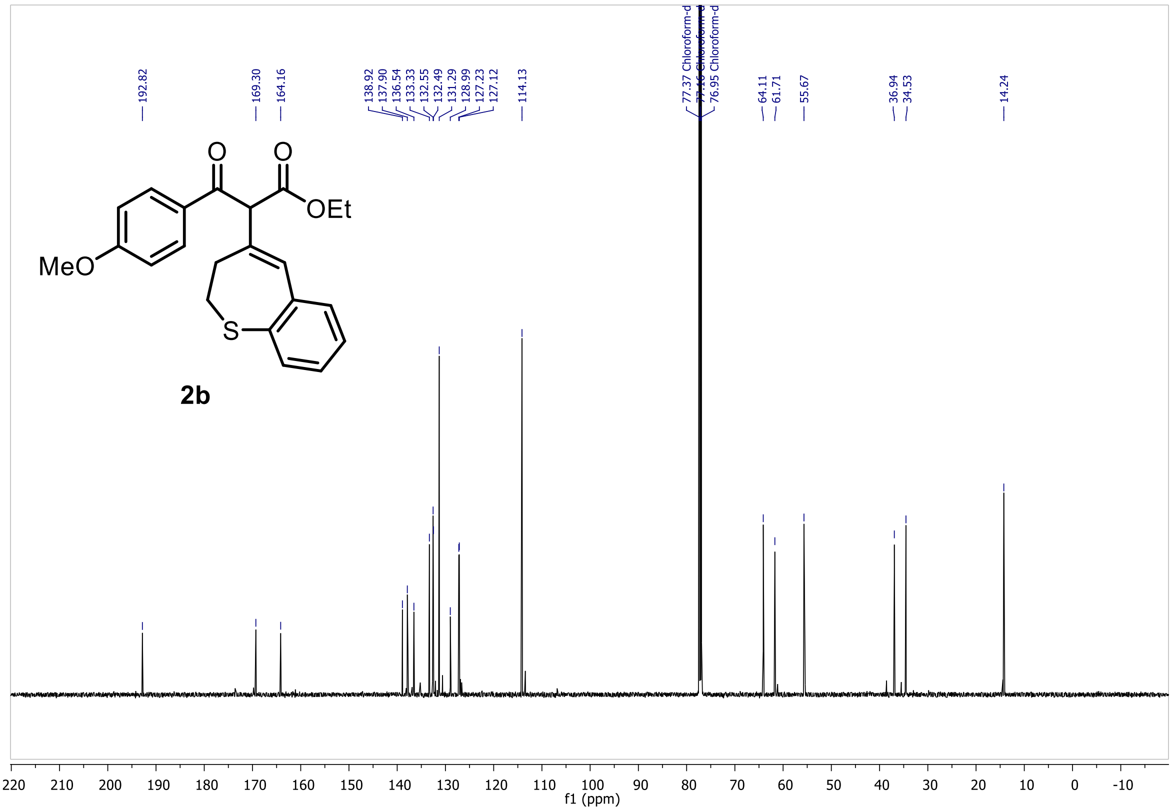
MeO
OEt
2b
192.82
169.30
164.16
114.13
64.11
61.71
55.67
36.94
34.53
14.24
f1 (ppm)

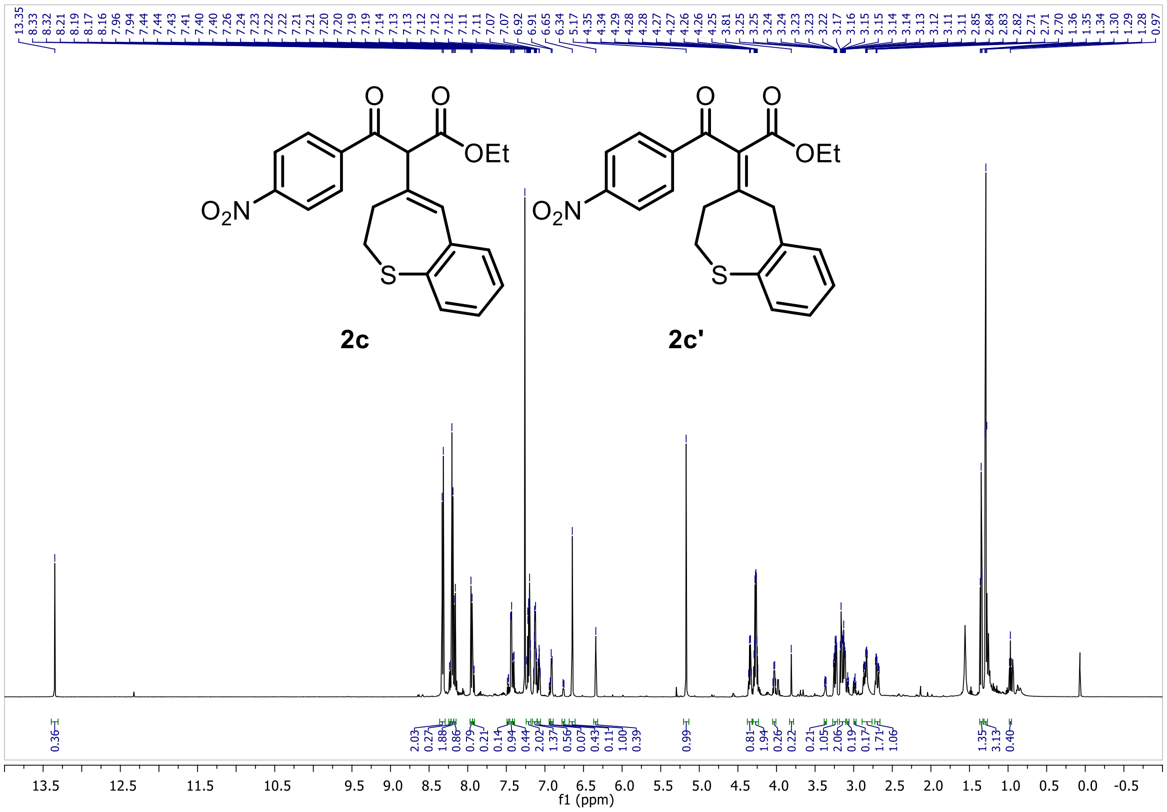
2c
2c'
OEt
f1 (ppm)

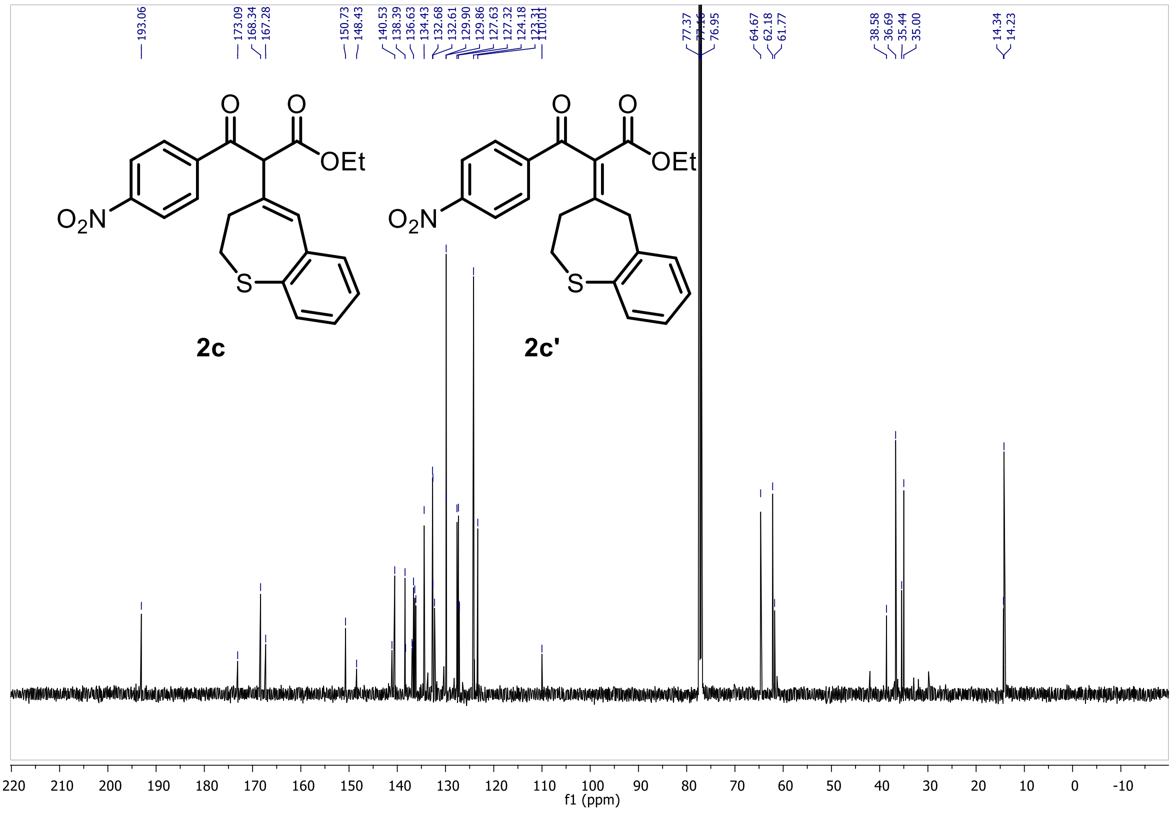
2c
2c'
OEt
f1 (ppm)

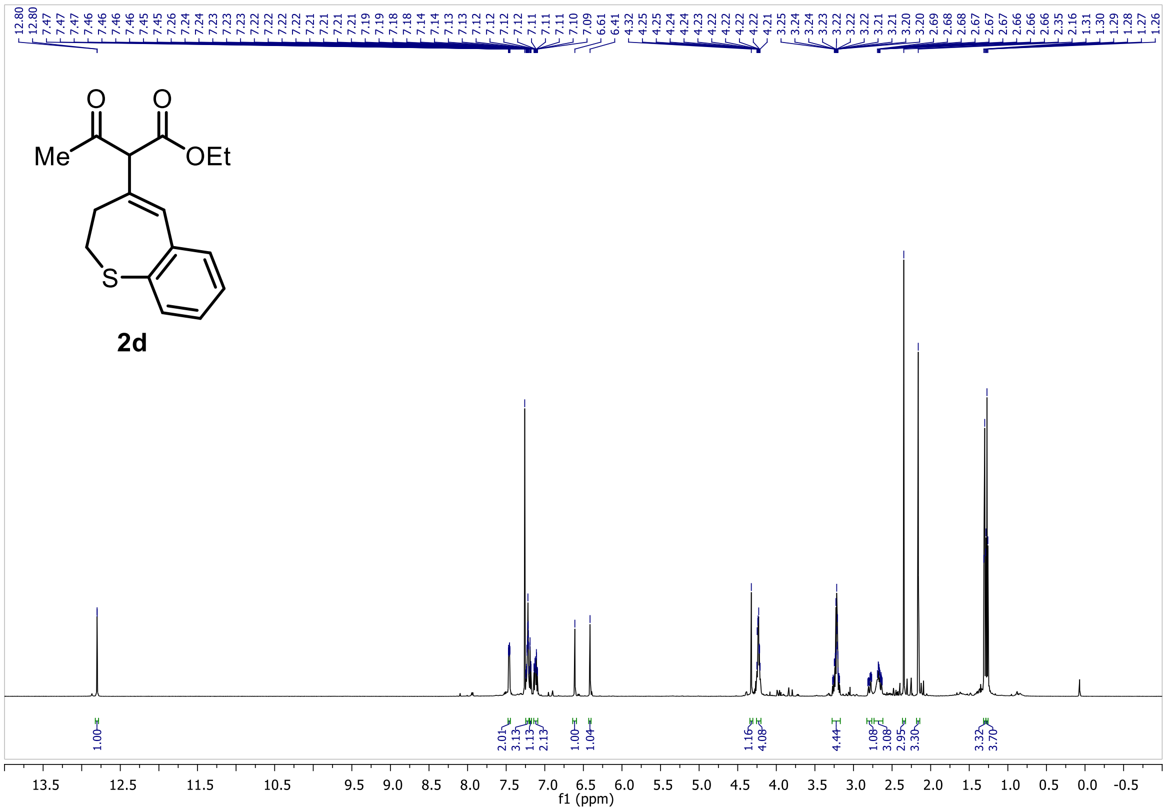
Me
OEt
S
2d
f1 (ppm)

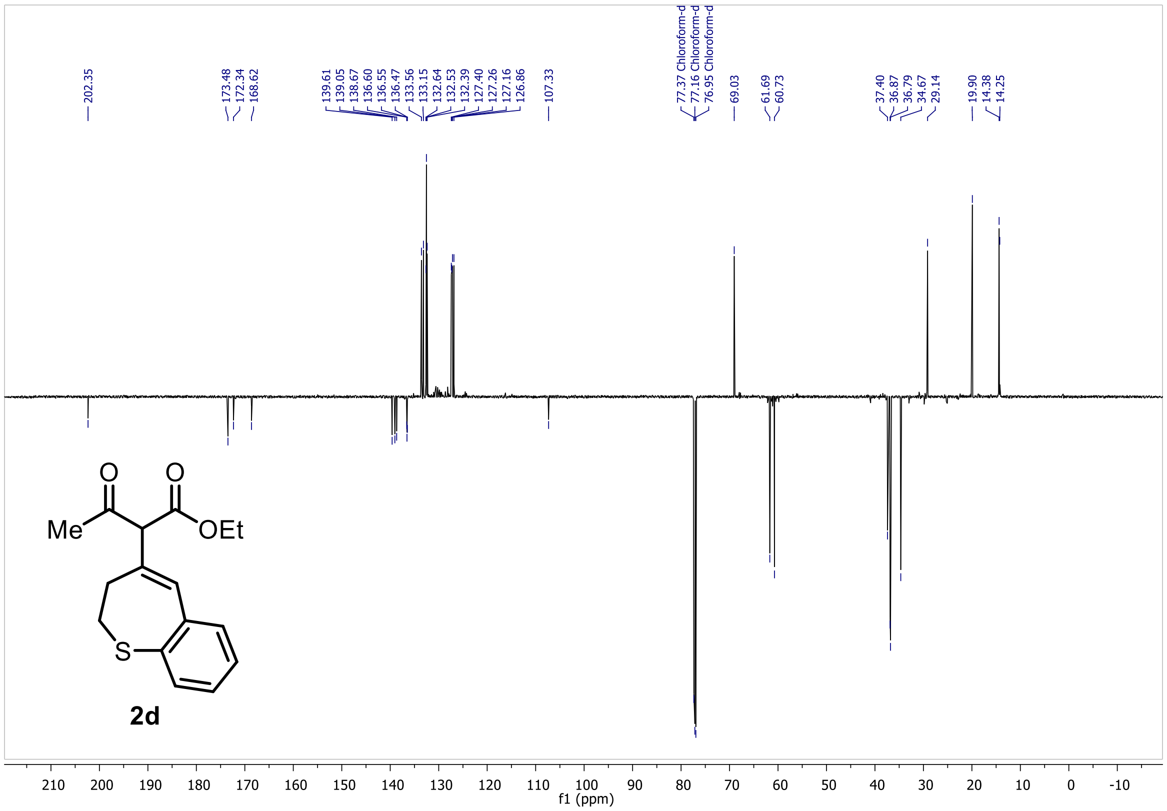
202.35
173.48
172.34
168.62
107.33
77.37 Chloroform-d
77.16 Chloroform-d
76.95 Chloroform-d
69.03
61.69
60.73
37.40
36.87
36.79
34.67
29.14
19.90
14.38
14.25
Me
OEt
S
2d
f1 (ppm)

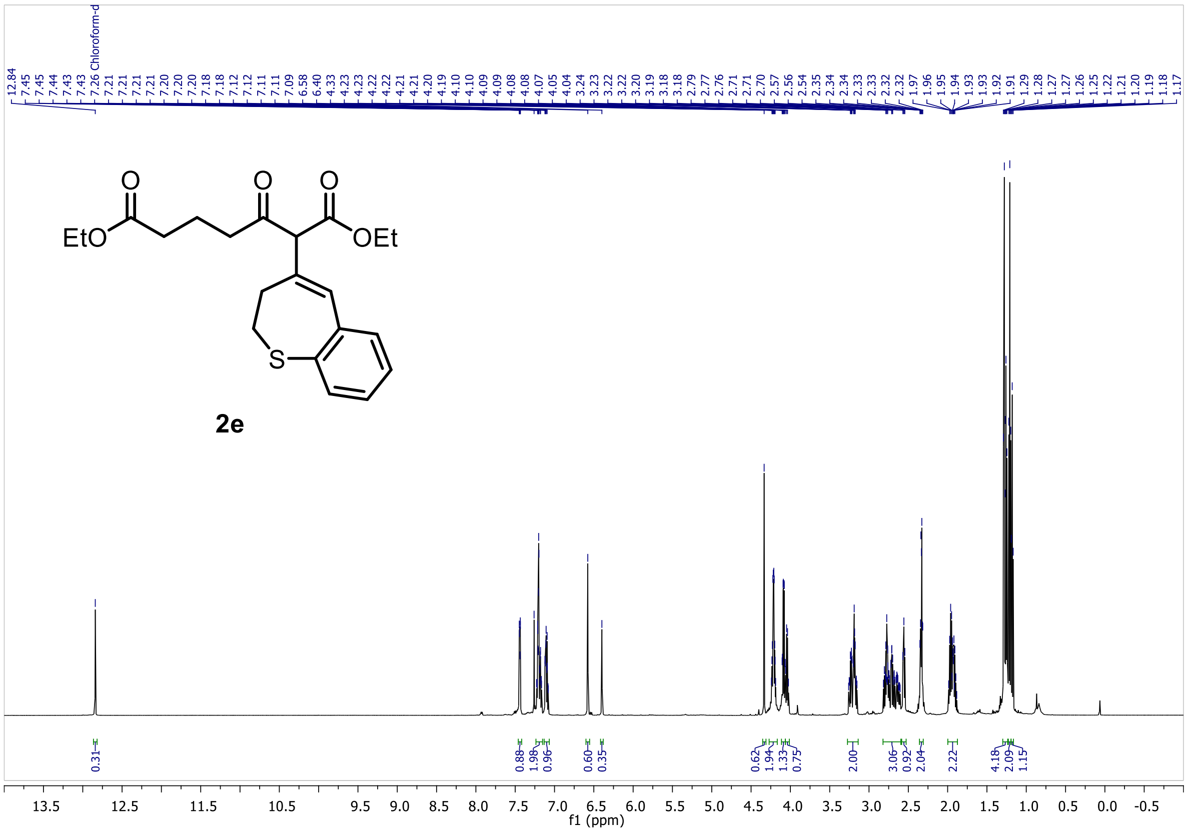

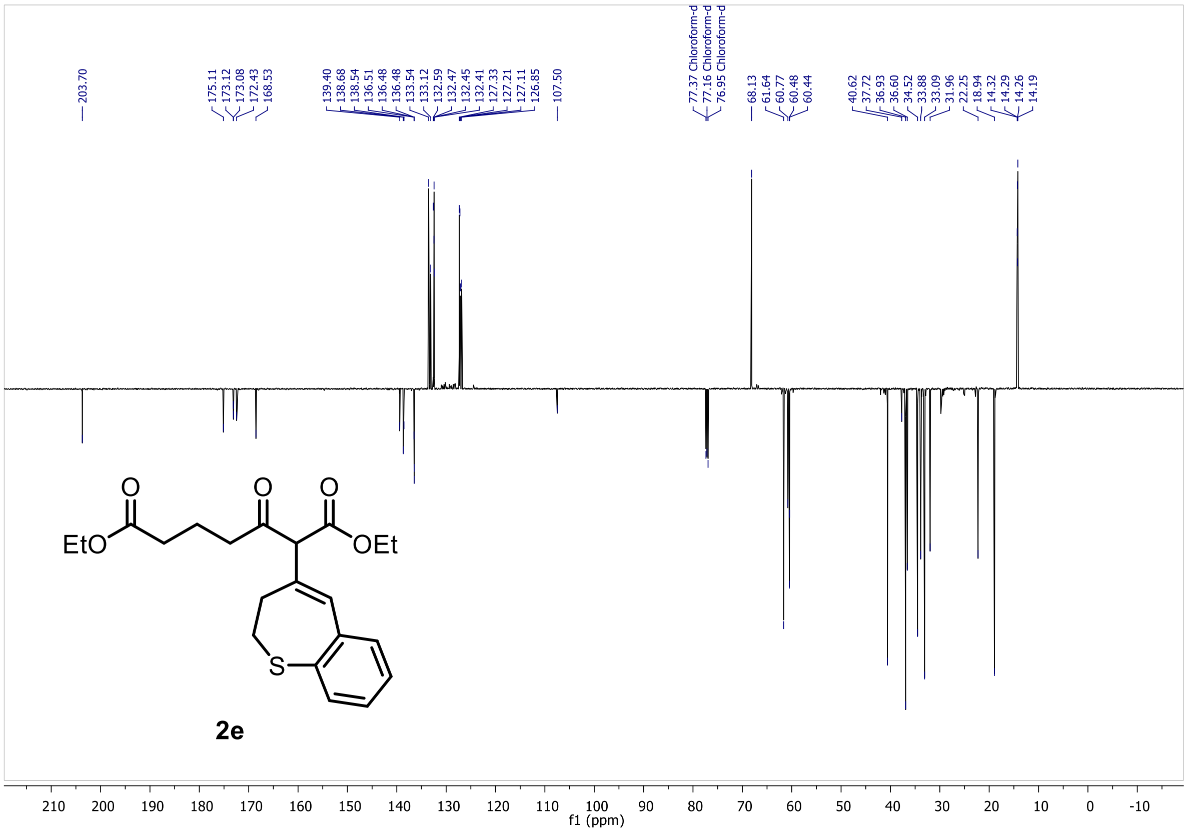

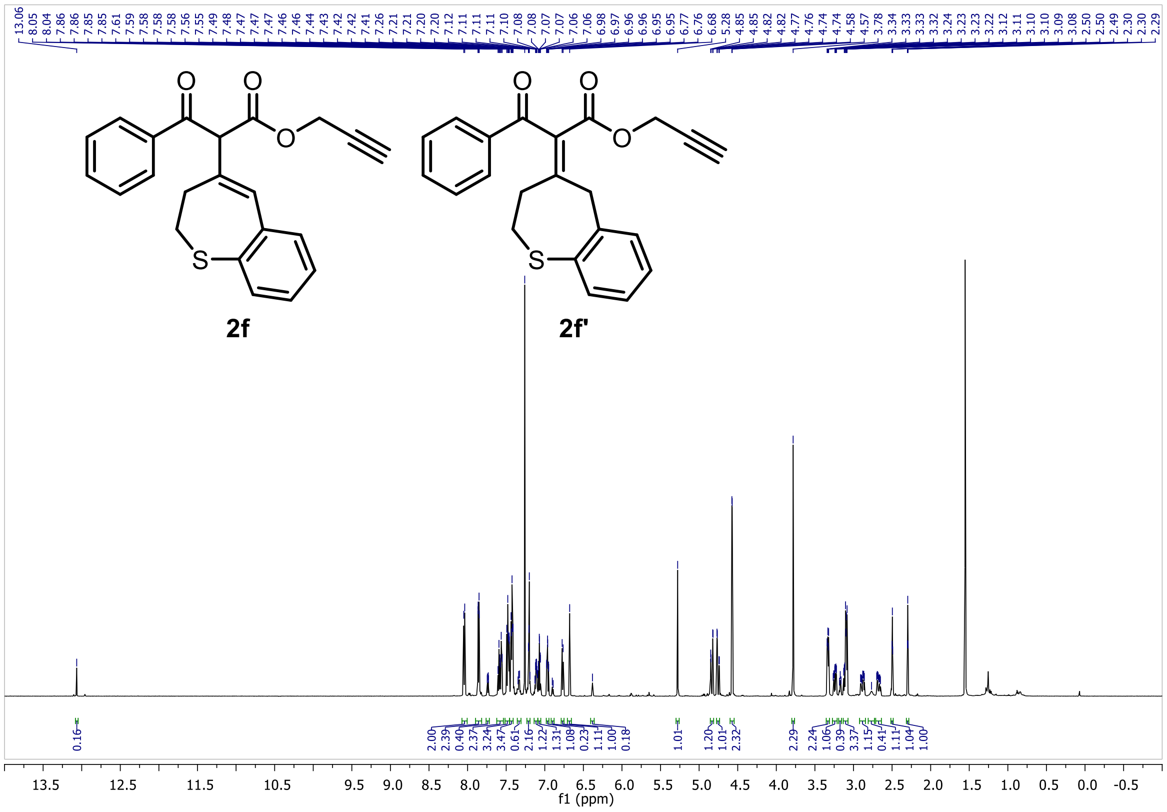

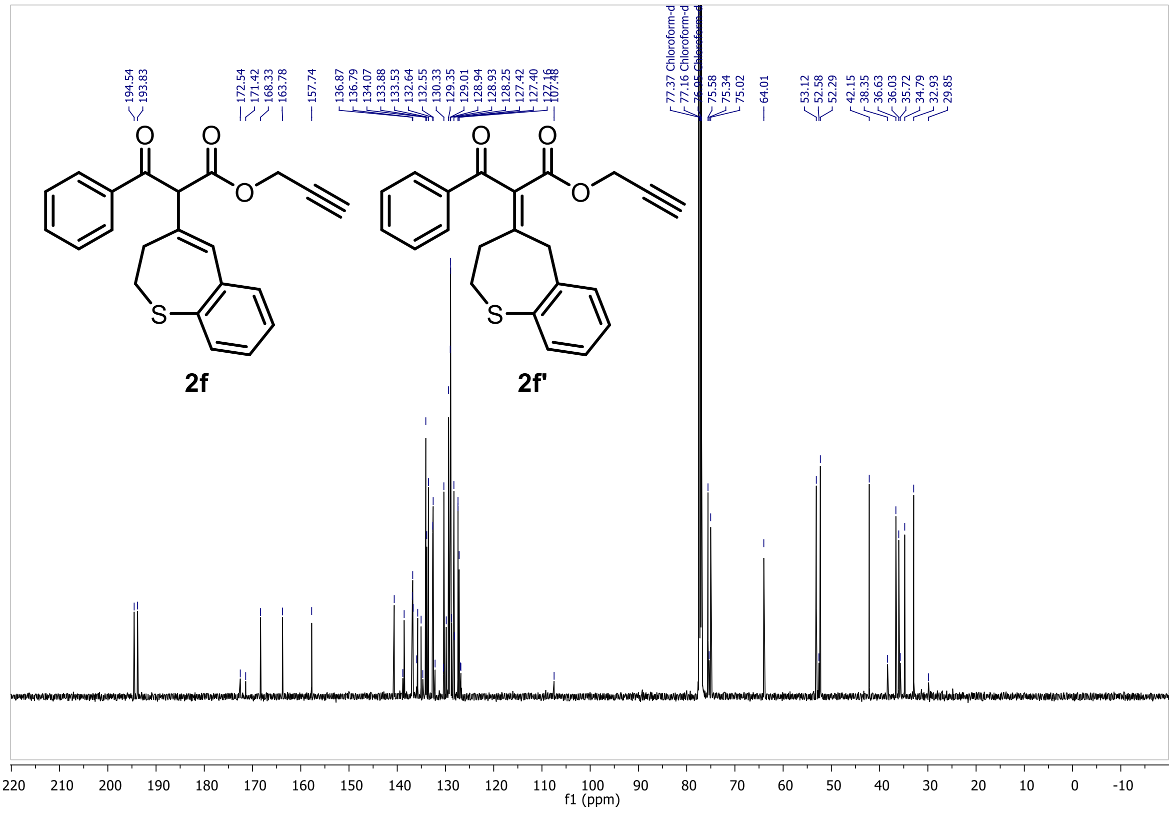

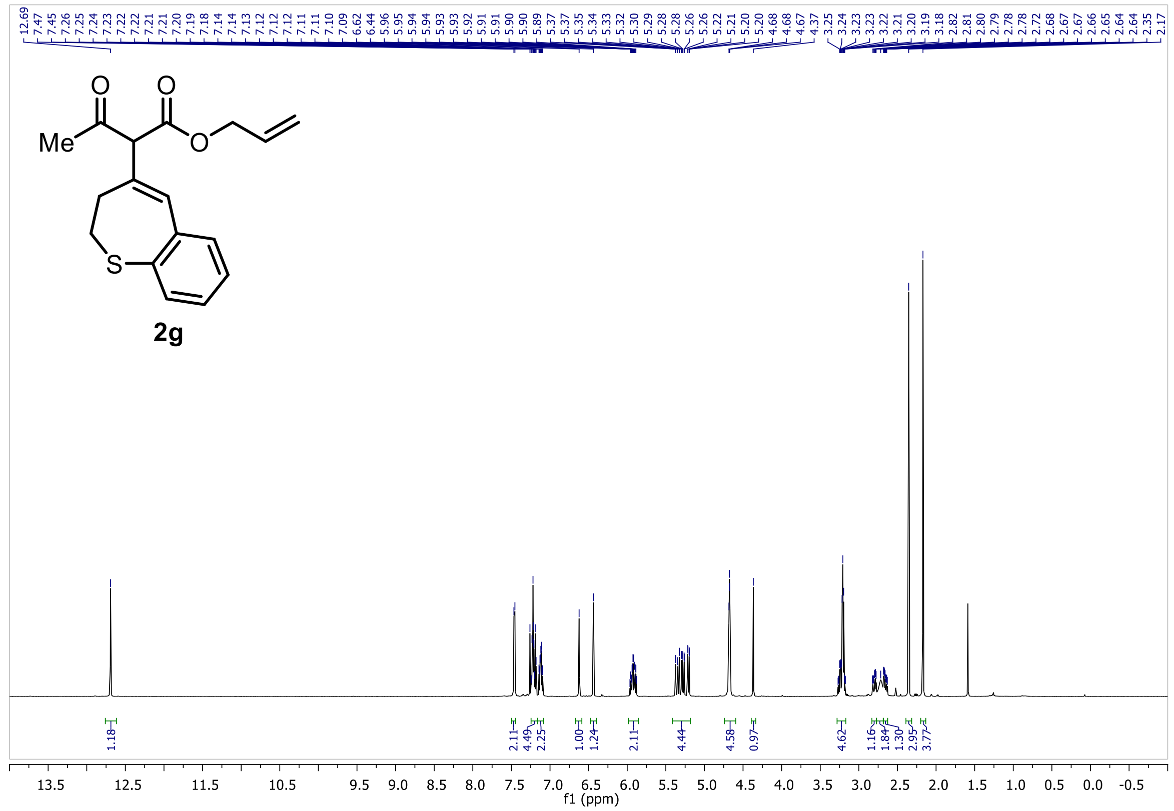

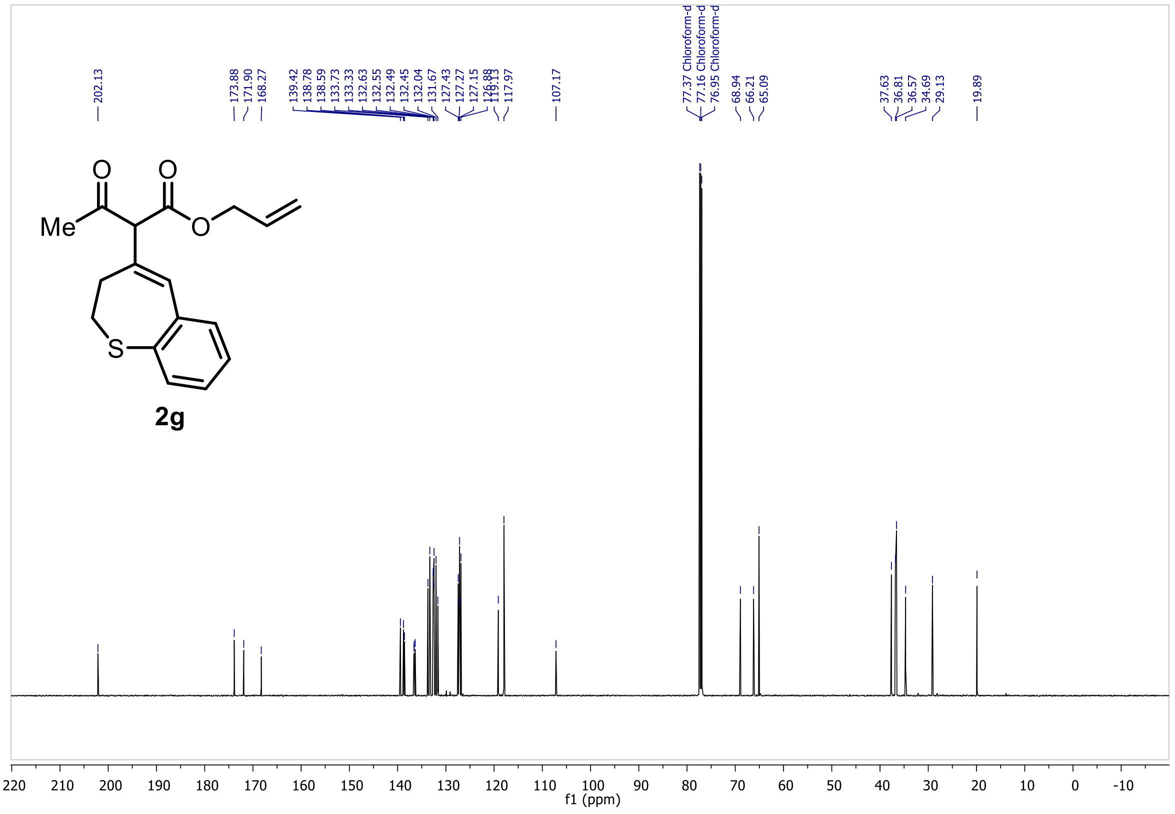

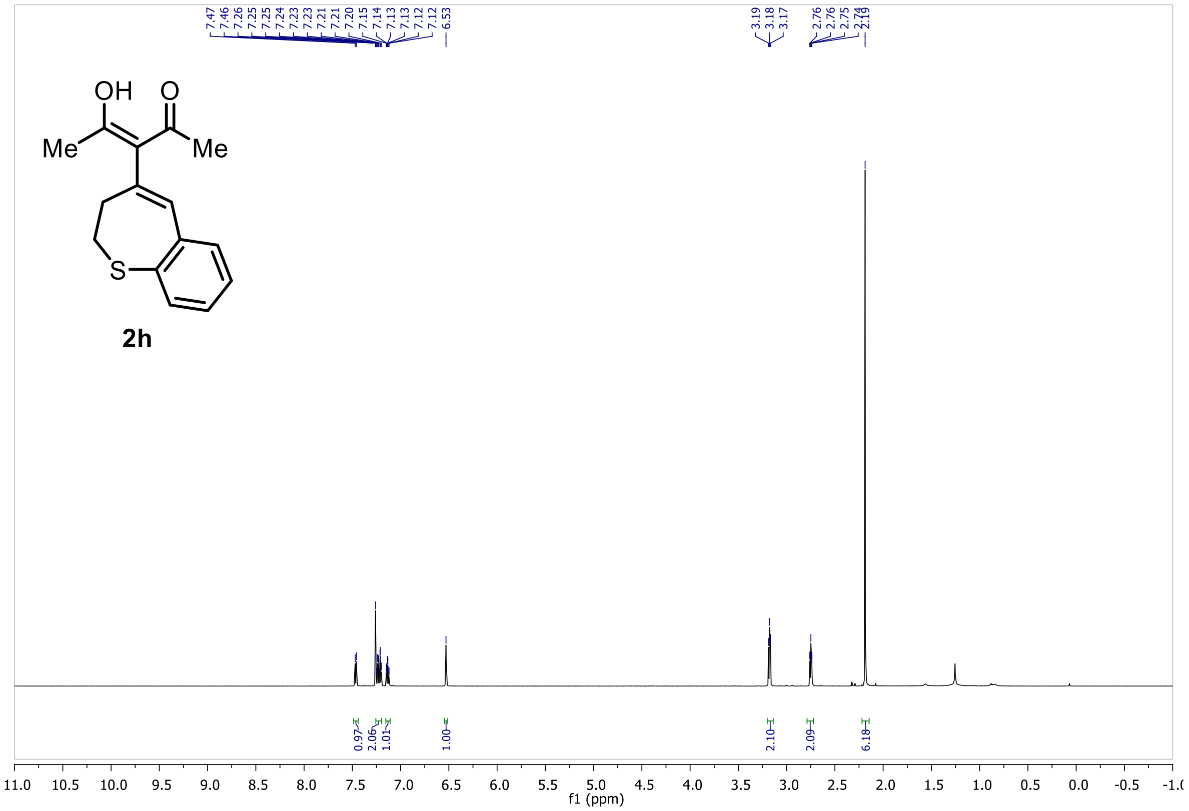
7.47
7.46
7.26
7.25
7.25
7.24
7.23
7.23
7.21
7.21
7.20
7.15
7.14
7.13
7.13
7.12
6.53
3.19
3.18
3.17
2.76
2.76
2.75
2.74
2.19
OH
O
Me
Me
S
2h
0.97
2.06
1.01
1.00
2.10
2.09
6.18
f1 (ppm)

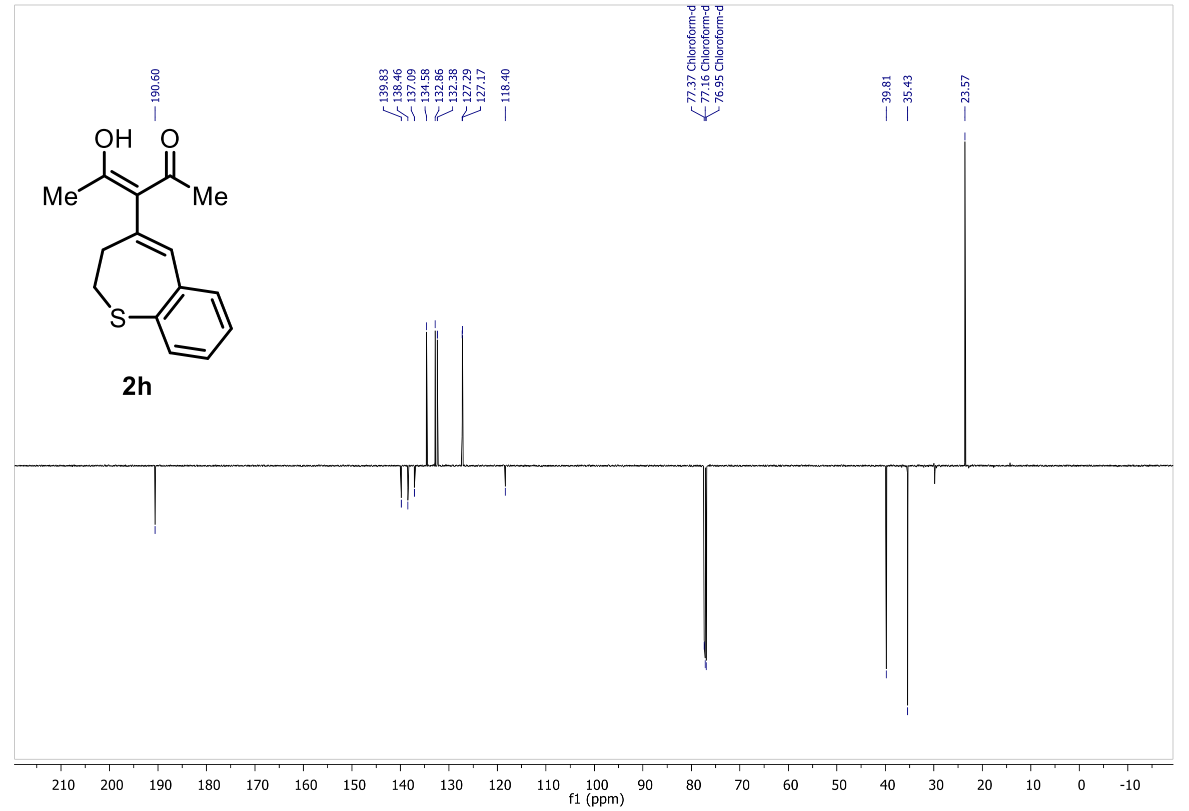
190.60
139.83
138.46
137.09
134.58
132.86
132.38
127.29
127.17
118.40
77.37 Chloroform-d
77.16 Chloroform-d
76.95 Chloroform-d
39.81
35.43
23.57
OH
O
Me
Me
S
2h
f1 (ppm)

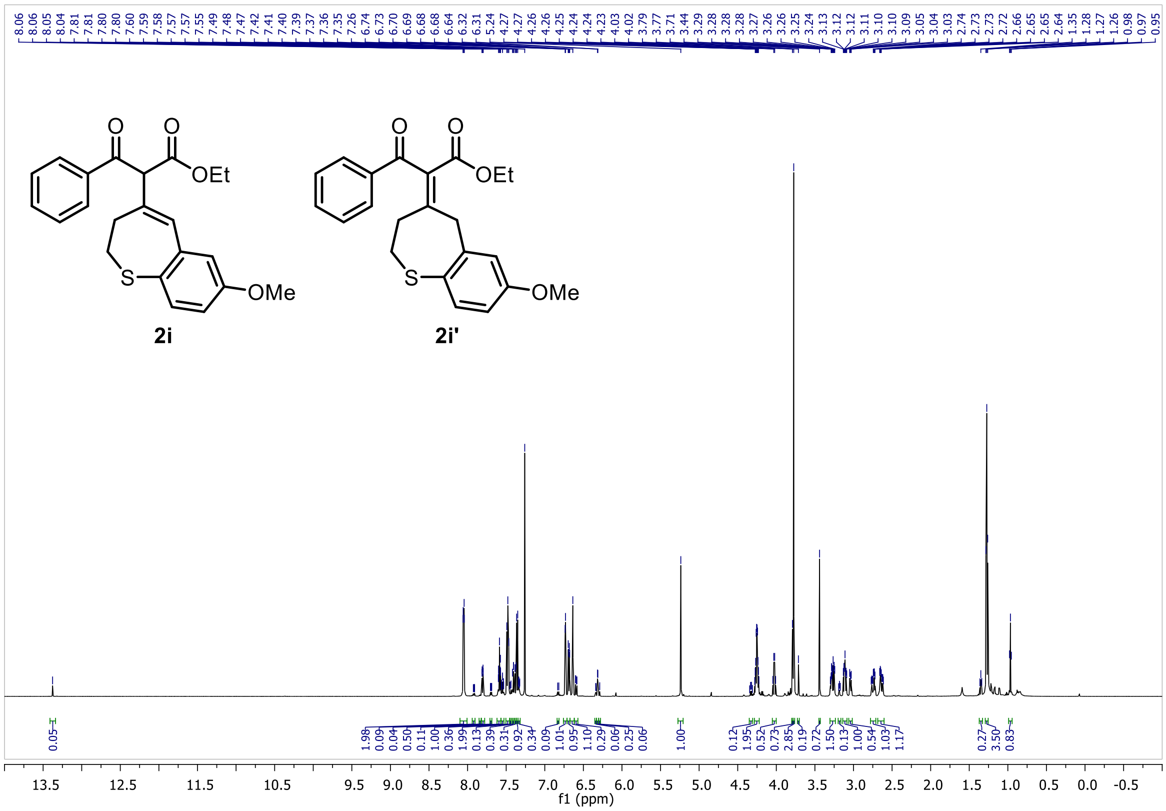
8.06 8.06 8.05 8.04 7.81 7.80 7.60 7.59 7.58 7.57 7.57 7.55 7.49 7.48 7.47 7.42 7.41 7.40 7.39 7.37 7.36 7.35 7.26 6.74 6.73 6.70 6.69 6.68 6.68 6.64 6.32 6.31 5.24 4.27 4.27 4.26 4.26 4.25 4.24 4.24 4.23 4.03 4.02 3.79 3.77 3.71 3.44 3.29 3.28 3.28 3.27 3.26 3.26 3.25 3.24 3.13 3.12 3.12 3.11 3.10 3.10 3.09 3.05 3.04 3.03 2.74 2.73 2.73 2.72 2.66 2.65 2.65 2.64 1.35 1.28 1.27 1.26 0.98 0.97 0.95
O
O
OEt
S
OMe
2i
2i'
0.05
1.98 0.09 0.04 0.50 0.11 1.00 0.36 1.99 0.13 0.39 0.31 0.92 0.34 0.09 1.01 0.95 1.10 0.29 0.06 0.25 0.06
1.00
0.12 1.95 0.52 0.73 2.85 0.19 0.72 1.50 0.13 1.00 0.54 1.03 1.17
0.27 3.50 0.83
13.5 12.5 11.5 10.5 9.5 9.0 8.5 8.0 7.5 7.0 6.5 6.0 5.5 5.0 4.5 4.0 3.5 3.0 2.5 2.0 1.5 1.0 0.5 0.0 -0.5
f1 (ppm)

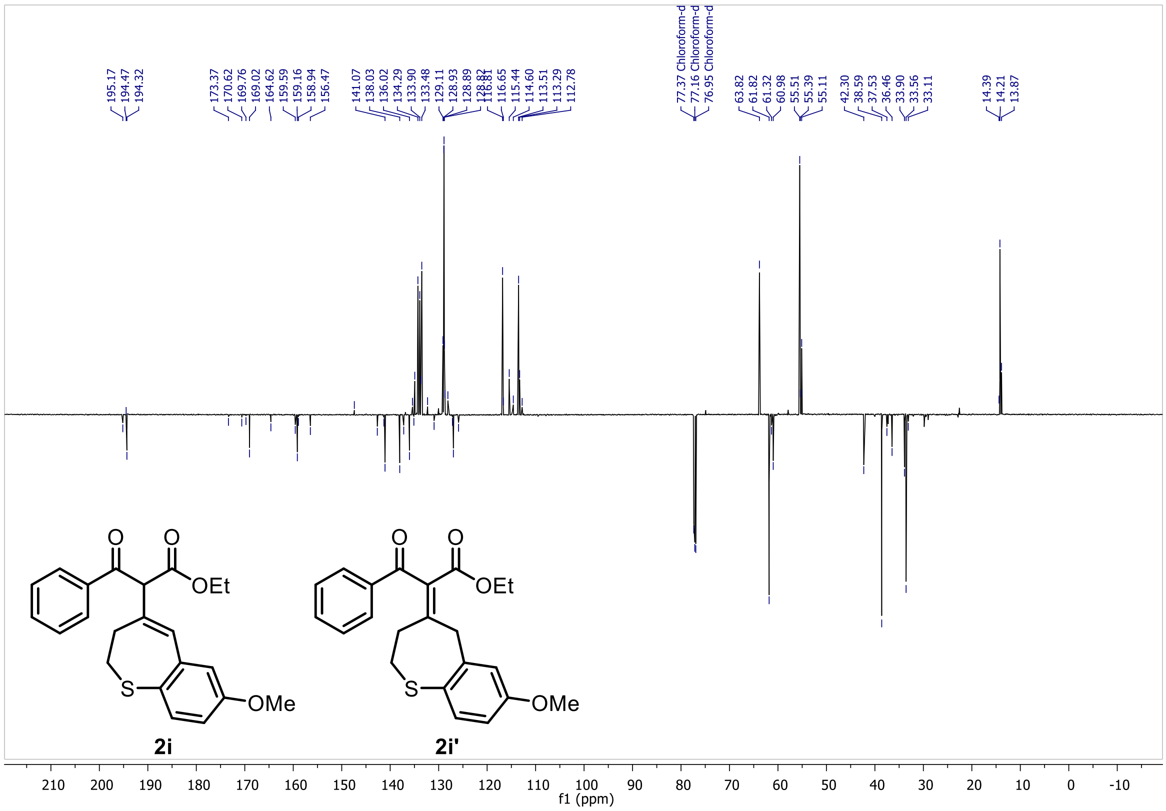
195.17 194.47 194.32
173.37 170.62 169.76 169.02 164.62 159.59 159.16 158.94 156.47
141.07 138.03 136.02 134.29 133.90 133.48 129.11 128.93 128.89 128.82 128.81 116.65 115.44 114.60 113.51 113.29 112.78
77.37 Chloroform-d 77.16 Chloroform-d 76.95 Chloroform-d
63.82 61.82 61.32 60.98 55.51 55.39 55.11
42.30 38.59 37.53 36.46 33.90 33.56 33.11
14.39 14.21 13.87
O
O
OEt
S
OMe
2i
2i'
210 200 190 180 170 160 150 140 130 120 110 100 90 80 70 60 50 40 30 20 10 0 -10
f1 (ppm)

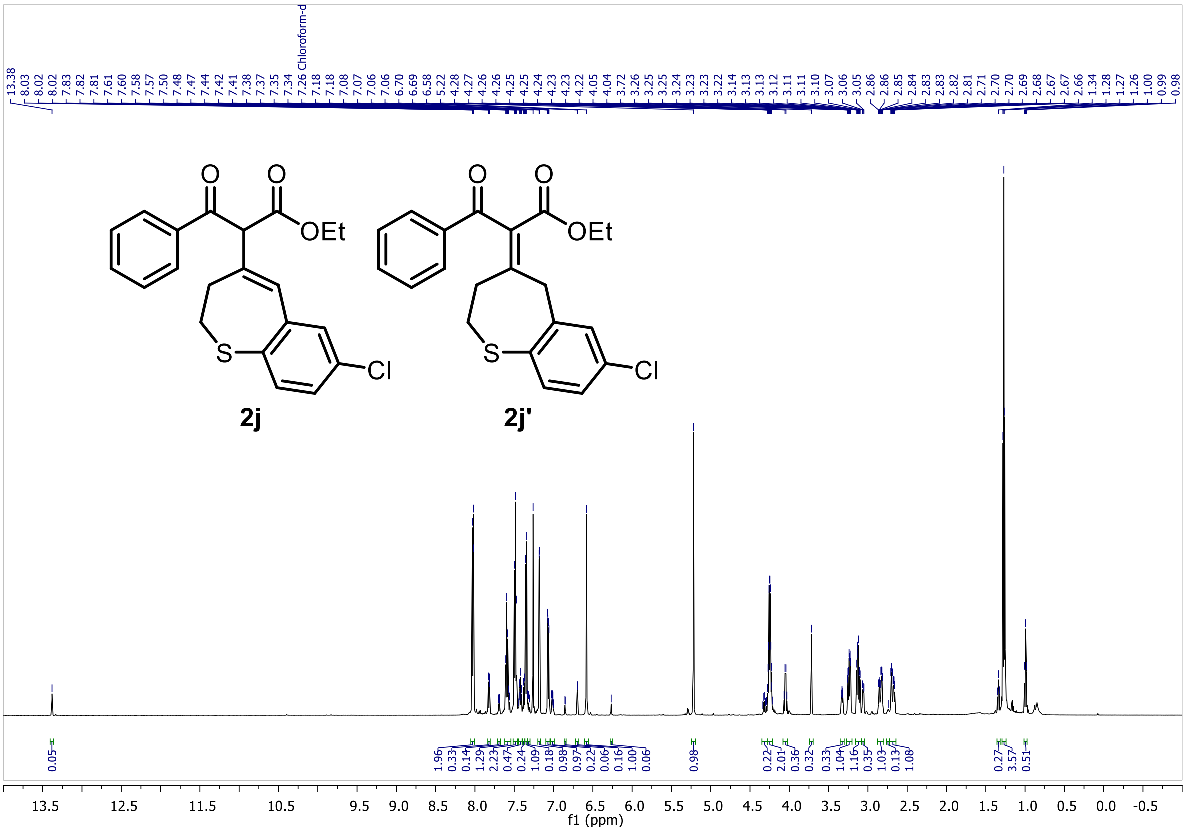

13.38
8.03
8.02
8.02
7.83
7.82
7.81
7.61
7.60
7.58
7.57
7.50
7.48
7.47
7.44
7.42
7.41
7.38
7.37
7.35
7.34
7.26 Chloroform-d
7.18
7.18
7.08
7.07
7.06
7.06
6.70
6.69
6.58
5.22
4.28
4.27
4.26
4.26
4.25
4.25
4.24
4.23
4.23
4.22
4.05
4.04
3.72
3.26
3.25
3.25
3.24
3.23
3.23
3.22
3.14
3.13
3.13
3.12
3.11
3.11
3.10
3.07
3.06
3.05
2.86
2.86
2.85
2.84
2.83
2.83
2.82
2.81
2.71
2.70
2.70
2.69
2.68
2.67
2.66
1.34
1.28
1.27
1.26
1.00
0.99
0.98
OEt
S
Cl
2j
2j'
0.05
1.96
0.33
0.14
1.29
2.23
0.47
0.24
1.09
0.18
0.98
0.97
0.22
0.06
0.16
1.00
0.06
0.98
0.22
2.01
0.36
0.32
0.33
1.04
1.16
0.35
1.03
0.13
1.08
0.27
3.57
0.51
13.5 12.5 11.5 10.5 9.5 9.0 8.5 8.0 7.5 7.0 6.5 6.0 5.5 5.0 4.5 4.0 3.5 3.0 2.5 2.0 1.5 1.0 0.5 0.0 -0.5
f1 (ppm)

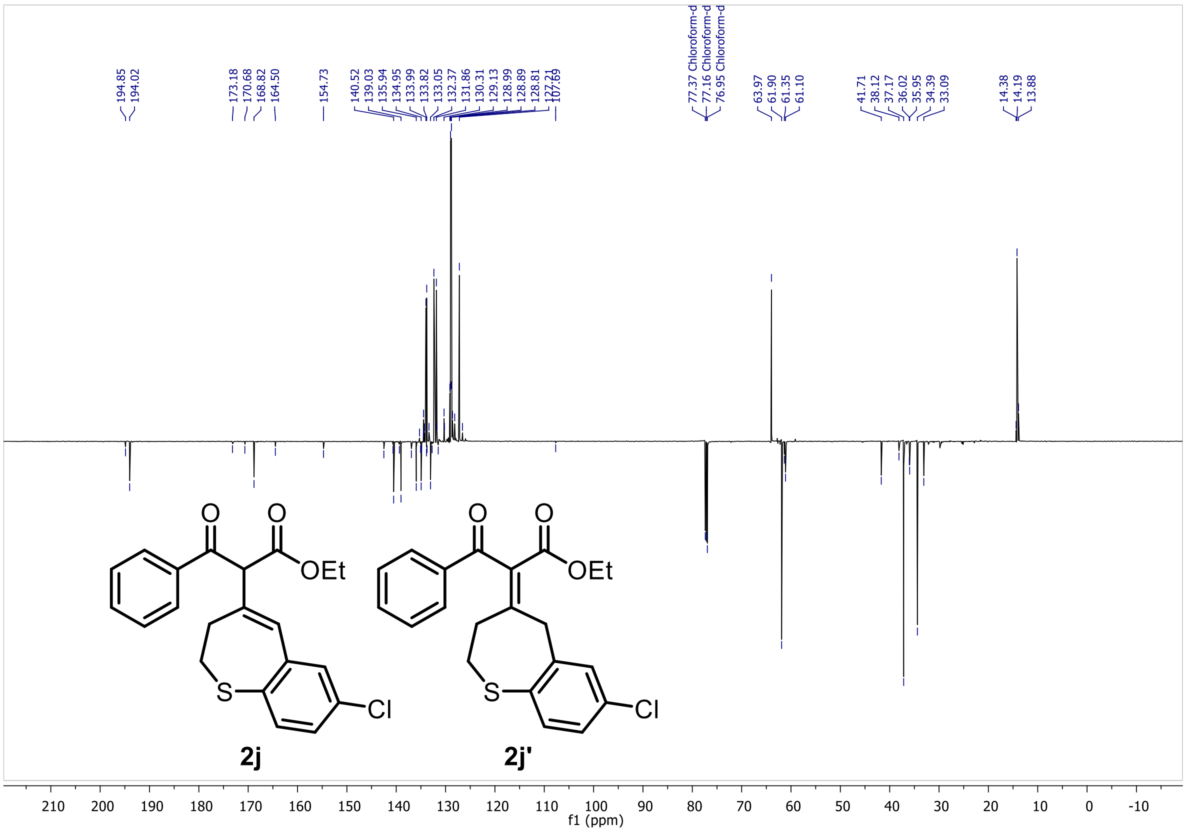

194.85
194.02
173.18
170.68
168.82
164.50
154.73
140.52
139.03
135.94
134.95
133.99
133.82
133.05
132.37
131.86
130.31
129.13
128.99
128.89
128.81
127.21
107.69
77.37 Chloroform-d
77.16 Chloroform-d
76.95 Chloroform-d
63.97
61.90
61.35
61.10
41.71
38.12
37.17
36.02
35.95
34.39
33.09
14.38
14.19
13.88
OEt
S
Cl
2j
2j'
210 200 190 180 170 160 150 140 130 120 110 100 90 80 70 60 50 40 30 20 10 0 -10
f1 (ppm)

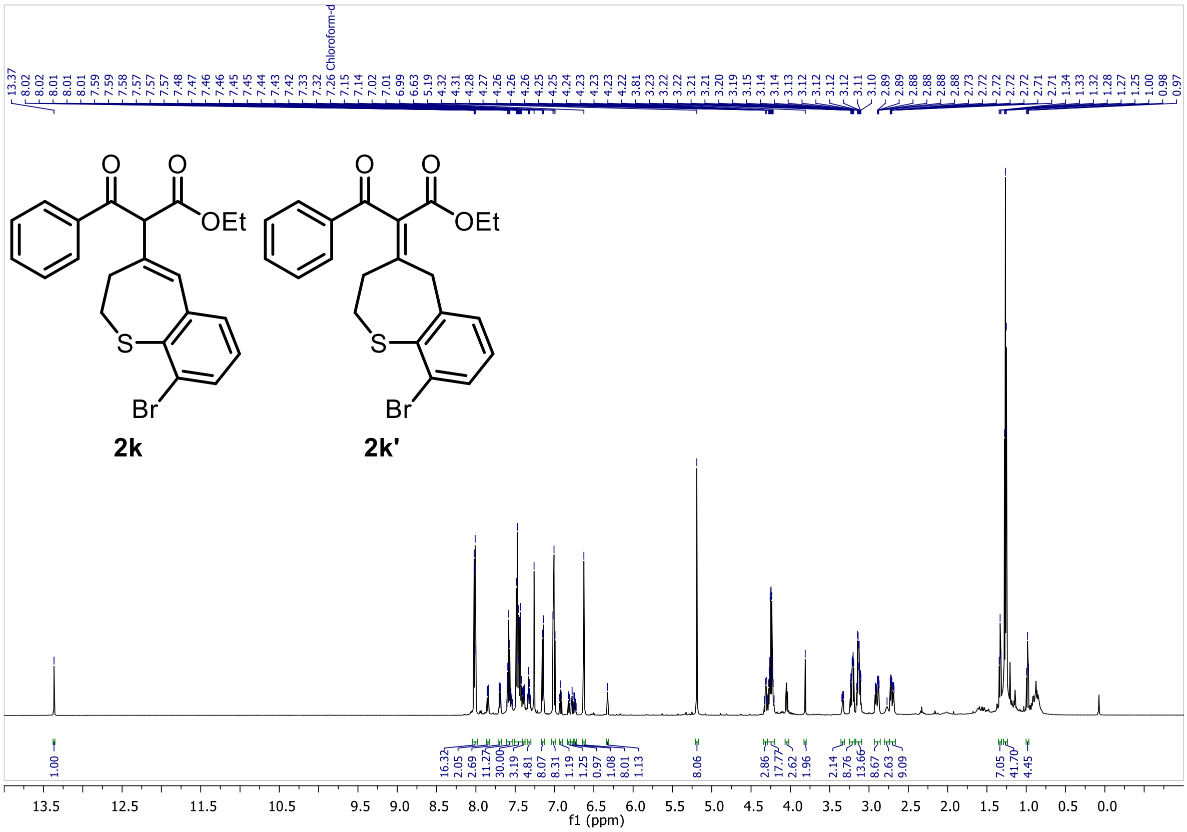
OEt
OEt
S
S
Br
Br
2k
2k'
f1 (ppm)

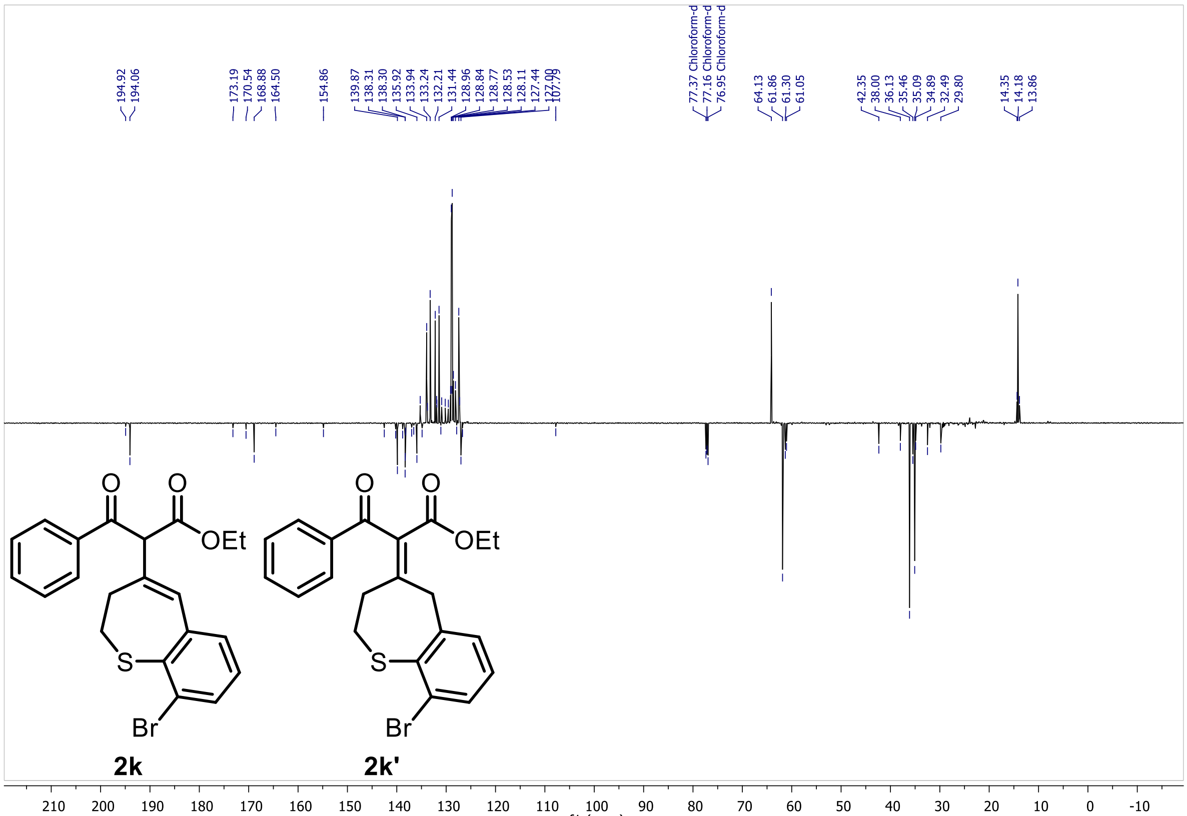
OEt
OEt
S
S
Br
Br
2k
2k'
f1 (ppm)

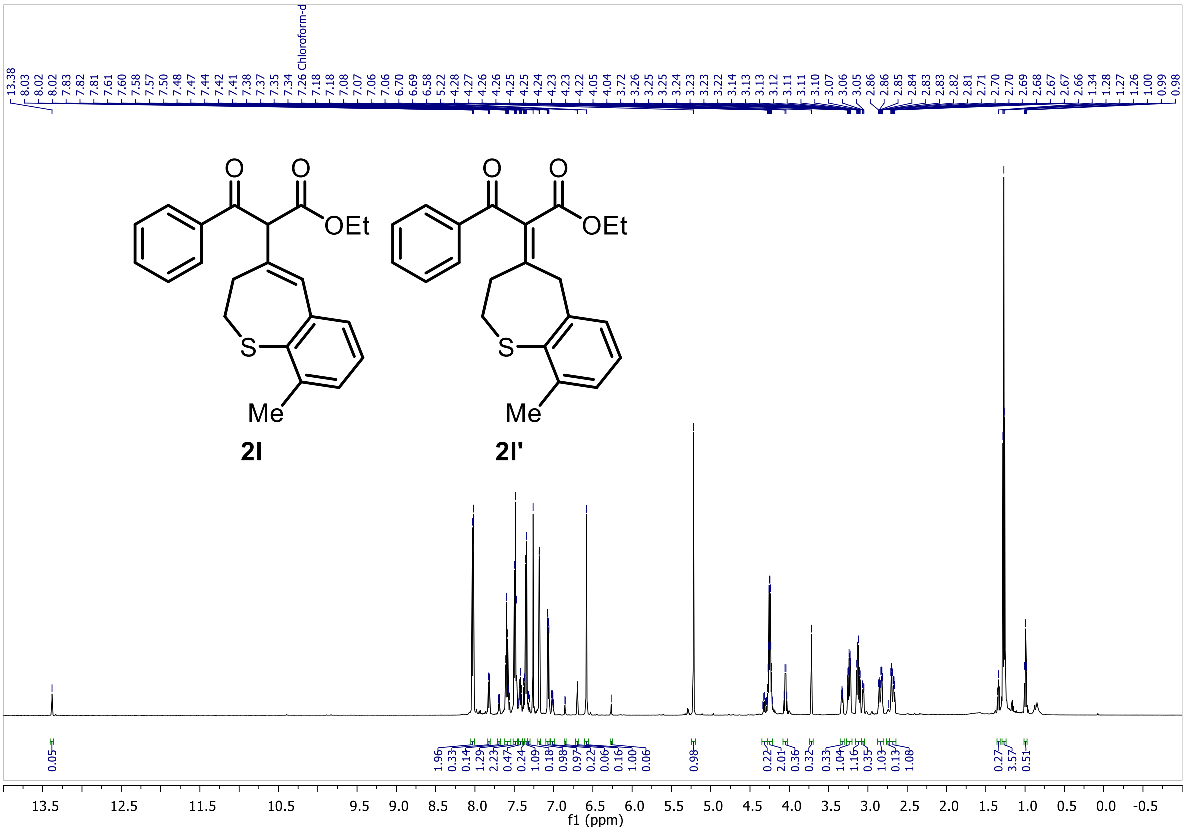

13.38 8.03 8.02 8.02 7.83 7.82 7.81 7.61 7.60 7.58 7.57 7.50 7.48 7.47 7.44 7.42 7.41 7.38 7.37 7.35 7.34 7.26 Chloroform-d 7.18 7.18 7.08 7.07 7.06 7.06 6.70 6.69 6.58 5.22 4.28 4.27 4.26 4.26 4.25 4.25 4.24 4.23 4.23 4.22 4.05 4.04 3.72 3.26 3.25 3.25 3.24 3.23 3.23 3.22 3.14 3.13 3.13 3.12 3.11 3.11 3.10 3.07 3.06 3.05 2.86 2.86 2.85 2.84 2.83 2.83 2.82 2.81 2.71 2.70 2.70 2.69 2.68 2.67 2.66 1.34 1.28 1.27 1.26 1.00 0.99 0.98
O
O
OEt
S
Me
2l
O
O
OEt
S
Me
2l'
0.05 1.96 0.33 0.14 1.29 2.23 0.47 0.24 1.09 0.18 0.98 0.97 0.22 0.06 0.16 1.00 0.06 0.98 0.22 2.01 0.36 0.32 0.33 1.04 1.16 0.35 1.03 0.13 1.08 0.27 3.57 0.51
13.5 12.5 11.5 10.5 9.5 9.0 8.5 8.0 7.5 7.0 6.5 6.0 5.5 5.0 4.5 4.0 3.5 3.0 2.5 2.0 1.5 1.0 0.5 0.0 -0.5
f1 (ppm)

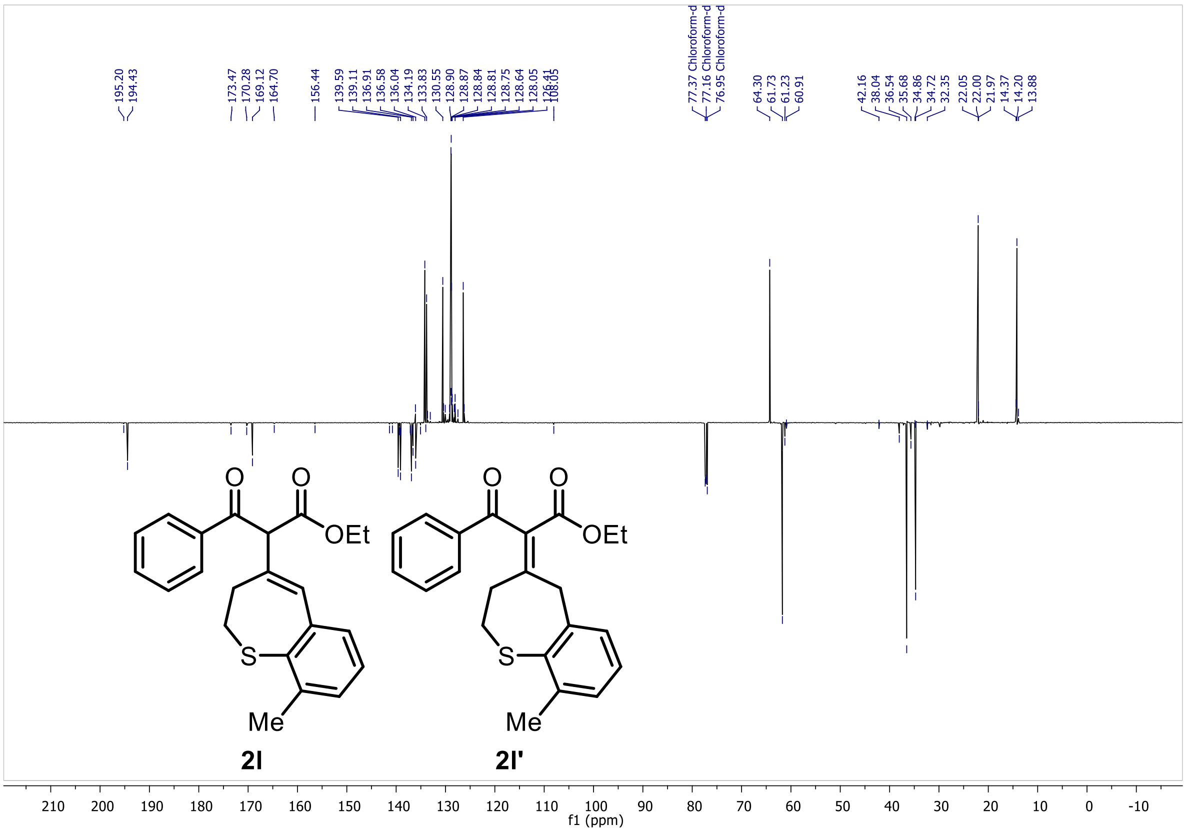

195.20 194.43 173.47 170.28 169.12 164.70 156.44 139.59 139.11 136.91 136.58 136.04 134.19 133.83 130.55 128.90 128.87 128.84 128.81 128.75 128.64 128.05 126.41 108.05 77.37 Chloroform-d 77.16 Chloroform-d 76.95 Chloroform-d 64.30 61.73 61.23 60.91 42.16 38.04 36.54 35.68 34.86 34.72 32.35 22.05 22.00 21.97 14.37 14.20 13.88
O
O
OEt
S
Me
2l
O
O
OEt
S
Me
2l'
210 200 190 180 170 160 150 140 130 120 110 100 90 80 70 60 50 40 30 20 10 0 -10
f1 (ppm)

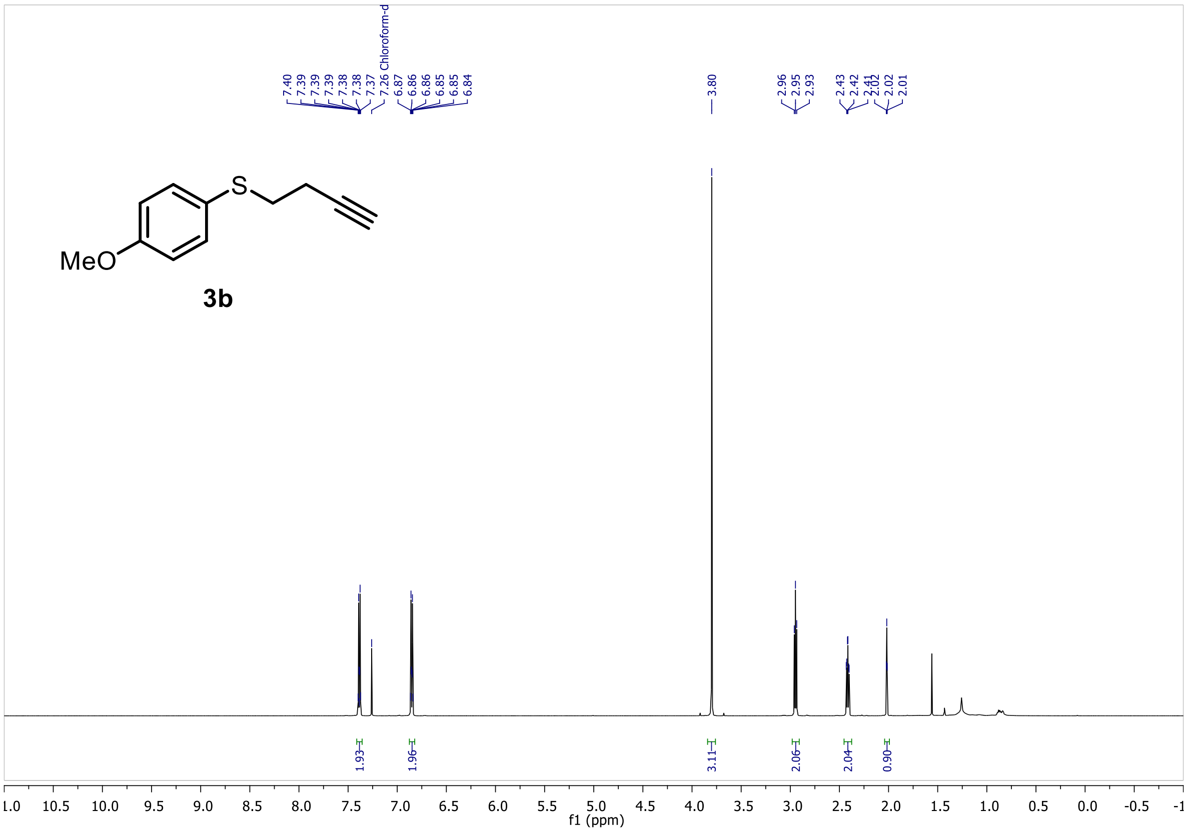
7.40
7.39
7.39
7.39
7.38
7.38
7.37
7.26 Chloroform-d
6.87
6.86
6.86
6.85
6.84
3.80
2.96
2.95
2.93
2.43
2.42
2.41
2.02
2.02
2.01
MeO
S
1.93
1.96
3.11
2.06
2.04
0.90
f1 (ppm)

3b

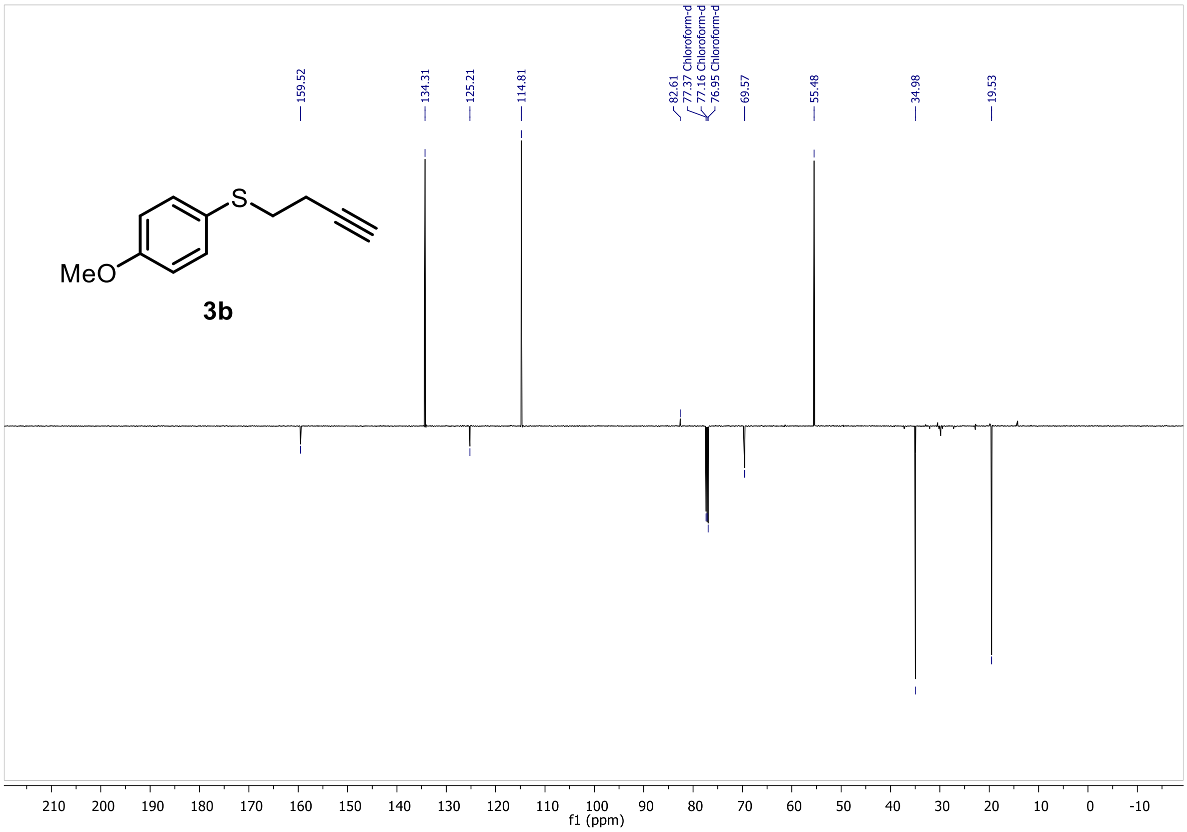
159.52
134.31
125.21
114.81
82.61
77.37 Chloroform-d
77.16 Chloroform-d
76.95 Chloroform-d
69.57
55.48
34.98
19.53
MeO
S
f1 (ppm)

3b

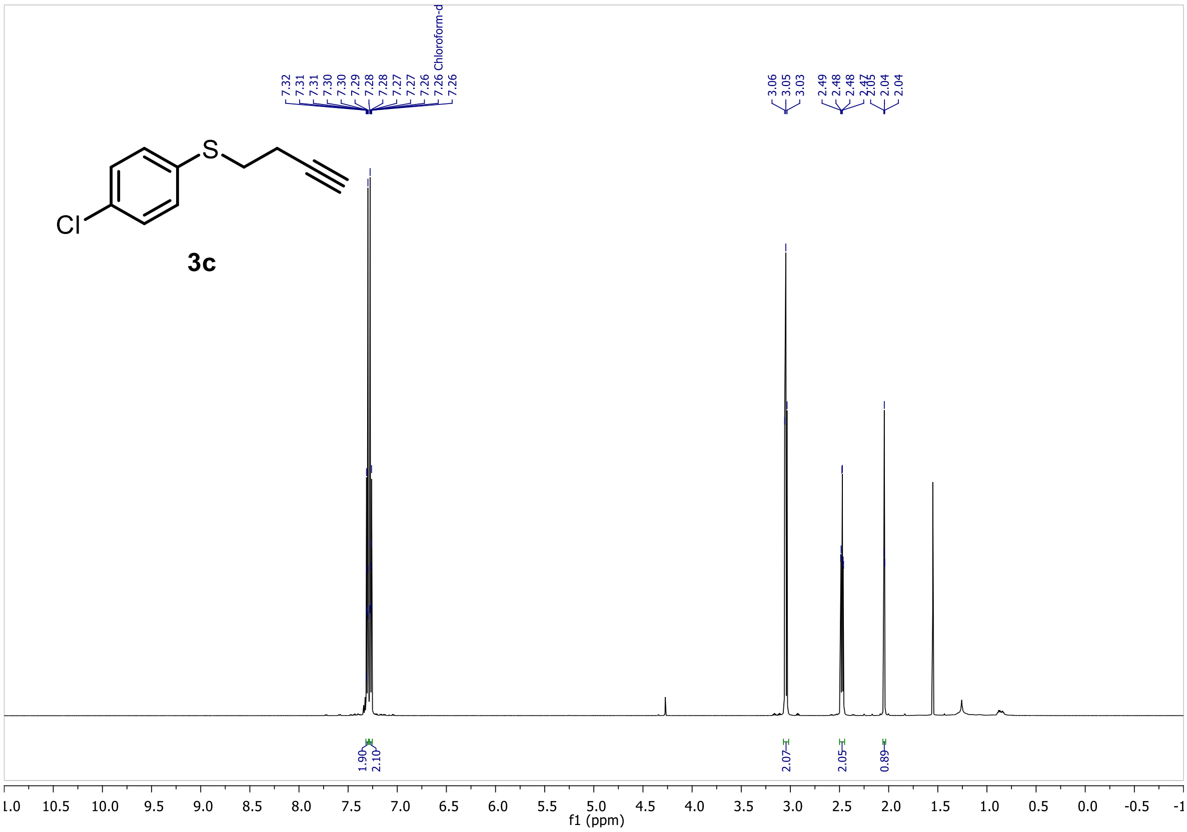
7.32
7.31
7.31
7.30
7.30
7.29
7.28
7.28
7.27
7.27
7.26
7.26 Chloroform-d
7.26
3.06
3.05
3.03
2.49
2.48
2.48
2.47
2.05
2.04
2.04
S
Cl
3c
1.90
2.10
2.07
2.05
0.89
f1 (ppm)

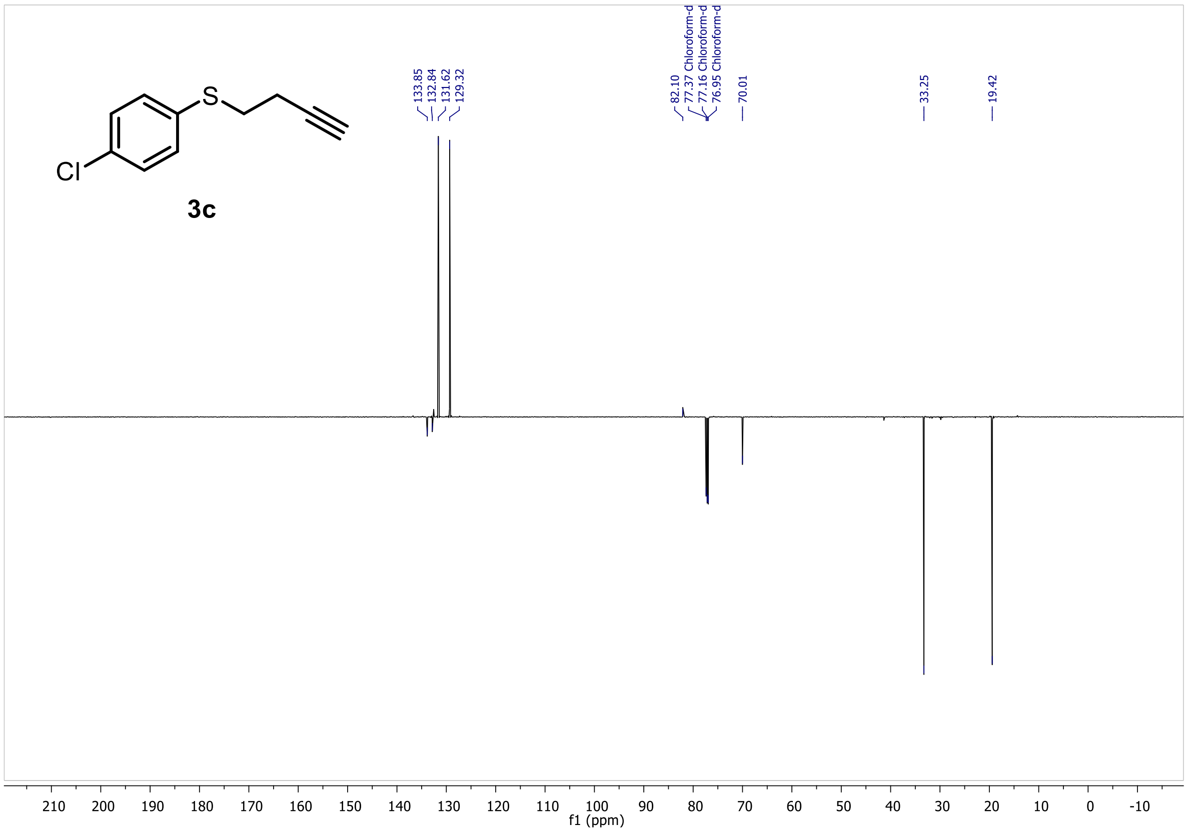
133.85
132.84
131.62
129.32
82.10
77.37 Chloroform-d
77.16 Chloroform-d
76.95 Chloroform-d
70.01
33.25
19.42
S
Cl
3c
f1 (ppm)

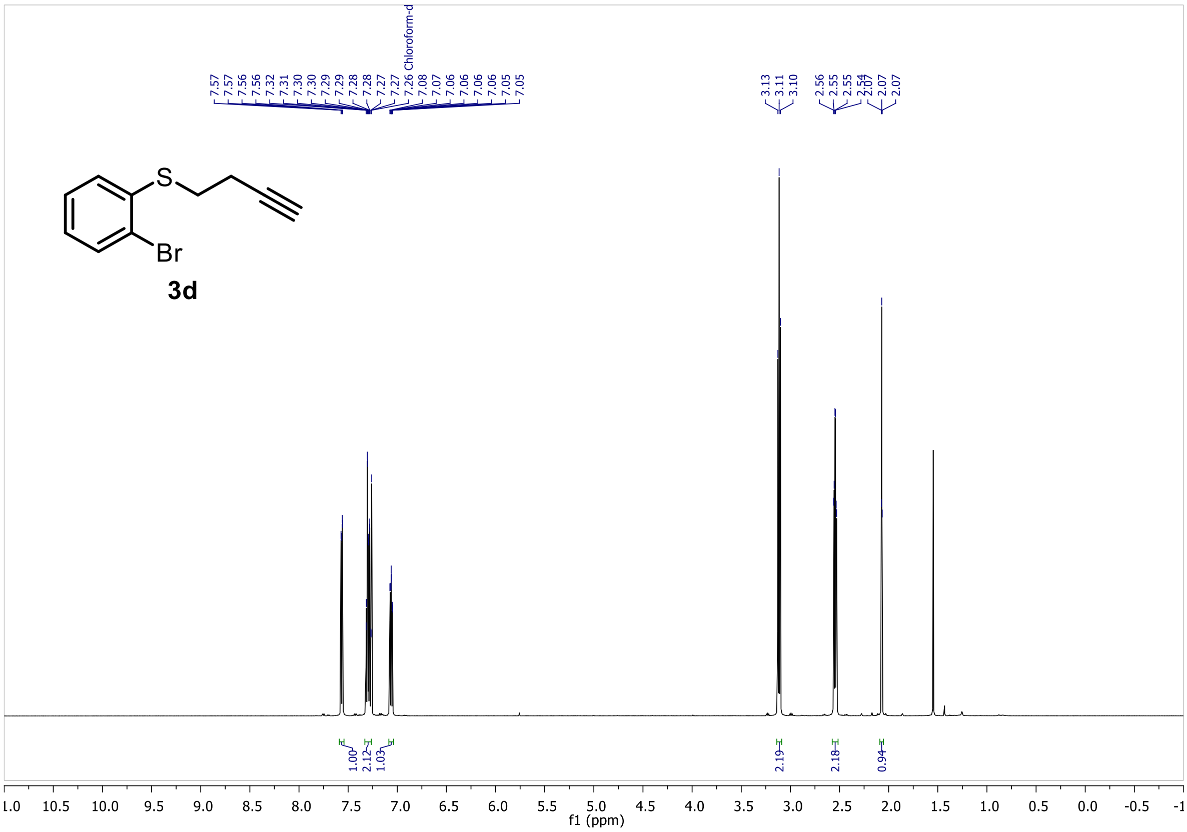
7.57 7.57 7.56 7.56 7.32 7.31 7.30 7.30 7.29 7.29 7.28 7.28 7.27 7.27 7.26 Chloroform-d 7.08 7.07 7.06 7.06 7.06 7.06 7.05 7.05
3.13 3.11 3.10
2.56 2.55 2.55 2.54 2.07 2.07 2.07
S
Br
3d
1.00 2.12 1.03
2.19 2.18 0.94
f1 (ppm)

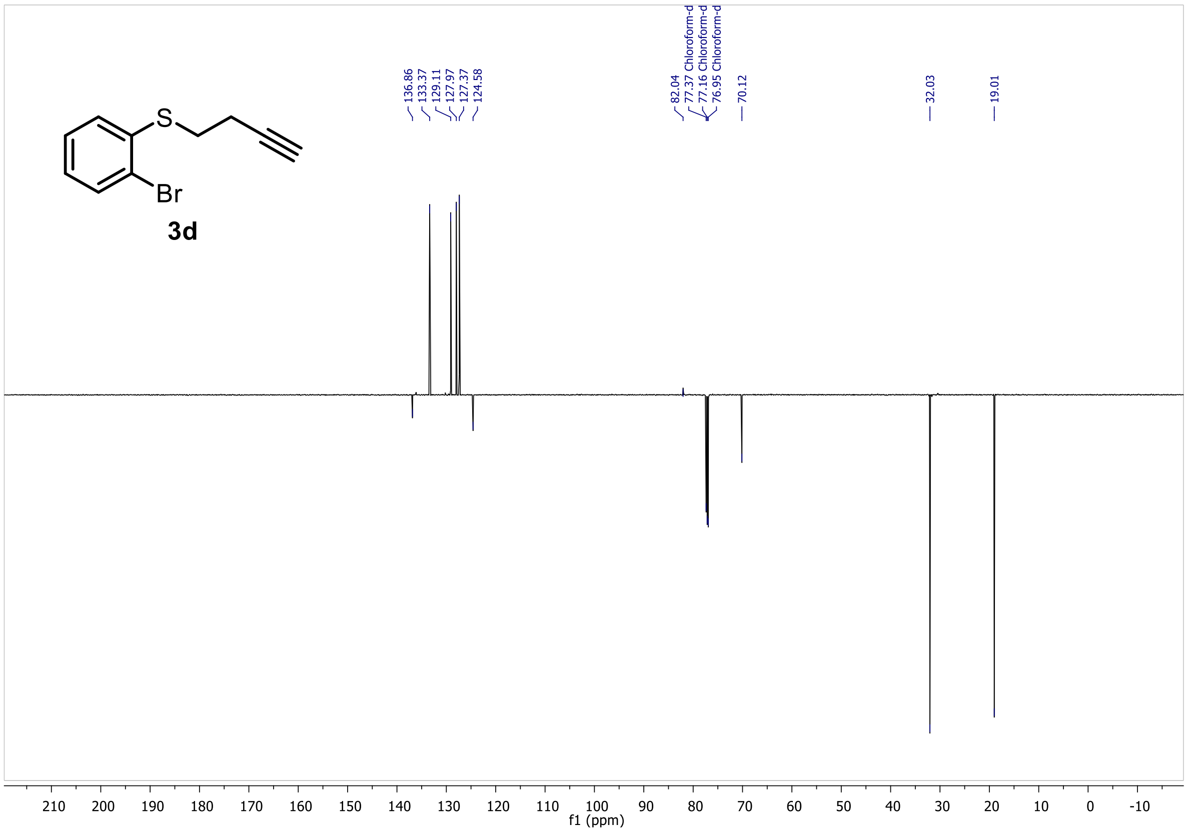
136.86 133.37 129.11 127.97 127.37 124.58
82.04 77.37 Chloroform-d 77.16 Chloroform-d 76.95 Chloroform-d
70.12
32.03
19.01
S
Br
3d
f1 (ppm)

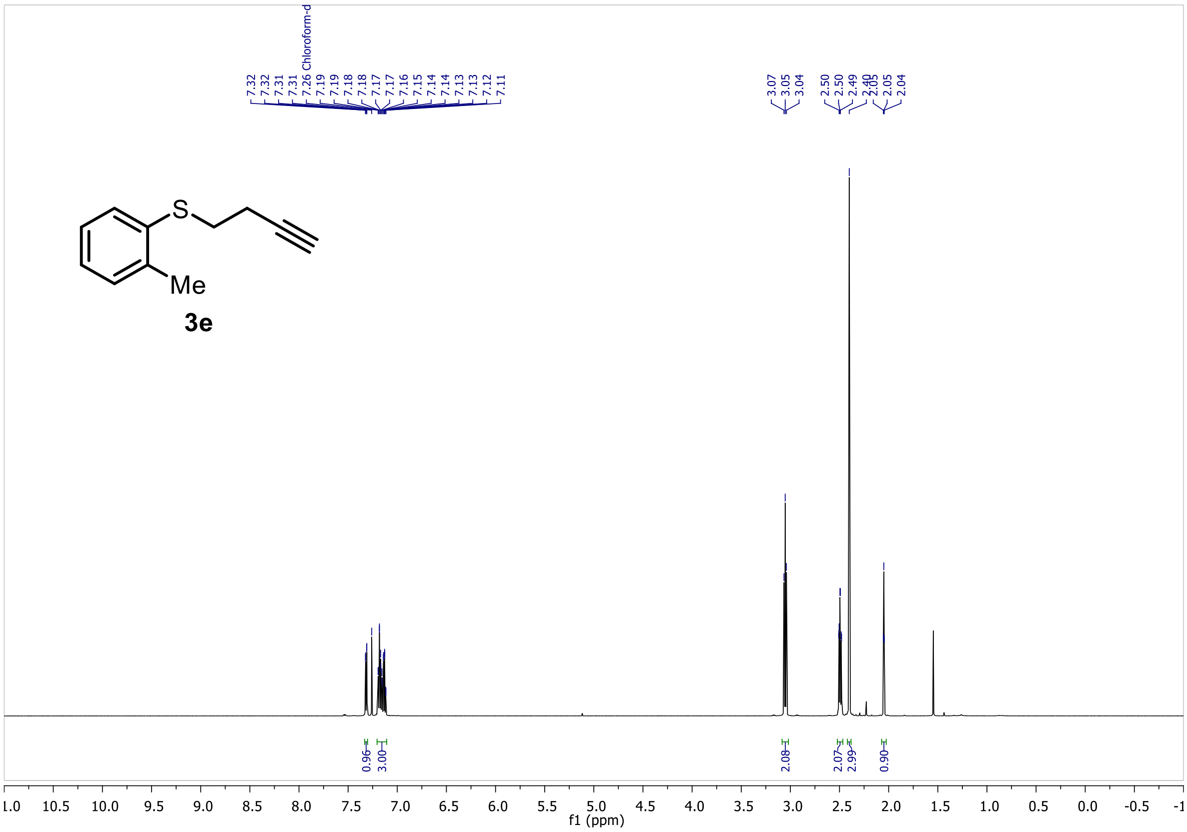

7.32
7.32
7.31
7.31
7.26 Chloroform-d
7.19
7.19
7.18
7.18
7.17
7.17
7.16
7.15
7.14
7.14
7.13
7.13
7.12
7.11
3.07
3.05
3.04
2.50
2.50
2.49
2.05
2.05
2.04
S
Me
3e
0.96
3.00
2.08
2.07
2.99
0.90
f1 (ppm)

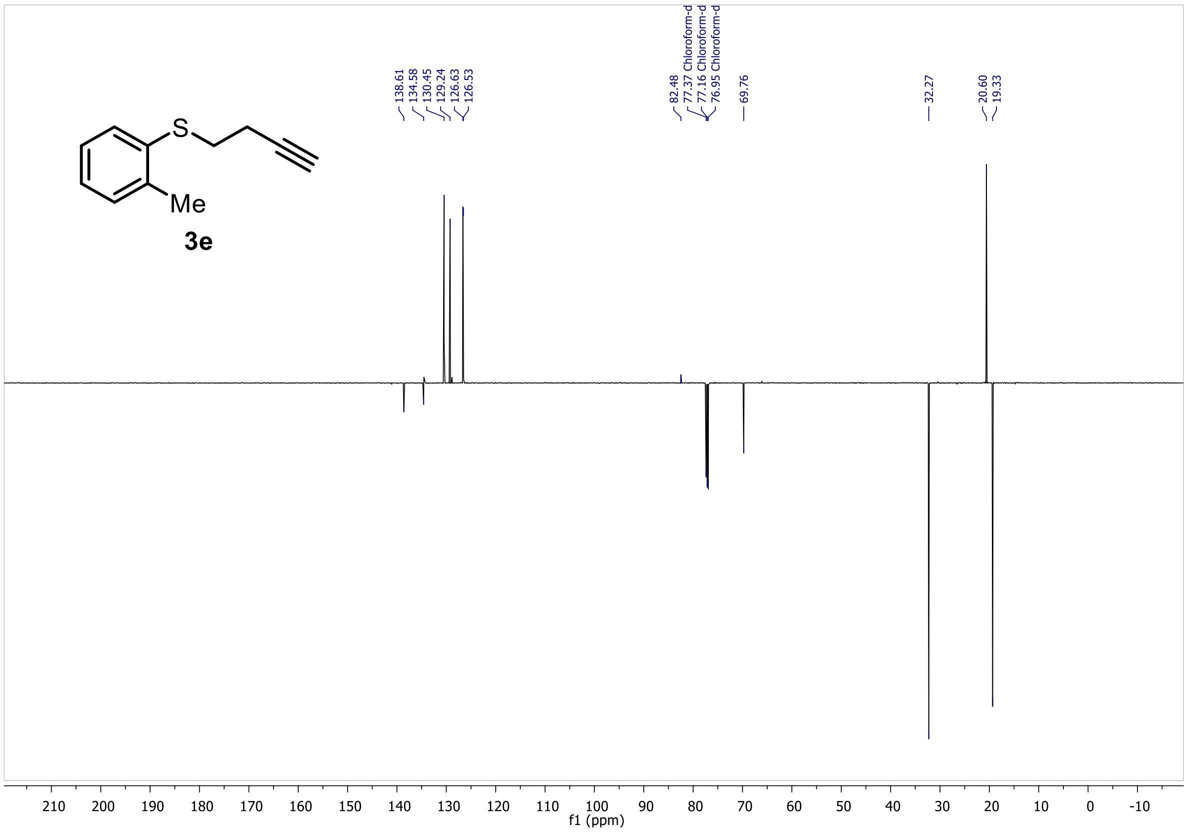

138.61
134.58
130.45
129.24
126.63
126.53
82.48
77.37 Chloroform-d
77.16 Chloroform-d
76.95 Chloroform-d
69.76
32.27
20.60
19.33
S
Me
3e
f1 (ppm)

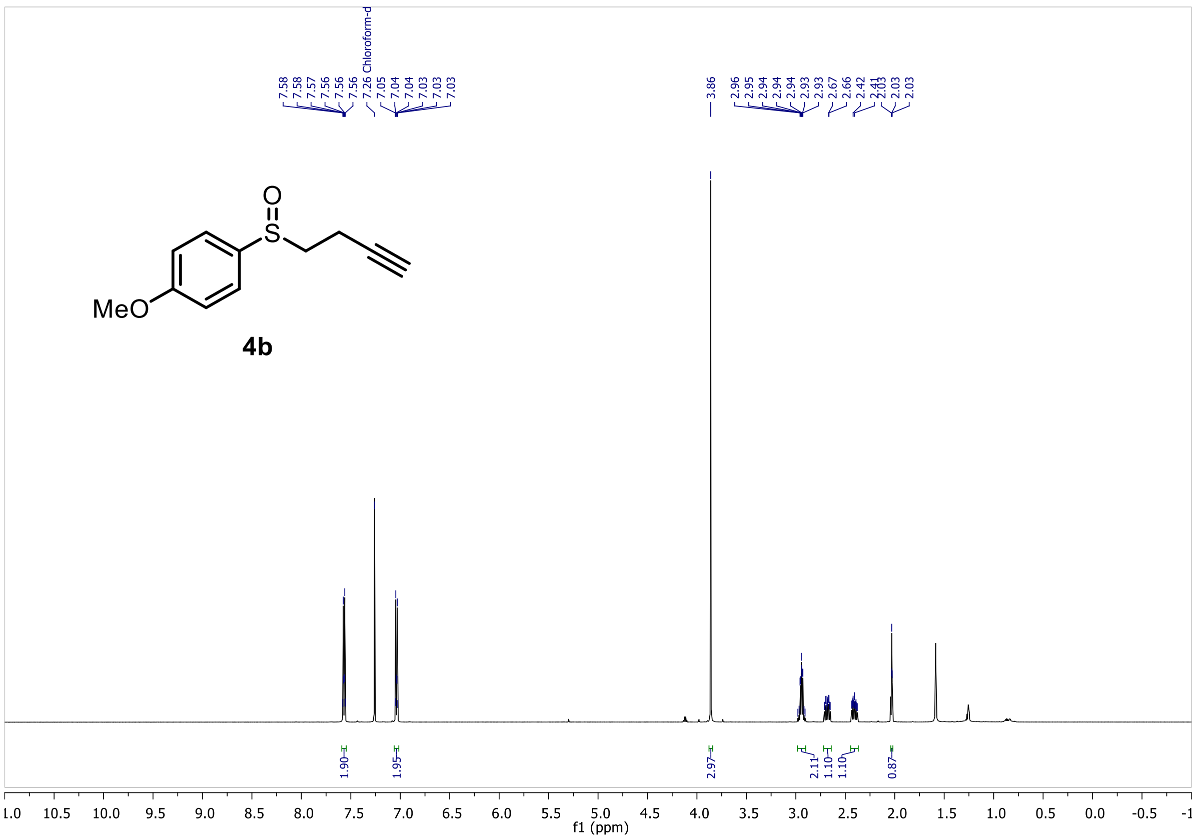
7.58
7.58
7.57
7.56
7.56
7.26 Chloroform-d
7.05
7.04
7.04
7.03
7.03
7.03
3.86
2.96
2.95
2.94
2.94
2.94
2.93
2.93
2.67
2.66
2.42
2.41
2.03
2.03
2.03
MeO
S
O
4b
1.90
1.95
2.97
2.11
1.10
1.10
0.87
f1 (ppm)

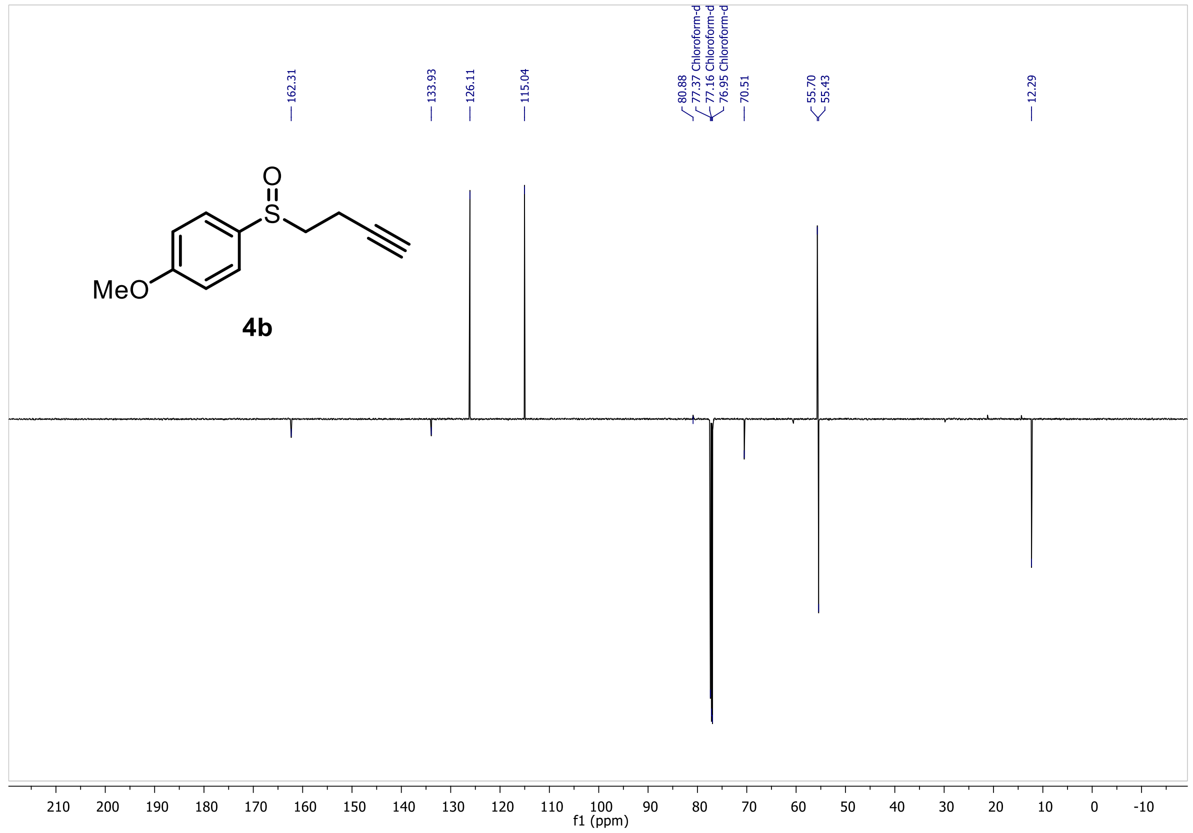
162.31
133.93
126.11
115.04
80.88
77.37 Chloroform-d
77.16 Chloroform-d
76.95 Chloroform-d
70.51
55.70
55.43
12.29
MeO
S
O
4b
f1 (ppm)

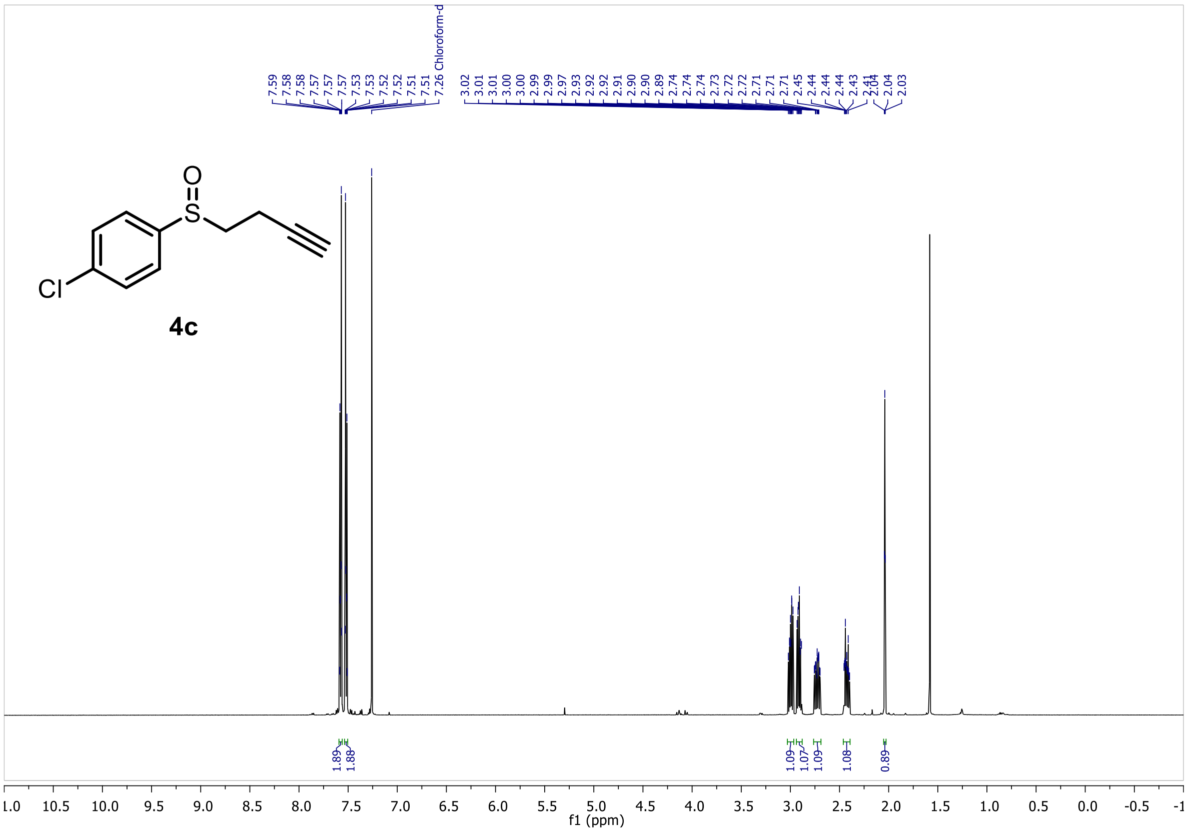
7.59
7.58
7.58
7.57
7.57
7.57
7.53
7.53
7.52
7.52
7.51
7.51
7.26 Chloroform-d
3.02
3.01
3.01
3.00
3.00
2.99
2.99
2.97
2.93
2.92
2.92
2.91
2.90
2.90
2.89
2.74
2.74
2.73
2.72
2.72
2.71
2.71
2.71
2.45
2.44
2.44
2.44
2.43
2.41
2.04
2.04
2.03
O
S
Cl
4c
1.89
1.88
1.09
1.07
1.09
1.08
0.89
f1 (ppm)

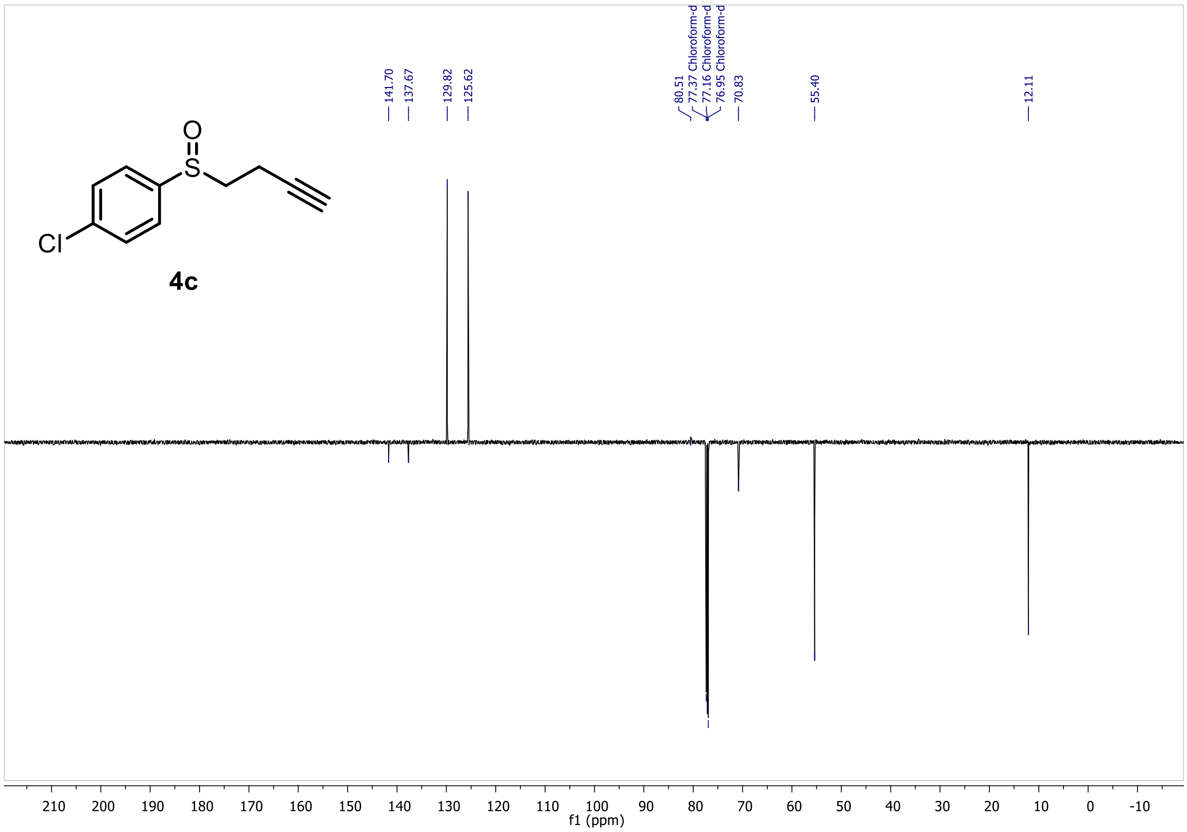
141.70
137.67
129.82
125.62
80.51
77.37 Chloroform-d
77.16 Chloroform-d
76.95 Chloroform-d
70.83
55.40
12.11
O
S
Cl
4c
f1 (ppm)

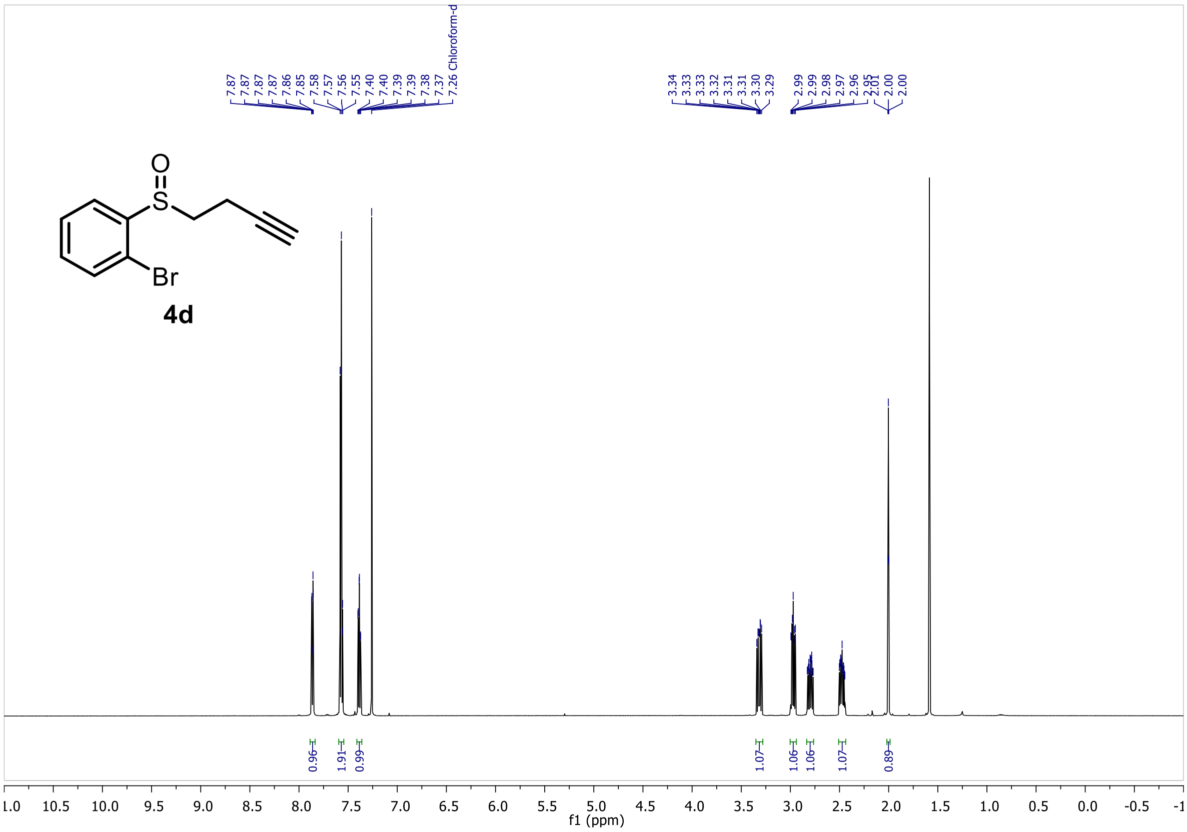
7.87
7.87
7.87
7.87
7.86
7.85
7.58
7.57
7.56
7.55
7.40
7.40
7.39
7.39
7.38
7.37
7.26 Chloroform-d
3.34
3.33
3.33
3.32
3.31
3.31
3.30
3.29
2.99
2.99
2.98
2.97
2.96
2.95
2.01
2.00
2.00
O
S
Br
4d
0.96
1.91
0.99
1.07
1.06
1.06
1.07
0.89
f1 (ppm)

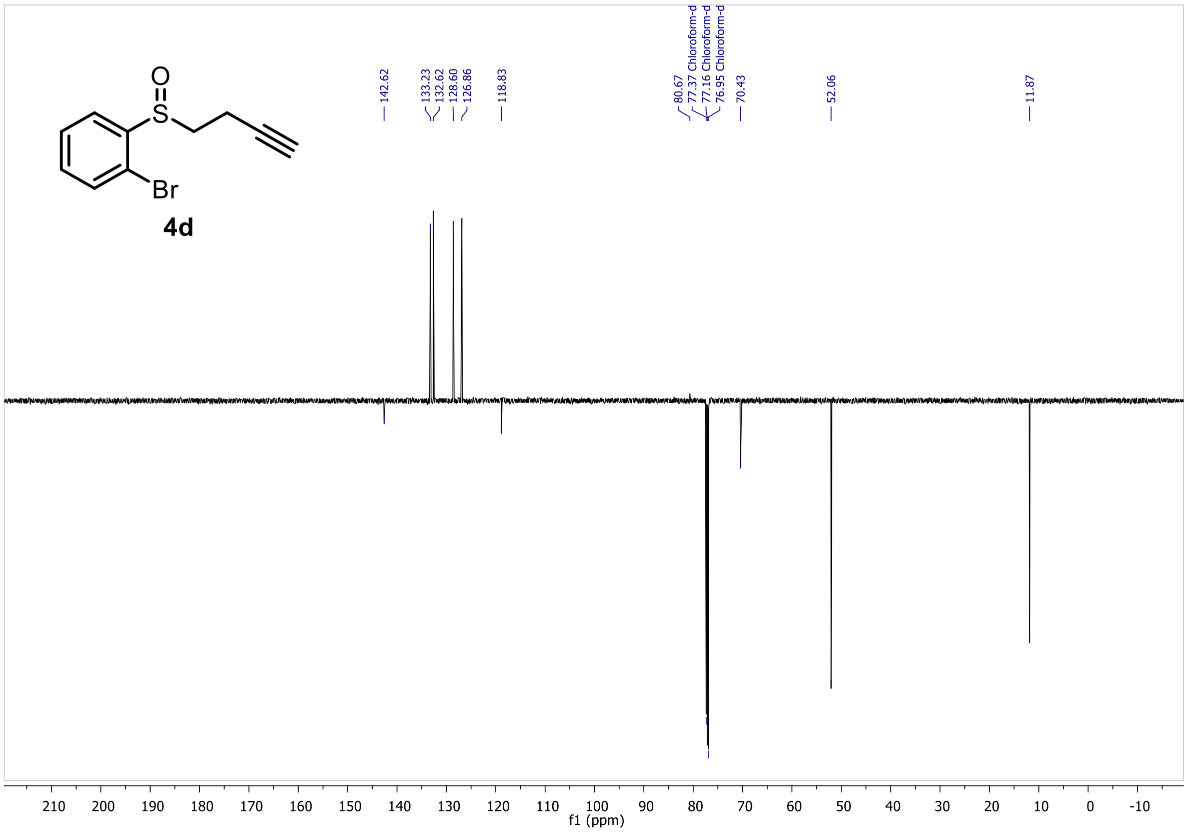
142.62
133.23
132.62
128.60
126.86
118.83
80.67
77.37 Chloroform-d
77.16 Chloroform-d
76.95 Chloroform-d
70.43
52.06
11.87
O
S
Br
4d
f1 (ppm)

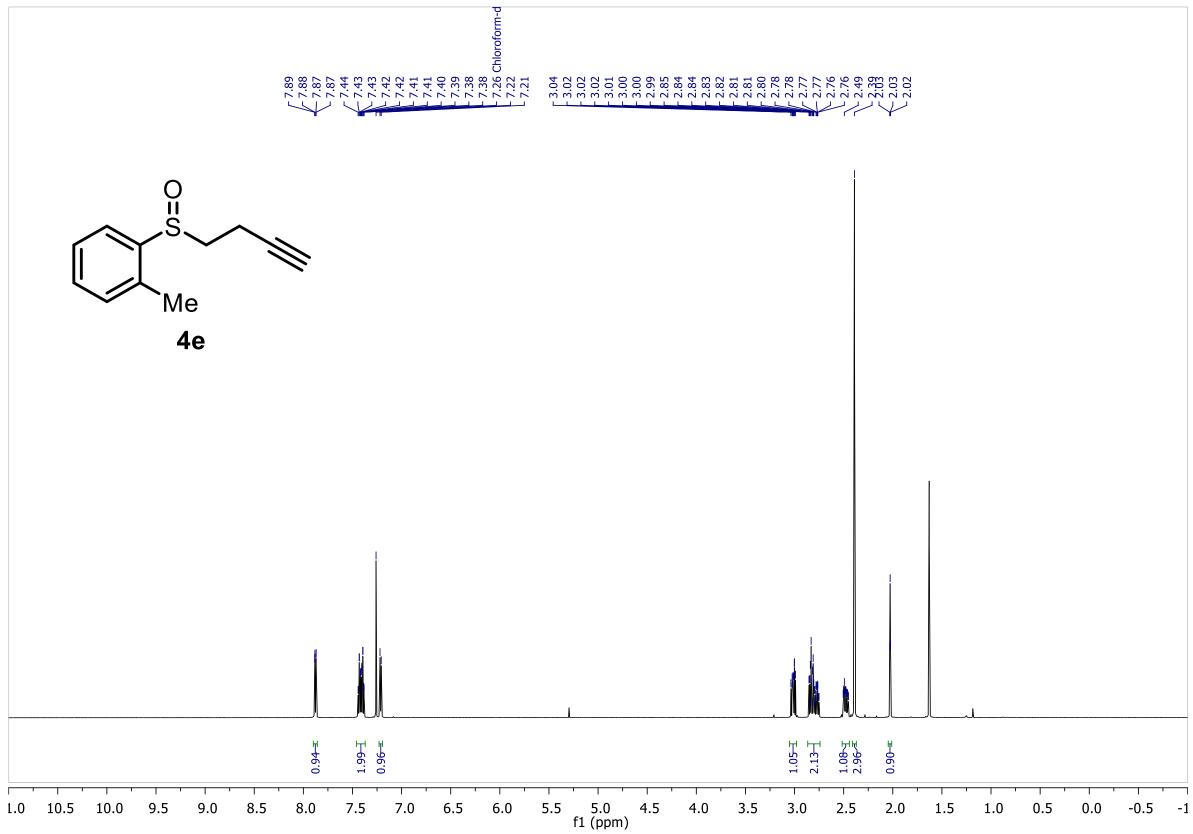
7.89
7.88
7.87
7.87
7.44
7.43
7.43
7.42
7.42
7.41
7.41
7.40
7.39
7.38
7.38
7.26 Chloroform-d
7.22
7.21
3.04
3.02
3.02
3.01
3.00
3.00
2.99
2.85
2.84
2.84
2.83
2.82
2.81
2.81
2.80
2.78
2.78
2.77
2.77
2.76
2.76
2.49
2.39
2.03
2.02
O
S
Me
4e
0.94
1.99
0.96
1.05
2.13
1.08
2.96
0.90
f1 (ppm)

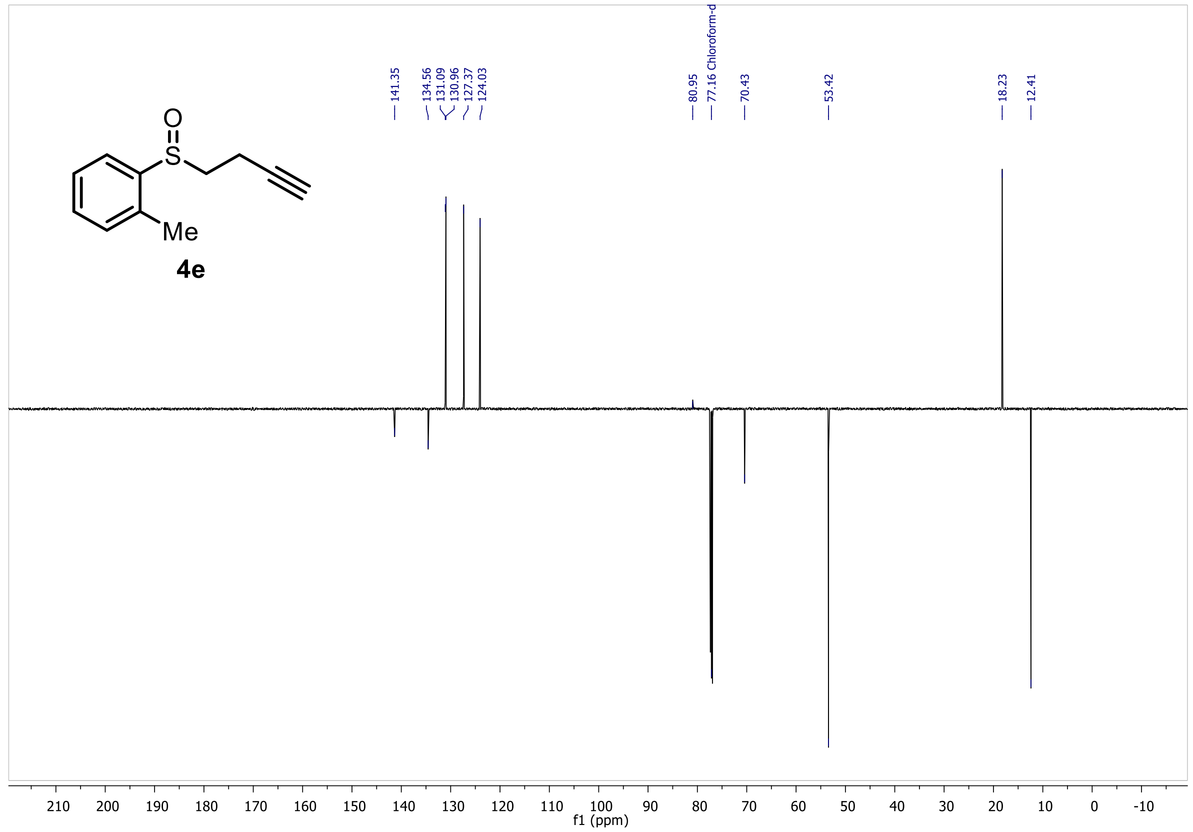
141.35
134.56
131.09
130.96
127.37
124.03
80.95
77.16 Chloroform-d
70.43
53.42
18.23
12.41
O
S
Me
4e
f1 (ppm)
